# Accuracy of Physicians Interpreting Photoplethysmography and Electrocardiography Tracings to Detect Atrial Fibrillation: INTERPRET-AF

**DOI:** 10.3389/fcvm.2021.734737

**Published:** 2021-09-20

**Authors:** Henri Gruwez, Stijn Evens, Tine Proesmans, David Duncker, Dominik Linz, Hein Heidbuchel, Martin Manninger, Pieter Vandervoort, Peter Haemers, Laurent Pison

**Affiliations:** ^1^Department of Cardiology, Hospital East-Limburg, Genk, Belgium; ^2^Department of Cardiovascular Sciences, University of Leuven, Leuven, Belgium; ^3^Doctoral School of Medicine and Life Science, Hasselt University, Hasselt, Belgium; ^4^Qompium NV, Hasselt, Belgium; ^5^Hannover Heart Rhythm Center, Department of Cardiology and Angiology, Hannover Medical School, Hanover, Germany; ^6^Department of Cardiology, Maastricht University Medical Centre and Cardiovascular Research Institute Maastricht, Maastricht, Netherlands; ^7^Department of Cardiology, Antwerp University Hospital and Antwerp University, Antwerp, Belgium; ^8^Department of Cardiology, Medical University of Graz, Graz, Austria

**Keywords:** atrial fibrillaiton, single-lead ECG, PPG (photoplethysmography), digital health, electrocardiography

## Abstract

**Aims:** This study aims to compare the performance of physicians to detect atrial fibrillation (AF) based on photoplethysmography (PPG), single-lead ECG and 12-lead ECG, and to explore the incremental value of PPG presentation as a tachogram and Poincaré plot, and of algorithm classification for interpretation by physicians.

**Methods and Results:** Email invitations to participate in an online survey were distributed among physicians to analyse almost simultaneously recorded PPG, single-lead ECG and 12-lead ECG traces from 30 patients (10 in sinus rhythm (SR), 10 in SR with ectopic beats and 10 in AF). The task was to classify the readings as ‘SR', ‘ectopic/missed beats', ‘AF', ‘flutter' or ‘unreadable'. Sixty-five physicians detected or excluded AF based on the raw PPG waveforms with 88.8% sensitivity and 86.3% specificity. Additional presentation of the tachogram plus Poincaré plot significantly increased sensitivity and specificity to 95.5% (*P* < 0.001) and 92.5% (*P* < 0.001), respectively. The algorithm information did not further increase the accuracy to detect AF (sensitivity 97.5%, *P* = 0.556; specificity 95.0%, *P* = 0.182). Physicians detected AF on single-lead ECG tracings with 91.2% sensitivity and 93.9% specificity. Diagnostic accuracy was also not optimal on full 12-lead ECGs (93.9 and 98.6%, respectively). Notably, there was no significant difference between the performance of PPG waveform plus tachogram and Poincaré, compared to a single-lead ECG to detect or exclude AF (sensitivity *P* = 0.672; specificity *P* = 0.536).

**Conclusion:** Physicians can detect AF on a PPG output with equivalent accuracy compared to single-lead ECG, if the PPG waveforms are presented together with a tachogram and Poincaré plot and the quality of the recordings is high.

## Introduction

Atrial fibrillation (AF) is the most common sustained cardiac arrhythmia with an estimated number of 30–100 million patients worldwide (1). Currently, the prevalence of AF in Europe is approximated between 2 and 4% and is expected to double from 2010 to 2060 as a result of the increasing burden of risk factors such as hypertension, diabetes, and aging of the population (2, 3). AF is associated with significant morbidity including a 5-fold risk to develop stroke, increased heart failure rate, frequent hospitalizations and impaired quality of life, resulting in an overall 3.5-fold increase in mortality (3). According to the 2020 European Society of Cardiology (ESC) guidelines, the diagnosis of AF should be made on a standard 12-lead ECG or a ≥30 s single-lead ECG, showing an irregularly irregular rhythm, with no discernible P-waves preceding the QRS complexes (3). However, frequent or long term ECG monitoring is cumbersome and photoplethysmography (PPG) has emerged as a non-intrusive modality to monitor the heart rate and rhythm. A variety of mobile devices, including smartphones and smartwatches, enable PPG-based heart rhythm monitoring through their built-in cameras and/or photodetectors (4). PPG is an optical measurement technique, based on a pulse volume signal resulting from the propagation of blood pressure waves along arterial blood vessels (5). The data collected by PPG-based smartphone applications can also be used to generate a PPG waveform and various graphs that represent the interval between consecutive heartbeats to facilitate physician interpretation of the PPG output. Several algorithms have been developed to use PPG information to detect AF with a high sensitivity and specificity (6). However, data on the performance of physicians to accurately detect AF based on PPG output is lacking. This study aims to, to systematically determine and compare the accuracy of qualitative PPG, single-lead ECG and 12-lead ECG analysis by physicians to differentiate between AF and non-AF rhythms. Secondly, this study aims to explore the incremental value of PPG presentation as a tachogram and Poincaré plot, and of algorithm classification for interpretation by physicians. Thirdly, this study aims to evaluate the influence of prior PPG experience.

## Methods

### Study Design

In this prospective comparative study, cardiologists, electrophysiologists and cardiology fellows were invited via email to qualitatively analyse PPG, single-lead ECG, and 12-lead ECG recordings (each temporally related in the same patients) via three separate surveys. Demographic and professional characteristics were collected from all subjects. The study was performed between March 2020 and November 2020. The protocol complies with the Declaration of Helsinki and was approved by the local ethics committee (Ziekenhuis Oost-Limburg, Genk, Belgium). The study was registered at clinicaltrials.gov (NCT04374344).

### Survey Construction

The presented heart rhythm recordings were collected from a pre-existing dataset containing almost simultaneously recorded PPG, single-lead ECG and 12-lead ECG waveforms of patients that visited the outpatient cardiology department of the hospital ‘Ziekenhuis-Oost Limburg'. A 60-s PPG waveform using FibriCheck® (Qompium NV, Hasselt, Belgium), a 30-s single-lead ECG representing lead one using KardiaMobile® (AliveCor, Mountain View, USA) and 10-s standard 12-lead ECG using General Electric MAC 5500HD/VU360® (Boston, Massachusetts, USA) were collected consecutively during an outpatient consultation. The recordings from patients with sinus rhythm (SR), SR with ectopic atrial or ventricular beats and AF were exported as three separate datasets. Thirty patients were selected from the dataset based on the following criteria: sufficient quality of the PPG waveform according to the FibriCheck® algorithm, sufficient quality of the single-ECG recording according to the KardiaMobile® algorithm and visually classified as high-quality PPG, single-lead ECG and 12-lead ECG recordings by two blinded medical technicians. To provide the reference diagnosis, the 12-lead ECG recordings were additionally reviewed by two independent cardiologists. In case of disagreement, a third cardiologist was consulted. As a result, the collected data included high-quality recordings from 10 patients with a regular rhythm, 10 patients with SR with ectopic beats and 10 patients with AF.

**Figure 1:**
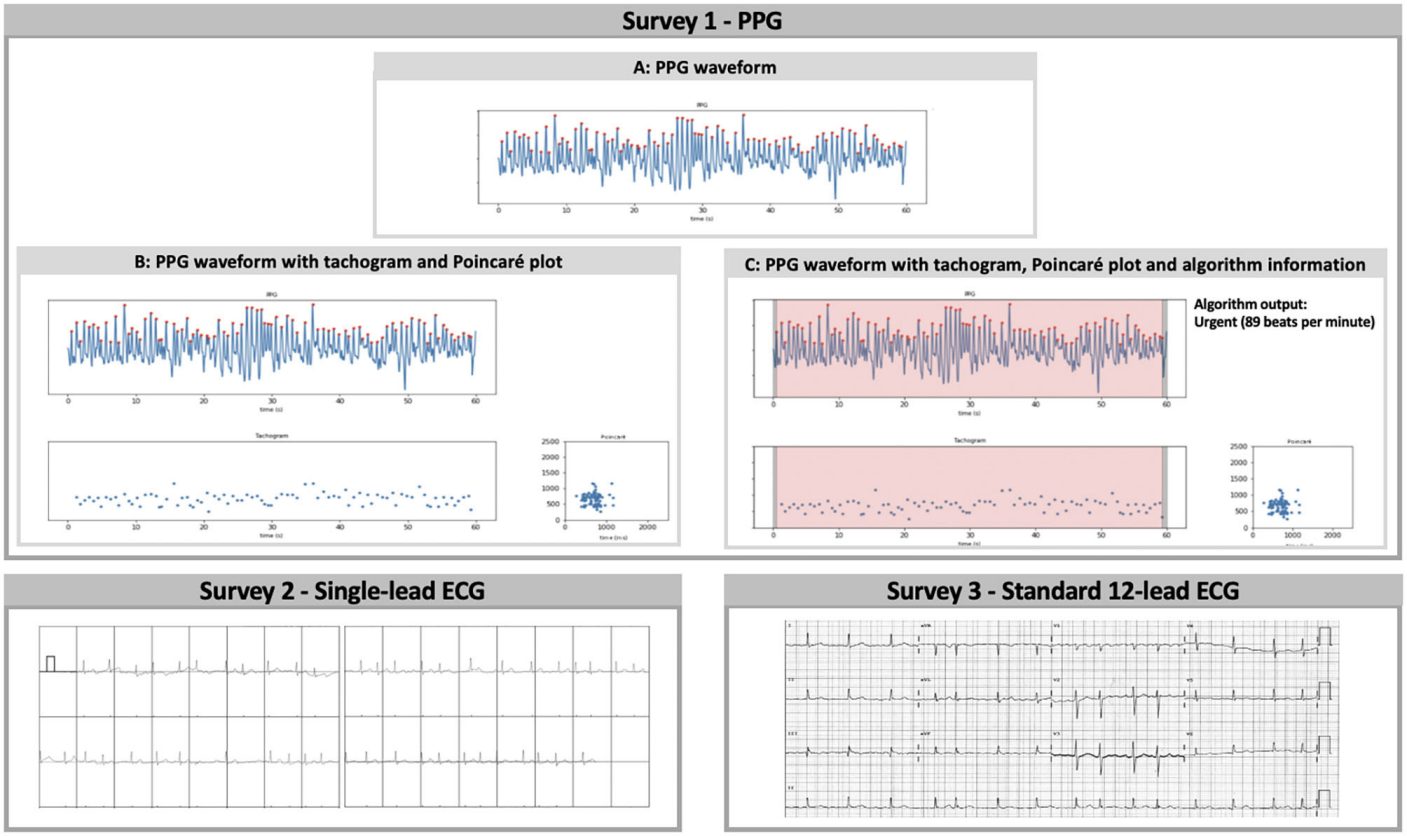
Survey 1: **(A)** PPG waveform, **(B)** PPG waveform with tachogram and Poincaré plot, and **(C)** PPG waveform with tachogram, Poincaré plot, and algorithm information were separately and consecutively shown for qualitative analysis in the first survey. The tachogram shows the duration of each peak-to-peak interval in milliseconds, while the Poincaré plot visualises the randomness of the heart rhythm by plotting each interval on the *x*-axis (ms) vs. the preceding interval on the *y*-axis (ms). The proprietary algorithm classified measurements with sufficient signal quality as normal (regular rhythm, green), warning (possible non-AF irregularity, orange), or urgent (possible AF, red). Survey 2: The performance of single-lead ECG analysis was assessed via the second survey. The vertical dashes underneath the QRS-complexes represent potential heartbeats detected by the Kardiamobile® algorithm. Survey 3: Finally, subjects were invited to qualitatively analyse traditional 12-lead ECG recordings in the third survey. PPG, photoplethysmography; ECG, electrocardiography.

These recordings were used to construct three separate surveys (Figure 1), in which the participating physicians were asked to classify the heart rhythm as ‘regular rhythm,' ‘one or more ectopic/missed heartbeats,' ‘atrial flutter,' ‘atrial fibrillation,' ‘unreadable,' or ‘other' via a multiple-choice question formulation. The first survey consisted of PPG data only. For each of the 30 patients, the heart rhythm recording was shown as a PPG waveform (Figure 1A). Subsequently, additional information was added stepwise in the second and third presentation of the PPG rhythm recording. The second presentation consisted of the waveform with the corresponding 60-s tachogram (visualising the duration of peak-to-peak intervals of the waveform) and Poincaré plot (visualises the randomness of the heart rhythm by plotting the peak-to-peak interval relative to the previous peak to peak interval) (Figure 1B). In the third presentation, the FibriCheck® algorithm information was added to the PPG waveform with the plots (Figure 1C). The algorithm information was provided by the proprietary algorithm classifying each measurement as normal (i.e., regular rhythm, green), warning (i.e., possible non-AF irregularity, orange), or urgent (i.e., possible AF, red) and providing the average heart rate during the 60-s measurement. The second and third survey consisted of the single-lead and 12-lead ECG recordings of these 30 patients, respectively.

### Survey Conduction

Physicians with a FibriCheck® dashboard account were invited to participate in the study and were requested to share the invitation with their colleagues. Only upon completion of the first survey, access was provided to the second and third survey presenting single-lead ECG and 12-lead ECG recordings, respectively. There were no time-limits to complete the survey and no feedback was given during or after completing the surveys. Incomplete surveys were excluded from the analysis.

### Statistical Analysis

For the dichotomous comparison, ‘atrial fibrillation' and ‘atrial flutter' answers were regarded as AF, and ‘regular rhythm' and ‘one or more ectopic/missed heartbeats' answers were regarded as non-AF. Recordings labelled as ‘unreadable' were handled as false positive or false negative, as appropriate. If a tracing was labelled ‘other' the physician was requested to specify the diagnosis in a blank text space. These diagnoses were handled as AF or non-AF as appropriate. Two-by-two contingency tables were constructed including all answers to the various PPG representations, single-lead ECG and 12-lead ECG with respect to the reference diagnosis. The sensitivity, specificity, positive predictive value (PPV), negative predictive value (NPV) and accuracy of AF detection were calculated as mean with 95% confidence interval (CI). The PPVs and NPVs were estimated based on an expected AF prevalence of 6% in a population aged >65 years old (7). Additionally, PPVs were calculated for a hypothetical prevalence of 2 and 33%. These calculations were performed using the sensitivities and specificities derived from this study in the formula:


$$\begin{array}{l} PPV=\\ \frac{sensitivity\;x\;prevalence}{\left(sensitivity\;x\;prevalence\right)+\left(\left(1-specificity\right)x\left(1-prevalence\right)\right)\;} \end{array}$$

Sensitivities and specificities were compared with the Obuchowski-Rockette's ANOVA approach with Jackknife covariance estimation and Benjamini-Hochberg correction (8, 9). The sensitivity and specificity, which were modelled separately, were dependent variables in the Obuchowski-Rokkett's ANOVA approach. The technique was an independent variable. The advantage of the Obuchowski-Rokkett's ANOVA approach was that it takes the correlation structure in the data into account via a random effect for reader and a random effect for the test-reader interaction. The results of the various PPG presentations were compared reciprocally. The results of the PPG presentation with plots were compared against single-lead ECG and 12-lead ECG results. The latter were also compared reciprocally. Solely paired data were used in these comparisons. The influence of prior experience on the performance of physicians was analysed using a generalised linear mixed model. Sensitivity and specificity were modelled separately, as dependent variables. As independent variables, experience, technique and the interaction between both were used in the model. The model also accounts for the correlation in the data through a random effect for patient and reader and allows the covariance of both random effects to differ according to the technique. All statistical analyses were 2-sided, and the level of significance was set at 5%. *P*-values were corrected for multiple testing using the Benjamini-Hochberg correction (10). SPSS Statistics 25.0 (IBM, Chicago, IL, USA) was used for descriptive analysis (participant characteristics), RStudio 3.6.3 (RStudio, Boston, USA) was used to perform the Obuchowski-Rockette's ANOVA approach using the R package MRMCaov (9) and the GLIMMIX procedure in SAS 9.4 (SAS, North-Carolina, USA) was used to perform the generalised linear mixed model.

## Results

### Study Population

**Figure 2:**
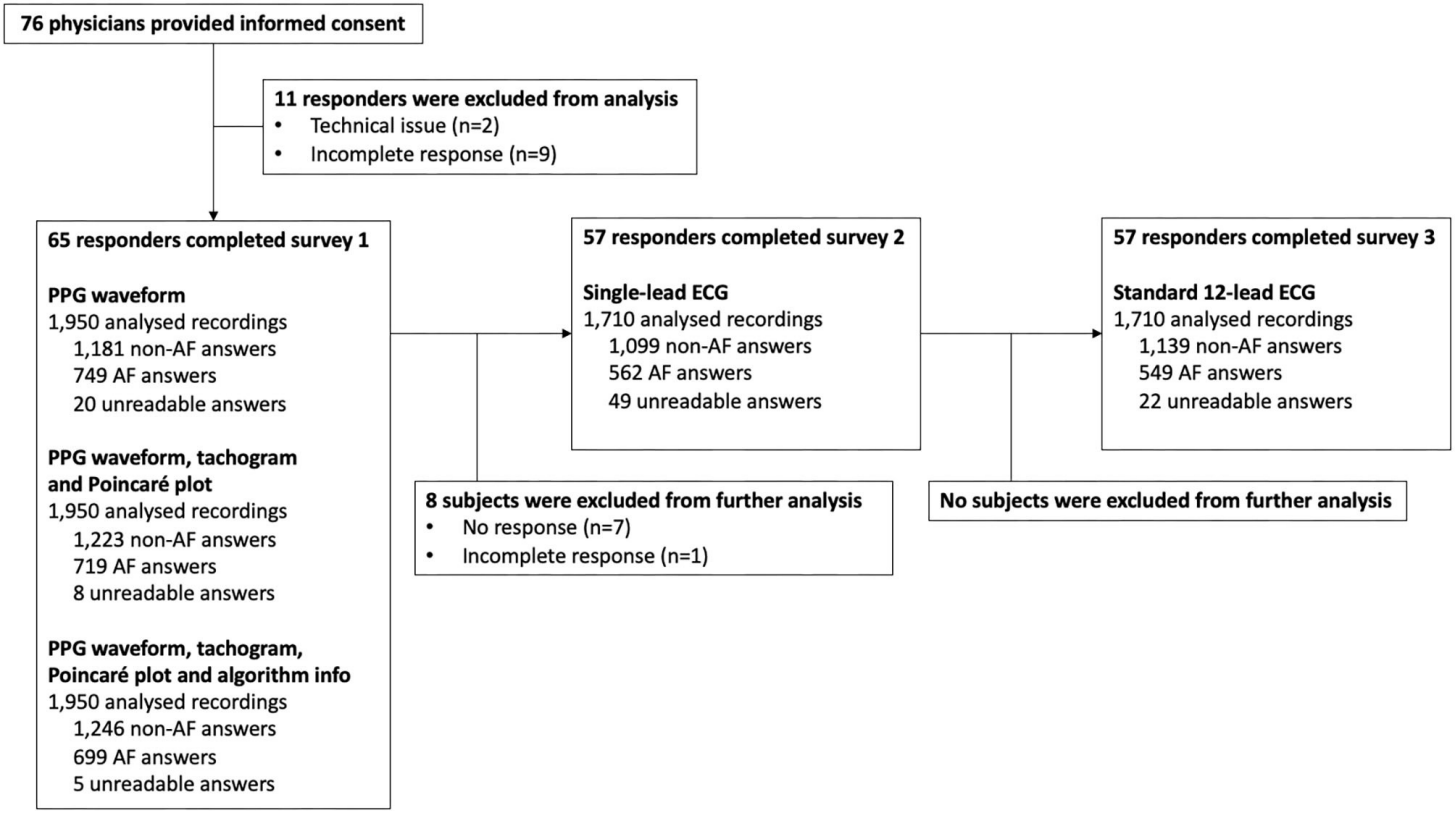
AF, atrial fibrillation; ECG, electrocardiography; PPG, photoplethysmography.

A total of 76 surveys were started of which 11 had to be excluded as a result of technical issues and/or incompleteness (Figure 2). Complete responses, eligible for analysis, were obtained from 30 cardiologists, 26 electrophysiologists, and 9 cardiology fellows (Table 1). Afterwards, the single-lead ECG survey and 12-lead ECG survey were completed by 57 subjects, resulting in a total number of 1,950, 1,710, and 1,710 interpreted recordings, respectively (Figure 2). The participating physicians originated from 33 centres in 9 European countries. 47.7% of them had prior experience with manual PPG analysis.

**Table 1:** Characteristics of the study population.

	**Medical professionals (*n* = 65)**
Age, years (Q1–Q3)	38 (34–47)
Current profession	
Cardiologist	30 (46.2%)
Electrophysiologist	26 (40.0%)
Cardiology fellow	9 (13.8%)
Use PPG in clinical practice	43 (66.2%)
Experience in manual PPG analysis	31 (47.7%)

*PPG, Photoplethysmography; Q1, 25^th^ percentile; Q3, 75^th^ percentile*.

### Performance of Photoplethysmography Analysis

**Figure 3:**
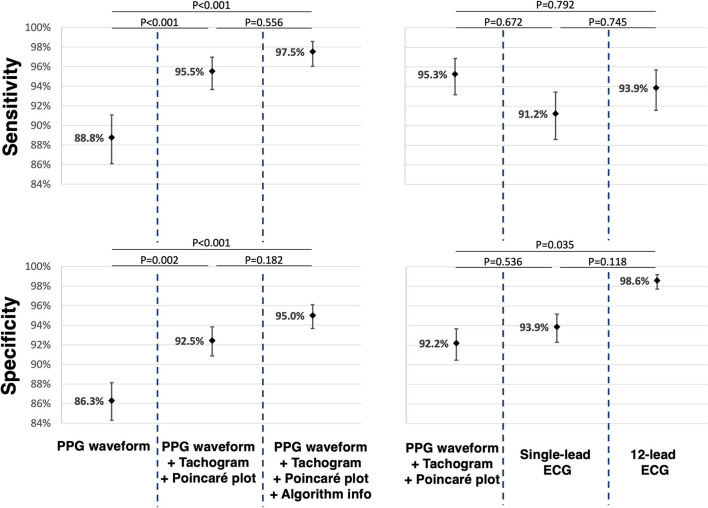
For the comparison of the different PPG presentations reciprocally, paired data of 65 participants were used. For the comparison of PPG, single-lead and 12-lead ECG, paired data of 57 participants were used. The mean sensitivity and mean specificity are displayed as dots with corresponding confidence interval. *P*-values were added for reciprocal comparison. PPG, photoplethysmography; ECG, electrocardiogram.

Data on accuracy is summarised in Table 2. The classification of PPG waveforms alone provided a total of 1,699 (87.1%) correct answers. This yielded a sensitivity of 88.8% (95% CI 86.1–91.1%) and specificity of 86.3% (95% CI 84.3–88.1%) to detect AF. When the corresponding tachogram and Poincaré plot were added in the next step, 182 (9.3%) answers were adjusted; 153 (84.1%) were successfully corrected, whilst 29 (15.9%) were incorrectly adjusted. The sensitivity and specificity to detect AF both increased significantly to 95.5% (95%; CI 93.7–97.0%; *P* < 0.001) and 92.5% (95%; CI 90.9–93.8%; *P* = 0.002), respectively (Figure 3). When the FibriCheck® algorithm output was provided subsequently, 57 (2.9%) answers were adjusted, 51 (89.5%) were successfully corrected, whilst 7 (10.5%) were incorrectly adjusted. The engendered increase in sensitivity to 97.5% (95% CI 96.0–98.6%; *P* = 0.556) and specificity to 95.0% (95% CI 93.7–96.1%; *P* = 0.182) for AF were not statistically significant.

**Table 2:** Accuracy metrics of the qualitative PPG, single-lead ECG, and 12-lead ECG analysis.

	**PPG waveform**	**PPG waveform + Tachogram + Pointcaré plot**	**PPG waveform + Tachogram + Pointcaré plot + Algorithm info**	**Single-lead ECG**	**12-lead ECG**
N physicians	65	65	65	57	57
N qualitatively analysed recordings	1,950	1,950	1,950	1,710	1,710
N unreadable answers (n AF; n non-AF)^*^	20 (1.0%) (14; 6)	8 (0.4%) (8; 0)	5 (0.3%) (5; 0)	49 (2.9%) (21; 28)	22 (1.2%) (20; 2)
Sensitivity, % (95% CI)	88.8 (86.1–91.1)	95.5 (93.7–97.0)	97.5 (96.0–98.6)	91.2 (88.6–93.4)	93.9 (91.6–95.7)
Specificity, % (95% CI)	86.3 (84.3–88.1)	92.5 (90.9–93.8)	95.0 (93.7–96.1)	93.9 (92.3–95.2)	98.6 (97.7–99.2)
PPV^**^, % (95% CI)	29.3 (26.5–32.2)	44.7 (40.1–49.5)	55.5 (49.6–61.2)	48.7 (43.0–54.4)	81.0 (72.4–87.4)
NPV^**^, % (95% CI)	99.2 (99.0–99.3)	99.7 (99.6–99.8)	99.8 (99.7–99.9)	99.4 (99.2–99.5)	99.6 (99.5–99.7)
Accuracy, % (95% CI)	87.1 (85.6–88.6)	93.5 (92.3–94.5)	95.9 (94.9–96.7)	93.0 (91.7–94.2)	97.0 (96.1–97.8)

*PPG, photoplethysmography; ECG, electrocardiography; N, number; AF, atrial fibrillation; CI, confidence interval;*

(*) *Number of readings classified as unreadable by the subjects. n-AF: number of AF recordings classified as unreadable. N non-AF: number of non-AF recordings classified as unreadable;*

(**) *Estimated based on an AF prevalence of 6%*.

The accuracy to detect AF by physicians who reported to be experienced with PPG analysis was not significantly different from the performance of physicians without prior PPG experience for any of the PPG presentations (*P* > 0.37 for sensitivity, *P* > 0.62 for specificity). The average proportion of correct answers in the PPG survey was 93.3% per participating physician (61.1% minimum; 90.0% 1st quartile; 95.6% 3th quartile; 100% maximum).

### Electrocardiography vs. Photoplethysmography Recordings to Detect AF

The mean sensitivity for AF detection based on a single-lead ECG and 12-lead ECG were 91.2% (CI 88.6–93.4%) and 93.8% (95% CI 91.5–95.7%), respectively. There was no significant difference among both, neither when compared to qualitative analysis of the PPG waveforms with plots. The mean specificity for AF detection based on single-lead ECG and 12-lead ECG was 93.9% (95% CI 92.3–95.2%) and 98.6% (95% CI 97.7–99.2%), respectively. The specificity of 12-lead ECG was significantly higher (*P* = 0.035), while for single-lead ECG the specificity was similar (*P* = 0.536) compared to PPG waveforms with corresponding RR-tachograms and Poincaré plots.

### Extrapolating Survey Results to a Hypothetical AF Screening Program

The performance was calculated in a hypothetical population with AF prevalence of 6% (Table 2). The overall accuracy for the raw PPG waveform (87.1%; CI 85.6–88.6) increased numerically when the tachogram and Poincaré plot were provided (93.5%; CI 92.3–94.5) and further increased when the algorithm output was provided (95.9%; CI 94.9–96.7), which was comparable to single-lead ECG (93.0%; 91.7–94.2) but numerically lower than 12-lead ECG (97.0%; 96.1–97.8). A similar trend was observed for the PPV. The raw PPG waveform resulted in the lowest PPV (29.3%; 26.5–32.2), which increased when the plots were provided (44.7%; CI 40.1–49.5%) and when the algorithm output was provided (55.5%; CI 49.6–61.2). This is numerically comparable to single-lead ECG (48.7%; CI 43.0–54.4%), but lower than 12-lead ECG (81.0%; CI 72.4–87.4%). The NPV was above 99.2% for all PPG and ECG outputs.

## Discussion

### Main Findings

This study evaluated the performance of cardiologists and cardiology fellows to differentiate between AF and non-AF rhythms based on PPG, single-lead ECG and 12-lead ECG recordings. The main finding is that physicians can detect AF on a PPG output with equivalent accuracy compared to single-lead ECG in high-quality recordings. To achieve this performance level, a tachogram and Poincaré plot should be provided to facilitate the interpretation of the PPG waveform. These results were consistent in physicians with and without prior PPG experience.

### What Is the Best Way to Present PPG Waveforms to Improve Interpretability?

PPG is new in the clinical toolbox for rhythm monitoring and many physicians are still unfamiliar with the interpretation of PPG outputs. In our study, 53% of the cardiologists and cardiology fellows had no prior experience with manual PPG analysis. This study was designed to evaluate the incremental value of a tachogram and Poincaré plot to the interpretation of a PPG waveform. The performance of physicians to detect AF improved significantly when the PPG waveform was accompanied by the plots. This demonstrates that physicians used the heart rate irregularity presented by these plots as additional information to the PPG morphology and indicates the importance of these plots in PPG analysis.

This presentation of PPG results was also adopted in the PPG dictionary paper by van der Velden et al., and should be used to define and to benchmark the presentation of PPG outputs to interpret PPG signals in clinical practice and for further research (11). Interestingly, the accuracy did not further improve, when the physicians were provided with the FibriCheck® algorithm results in addition to the PPG waveform with plots. It should be noted that the accuracy of the algorithm by itself was not evaluated in this study. The accuracy of the FibriCheck® app has been described in literature with a reported sensitivity of 96% and specificity of 97% to detect AF (12). Potentially, a combined approach of the algorithm classification and manual overreading may result in even better performance and reduce workload, which warrants further study. To compare PPG vs. single-lead ECG, we compared the benchmark presentation (a PPG waveform with plots) against single-lead ECG without providing the algorithm results of either technology. Of note, we did not investigate the accuracy of single-lead ECG combined with the corresponding tachogram and Poincaré plot, which may also have implications for the representation of 12-lead and single-lead ECG recording.

### Photoplethysmography: AF Detection vs. AF Diagnosis

Current guidelines state that when AF is suspected by an automated algorithm, confirmation on an ECG tracing is always required. While the use of a single-lead ECG is a class I recommendation in the ESC 2020 guidelines, the use of PPG alone to establish the diagnosis is not accepted, even when overread by a physician (3). This accords with the general feeling among cardiologists as 83% would diagnose AF based on a single-lead ECG, but only 27% would make the diagnosis based on a PPG output (13). Theoretically, single-lead ECG has some advantages over PPG, such as the ability to evaluate the presence of a p-wave, the QRS width and the QT interval. However, these advantages did not result in a superior performance to detect AF in our study. Particularly if the PPG waveform is combined with the corresponding tachogram and Poincaré plot, physicians could accurately interpret the recordings and detect AF, regardless of prior PPG experience. However, it should be noted that a higher number of different morphologies and other arrythmias could influence these results when applied in clinical practice. Whether the disparity between the general feeling among cardiologist and our study results derive from ignorance toward PPG or superior evaluation of other morphologies and arrythmias with ECG in clinical practice remains to be demonstrated. Currently, PPG is already being used in clinical practice for remote rhythm management in patients who are already diagnosed with AF. By example, in the TeleCheck-AF project on-demand PPG-based rate and rhythm monitoring was used around teleconsultation in 40 centers in Europe during the COVID-19 pandemic (14–16). In this context PPG technology is used to detect, but not diagnose AF. The provided results of this INTERPRET-AF study should trigger further discussion whether every AF episode detected with PPG still needs to be confirmed with ECG documentation to allow the diagnosis of AF in a patient.

### Clinical Implications and Implications for AF Screening

Patients with AF detected with PPG and an automated analysis algorithm should have their PPG output reviewed by a physician. To optimise the accuracy, the output should be presented as a PPG waveform with the corresponding tachogram and Poincaré plot for physician review. This is the new benchmark presentation and the results of this study demonstrate that AF can be detected on this presentation with similar accuracy as on a single-lead ECG trace, but lower specificity then a 12-lead ECG. That is, for measurements of high-quality. As alluded to before, according to current guidelines, ECG confirmation is required to make the diagnosis of AF. However, ECG confirmation may become obsolete as physicians can detect AF on the PPG benchmark presentation with similar accuracy as demonstrated in these results. This is of particular interest in AF screening. The feasibility of PPG based screening for AF has been demonstrated by large scale screening trials that used smartphones to record a 60-s PPG waveform at home (17). In our study, a smartphone was used to generate 60-s of PPG data in the hospital. PPG measurements with a smartphone at home or in the hospital are considered equivalent as the patient is aware when a measurement is made and instant feedback is provided when the measurement is of insufficient quality, allowing the patient to make a new measurement and avoid motion artefacts until the required quality is attained. Similarly, in real-life conditions, the quality assessment is performed by the algorithm before a rhythm classification is performed. In all (>5.5 million) 60-s PPG segments recorded with the FibriCheck application up to August 2021, only 8.2% of the measurements were of insufficient quality (data provided by Qompium NV, Hasselt, Belgium). Measurements of insufficient quality are disregarded and will not be presented to the physician for interpretation. By contrast, screening trials that adopted a smartwatch-based approach can sample more frequent PPG measurements (up to a continuous measurement) (18). However, wearable devices (smartwatches) perform PPG measurements while the patient is unaware and unable to avoid motion artefacts. Hence, this approach does not always result in a high-quality measurement and the classification of insufficient quality measurements is more relevant to PPG deriving wearables. It should be noted, that our study design does not allow extrapolation of these results to measurements of insufficient quality. Both the smartphone- and smartwatch-approach require additional hardware to make a confirmatory ECG documentation of the arrythmia. By contrast, screening programs that use single-lead ECG to screen for AF do not require confirmatory testing to diagnose AF according to current guidelines (19). This study challenges that inequality and suggests that in the absence of other arrythmias, single-lead ECG is not superior to PPG to detect AF when a high-quality PPG waveform is presented with a tachogram and Poincaré plot. Due to the nature of screening, it is likely that confirmational testing of any kind remains indispensable. Hence, this study should open the debate whether confirmatory testing will be possible with PPG as it is with single-lead ECG.

**Figure 4:**
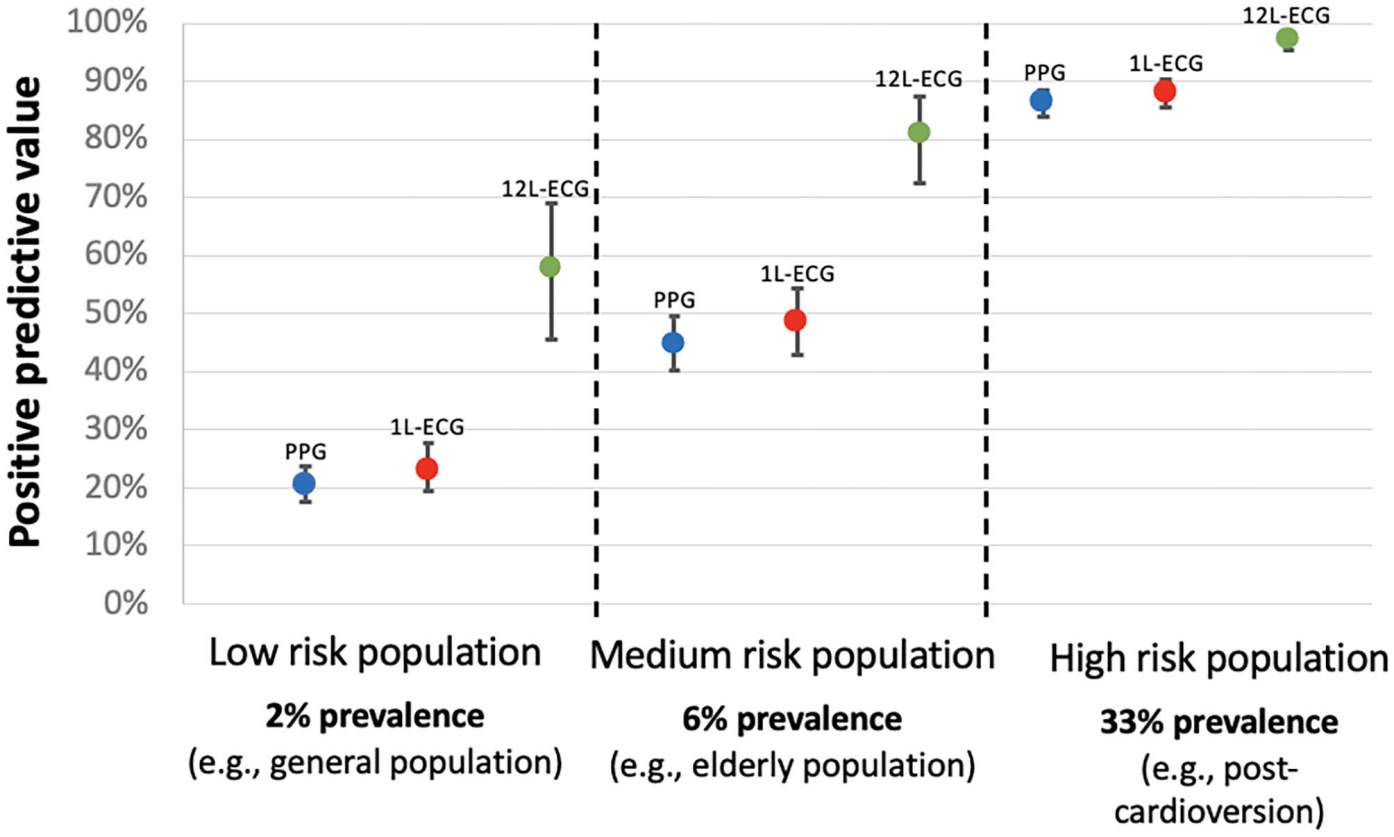
Positive predictive value for AF detection according to the AF prevalence in the targeted population. 1L-ECG, single-lead electrocardiogram; 12L-ECG, 12-lead electrocardiogram; PPG, photoplethysmography (in this context refers to photoplethysmography waveform with tachogram and poincaré plot).

Despite the high sensitivity and specificity of PPG and single-lead ECG for AF detection, the PPV for both drastically declines in populations with a lower AF prevalence. In the setting of AF screening, most studies reported an AF detection rate between 2 and 10%, which is far less than the AF prevalence of 33% in the PPG and ECG dataset presented to the participants in this study (20). To simulate the setting of AF screening in an elderly population, the diagnostic metrics of this study were re-calculated for a hypothetical AF prevalence of 6%. In this simulation, PPG with plots, single-lead ECG and 12-lead ECG all had a very high NPV above 99.2%, but the PPV was moderate for PPG with plots, and for single-lead ECG (44.7 and 48.7%, respectively), suggesting that these detection methods may generate a high number of false positive (FP) diagnoses in AF screening. Figure 4 illustrates how the PPV decreases as the AF prevalence decreases. This highlights the need for confirmational testing after AF detection in a screening program. It has previously been shown that the PPV of handheld single-lead ECG is less than the 12-lead ECG gold standard (between 61.9 and 87.0%), even when interpreted by electrophysiologists in a population with a high AF prevalence (11.9%) (21). However, this is the first study to directly compare PPG and single-lead ECG, suggesting that PPG might be as appropriate as single-lead ECG to confirm AF (if a high-quality PPG output is reviewed by a physician on a waveform with tachogram and Poincaré plot). It will be important to confirm these findings in out-of hospital settings with more morphologies and other arrythmias before enabling smartphones to both detect and confirm AF with PPG.

The purpose of repetitive PPG measurements to confirm AF is to increase the PPV and to lower the false positive rate. There are several reasons why PPG is a suitable tool to fulfil this goal. One, because PPG does not require additional hardware, PPG can easily be used with repetitive measurements which by itself is a strong mechanism to improve specificity and reduce the number of false positives. Two, combining AF detection algorithms and manual interpretation can decrease the false positive rate in PPG and single-lead ECG screening strategies (17). In this study, the algorithm output did not significantly improve the accuracy, however the algorithm did guide physicians to correct their response in 51 cases. Three, advances in deep learning algorithms will likely continue to improve the robustness of PPG algorithms and ECG algorithms resulting in increased an PPV and fewer false positives (5).

Further larger studies also focusing on the real-life performance of physicians to interpret PPG waveforms and the non-inferiority of treatment of ECG- vs. PPG-detected AF on AF outcomes are required to clarify the predictive values in population screening. Both PPG and single-lead ECG based screening with both algorithms as well as physician interpretation should be validated in real-world setting with appropriate AF prevalence and where AF detection is complicated by other cardiac arrythmias and artefacts that were not included in the current validation studies.

## Limitations

We acknowledge several limitations to this survey-based study approach. One, the number of responders, participating in the study, was limited. Despite, statistical power was attained as a result of the high number of questions per subject. Two, there may be a selection bias, as the invited physicians were mainly physicians with an existing FibriCheck® dashboard. However, according to our survey results, almost half of the responders did not have prior PPG experience. Three, the tracings chosen for PPG and ECG represent an artificial population and may limit extrapolation to real-world use. Therefore, the prevalence was adjusted in the PPV and NPV calculation. Four, this study only evaluated the differentiation between AF and non-AF rhythms as a dichotomous classification and did not take a broader differential diagnosis into account. Five, only high-quality measurements performed in the cardiology department were selected in the survey, limiting the extrapolation to wearable devices (smartwatches) performing PPG measurements while the patient is unaware and unable to avoid motion artefacts. Six, only measurements of 30 patients were included in the survey. These findings should be confirmed in a larger population with more morphologies and arrythmias.

## Conclusion

Physicians can detect AF on a high-quality PPG output with equivalent accuracy compared to single-lead ECG, even without prior PPG training. To achieve this performance level, a tachogram and Poincaré plot should be provided to facilitate the interpretation of the PPG waveform. Such rhythm interpretation is even as sensitive as 12-lead ECG to detect AF. However, it remains that 12-lead ECG is more specific, and thus results in a higher positive predictive value and fewer false positives. This is the first paper to describe a method of PPG presentation that should be used for future benchmarking studies. Subsequent studies should be conducted in real world settings to confirm or disprove the findings suggested by this study, that high-quality PPG recordings might be as suitable as single-lead ECG to diagnose AF.

## Data Availability Statement

The raw data supporting the conclusions of this article will be made available by the authors, without undue reservation.

## Ethics Statement

The studies involving human participants were reviewed and approved by Comité Medische Ethiek, Ziekenhuis Oost-Limburg. The patients/participants provided their written informed consent to participate in this study.

## Author Contributions

SE and TP were involved in data collection. SE, HG, LP, PV, and PH were involved in the statistical analysis. HG wrote the initial manuscript. All authors read, reviewed, and edited the manuscript in the subsequent revision rounds.

## Conflict of Interest

SE and TP are employed by Qompium NV. DD received speaker honoraria and/or travel grants from Abbott, Astra-Zeneca, Bayer, Biotronic, Boehringer-Ingelheim, Boston Scientific, Medtronic, Pfizer and Zoll. HH did receive personal fees from Biotronik and Pfizer-BMS. He received unconditional research grants through the University of Antwerp and/or the University of Hasselt from Bayer, Boehringer-Ingelheim, Bracco Imaging Europe, Abbott, Medtronic, Biotronik, Daicchi-Sankyo, Pfizer- BMS, and Boston-Scientific, all outside the scope of this work. MM received speaker honoraria and/or travel grants from Biosense Webster, Abbott, Biotronik, Zoll, Boston Scientific, Daiichi Sankyo, Bayer, Pfizer, Amomed as well as research grants from Biosense Webster, none of which are relevant to the manuscript. PV holds stock in Qompium NV. The remaining authors declare that the research was conducted in the absence of any commercial or financial relationships that could be construed as a potential conflict of interest.

## Publisher's Note

All claims expressed in this article are solely those of the authors and do not necessarily represent those of their affiliated organizations, or those of the publisher, the editors and the reviewers. Any product that may be evaluated in this article, or claim that may be made by its manufacturer, is not guaranteed or endorsed by the publisher.
