# Supplementary material for: Accuracy of Physicians Interpreting Photoplethysmography and Electrocardiography Tracings to Detect Atrial Fibrillation: INTERPRET-AF
Source: Front Cardiovasc Med. 2021 Sep 20;8:734737. doi: 10.3389/fcvm.2021.734737 (PMC8488290; doi:10.3389/fcvm.2021.734737)

INTERPRET-AF_12-lead_ECG

* 2. How would you classify the following measurement?

- Regular rhythm
- One or more ectopic/missed heartbeats
- Atrial flutter
- Atrial fibrillation
- Unreadable
- Other (please specify)

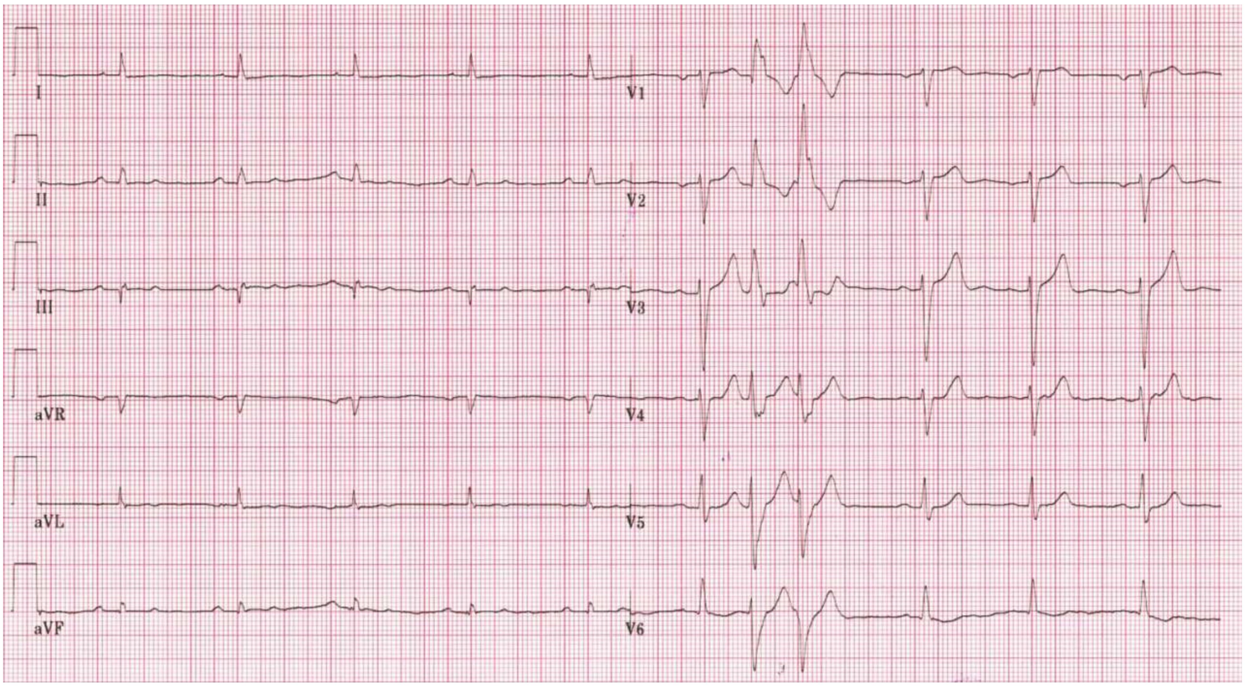

INTERPRET-AF_12-lead_ECG

* 3. How would you classify this measurement?

- Regular rhythm
- One or more ectopic/missed heartbeats
- Atrial flutter
- Atrial fibrillation
- Unreadable
- Other (please specify)

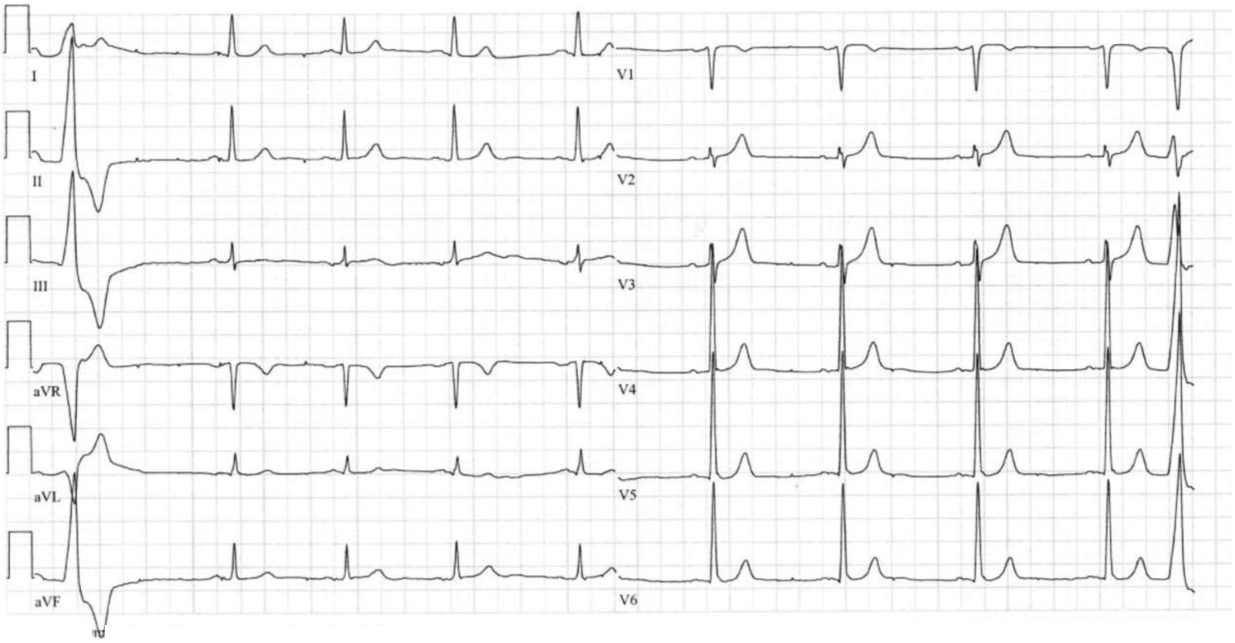

INTERPRET-AF_12-lead_ECG

* 4. How would you classify the following measurement?

- Regular rhythm
- One or more ectopic/missed heartbeats
- Atrial flutter
- Atrial fibrillation
- Unreadable
- Other (please specify)

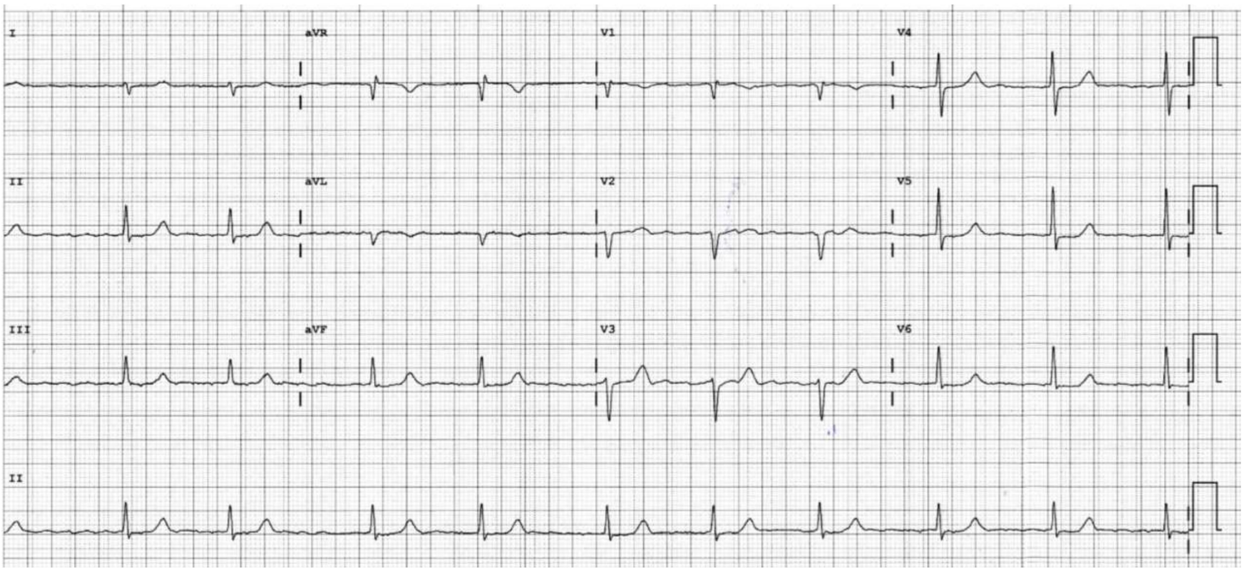

INTERPRET-AF_12-lead_ECG

* 5. How would you classify the following measurement?

- Regular rhythm
- One or more ectopic/missed heartbeats
- Atrial flutter
- Atrial fibrillation
- Unreadable
- Other (please specify)

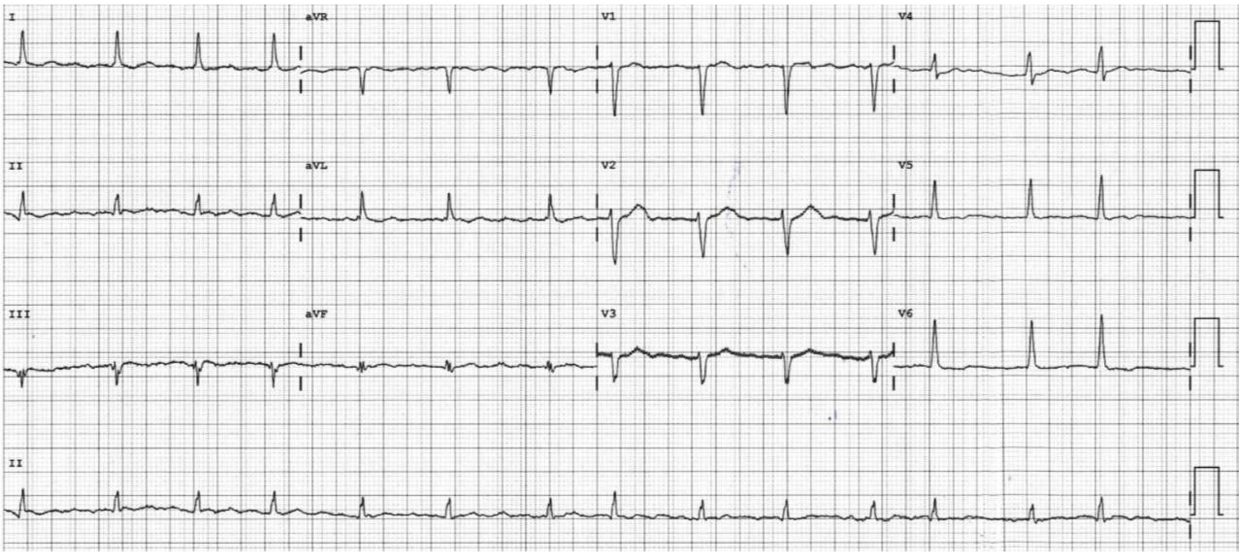

INTERPRET-AF_12-lead_ECG

* 6. How would you classify the following measurement?

- Regular rhythm
- One or more ectopic/missed heartbeats
- Atrial flutter
- Atrial fibrillation
- Unreadable
- Other (please specify)

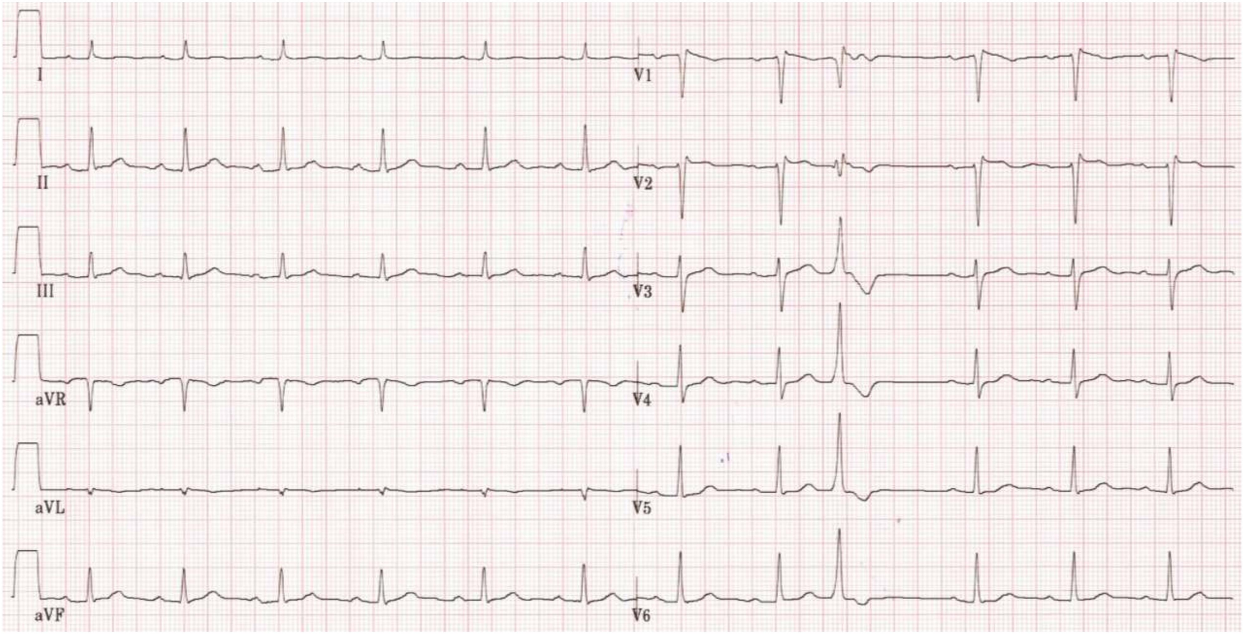

INTERPRET-AF_12-lead_ECG

* 7. How would you classify the following measurement?

- Regular rhythm
- One or more ectopic/missed heartbeats
- Atrial flutter
- Atrial fibrillation
- Unreadable
- Other (please specify)

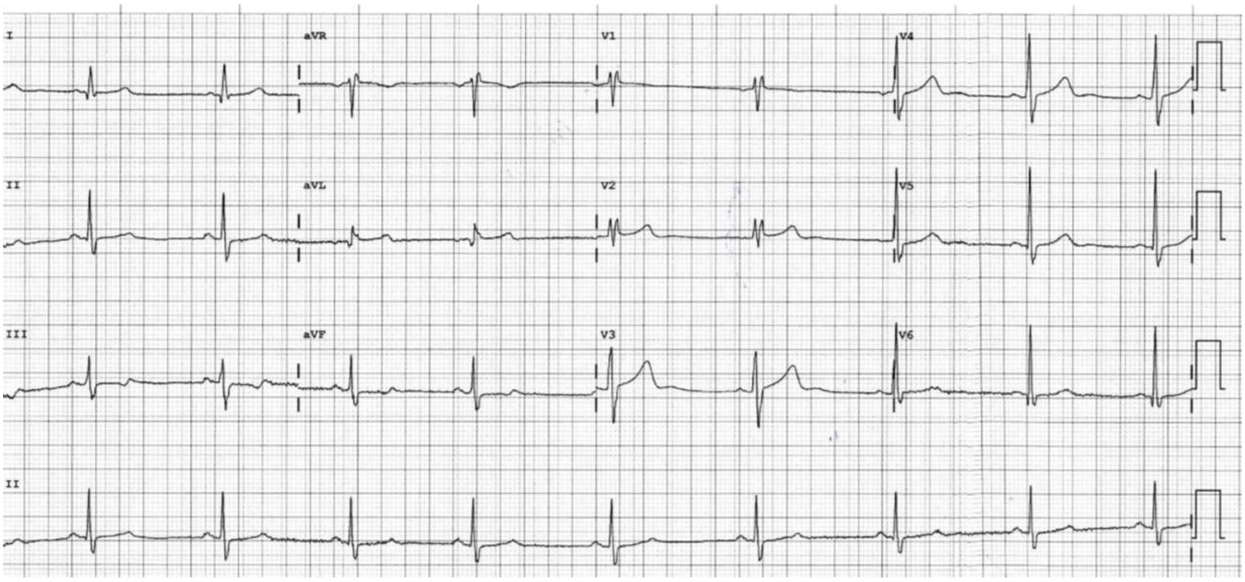

INTERPRET-AF_12-lead_ECG

* 8. How would you classify the following measurement?

- ◯ Regular rhythm
- ◯ One or more ectopic/missed heartbeats
- ◯ Atrial flutter
- ◯ Atrial fibrillation
- ◯ Unreadable
- ◯ Other (please specify)

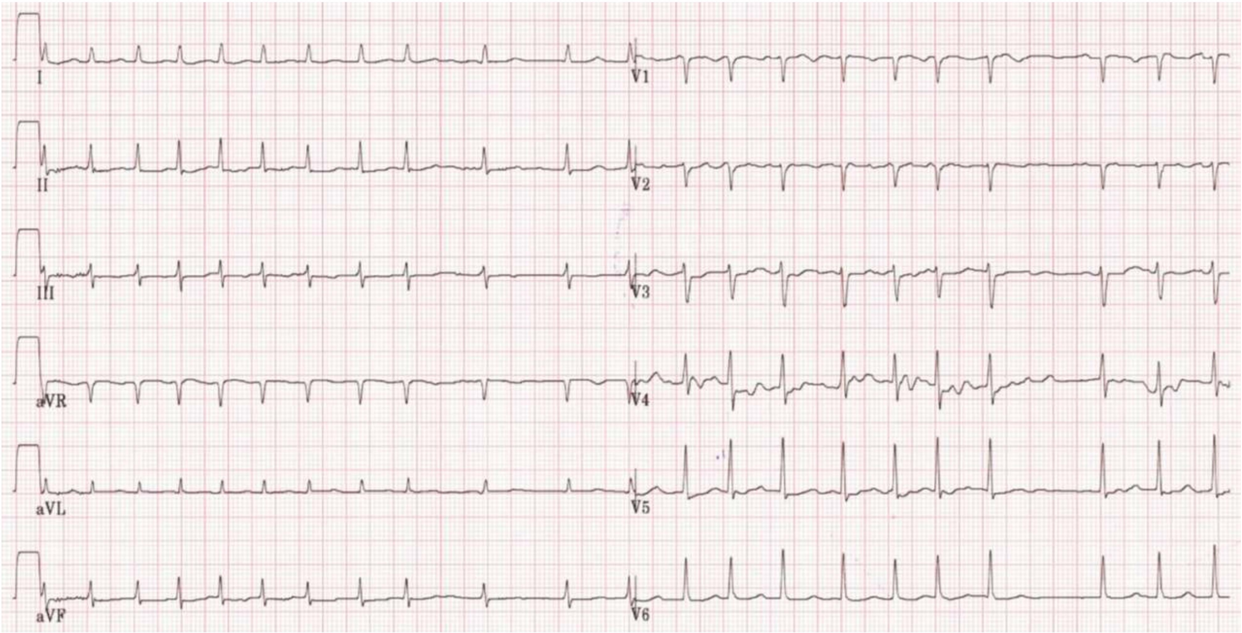

INTERPRET-AF_12-lead_ECG

* 9. How would you classify the following measurement?

- ◯ Regular rhythm
- ◯ One or more ectopic/missed heartbeats
- ◯ Atrial flutter
- ◯ Atrial fibrillation
- ◯ Unreadable
- ◯ Other (please specify)

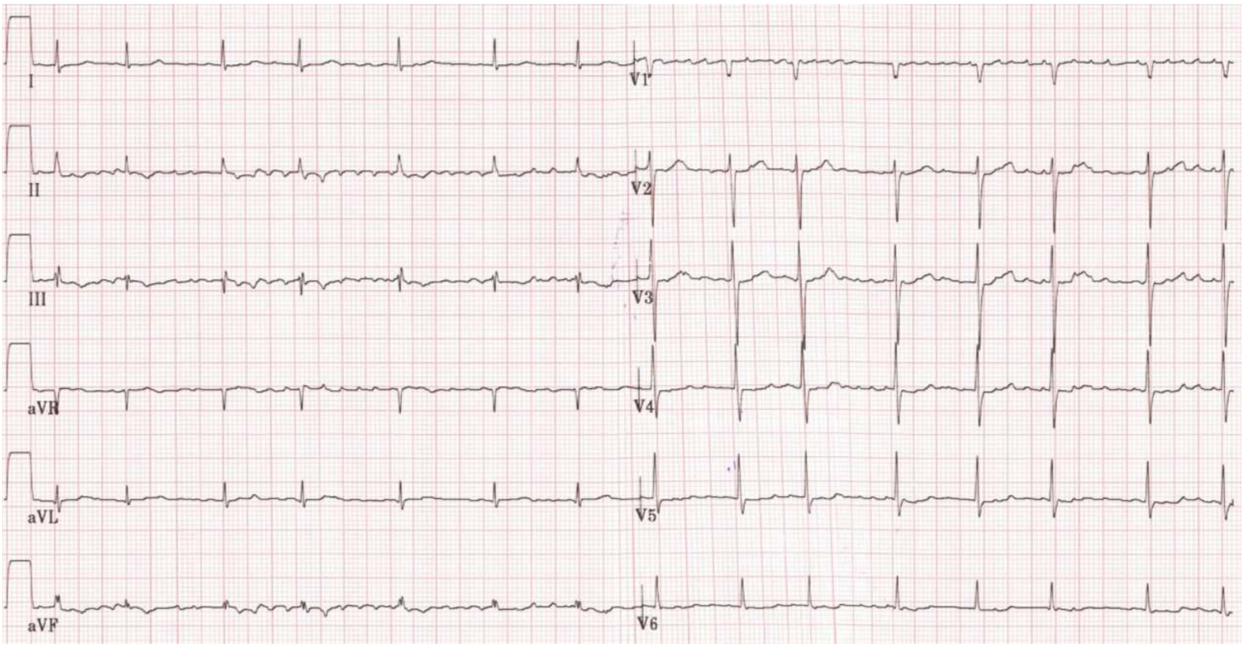

INTERPRET-AF_12-lead_ECG

* 10. How would you classify the following measurement?

- ◯ Regular rhythm
- ◯ One or more ectopic/missed heartbeats
- ◯ Atrial flutter
- ◯ Atrial fibrillation
- ◯ Unreadable
- ◯ Other (please specify)

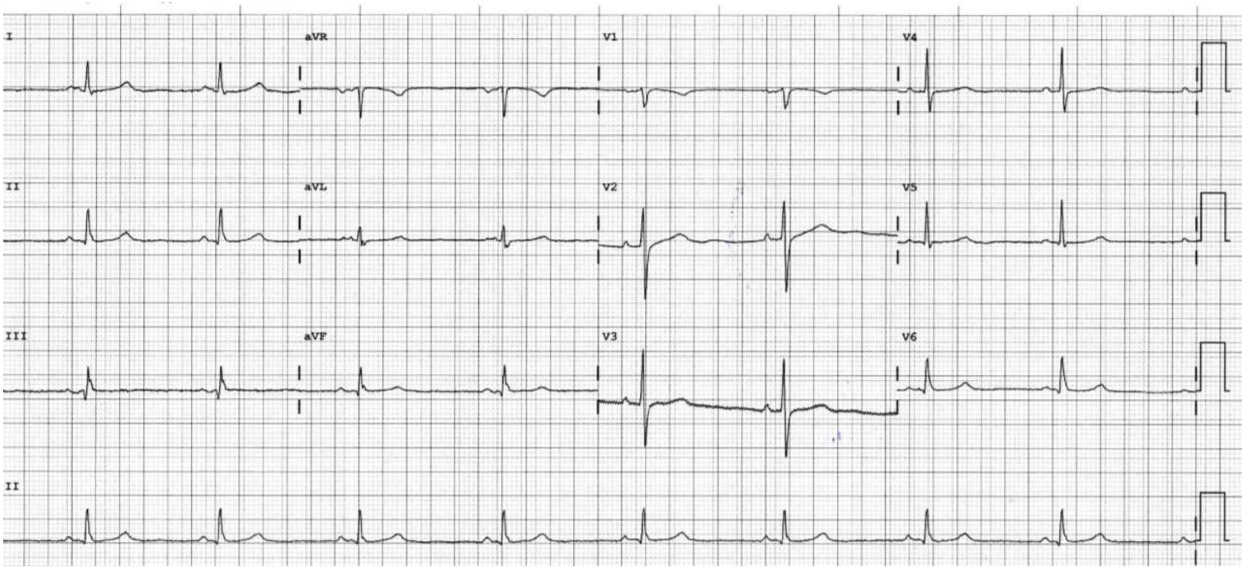

INTERPRET-AF_12-lead_ECG

* 11. How would you classify the following measurement?

- Regular rhythm
- One or more ectopic/missed heartbeats
- Atrial flutter
- Atrial fibrillation
- Unreadable
- Other (please specify)

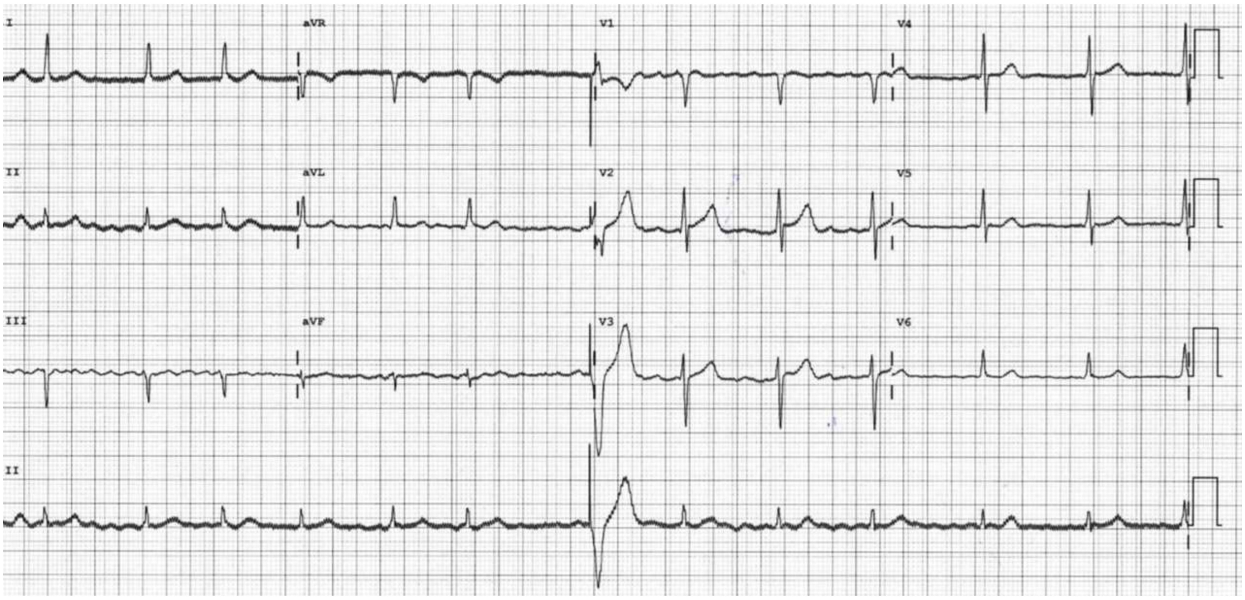

INTERPRET-AF_12-lead_ECG

* 12. How would you classify the following measurement?

- Regular rhythm
- One or more ectopic/missed heartbeats
- Atrial flutter
- Atrial fibrillation
- Unreadable
- Other (please specify)

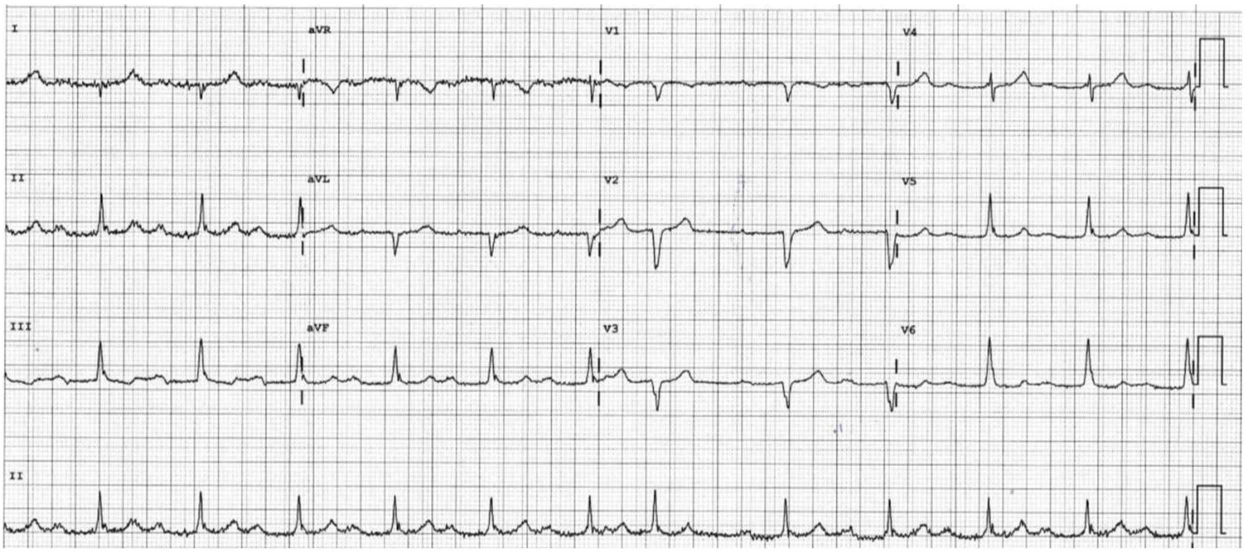

INTERPRET-AF_12-lead_ECG

* 13. How would you classify the following measurement?

- Regular rhythm
- One or more ectopic/missed heartbeats
- Atrial flutter
- Atrial fibrillation
- Unreadable
- Other (please specify)

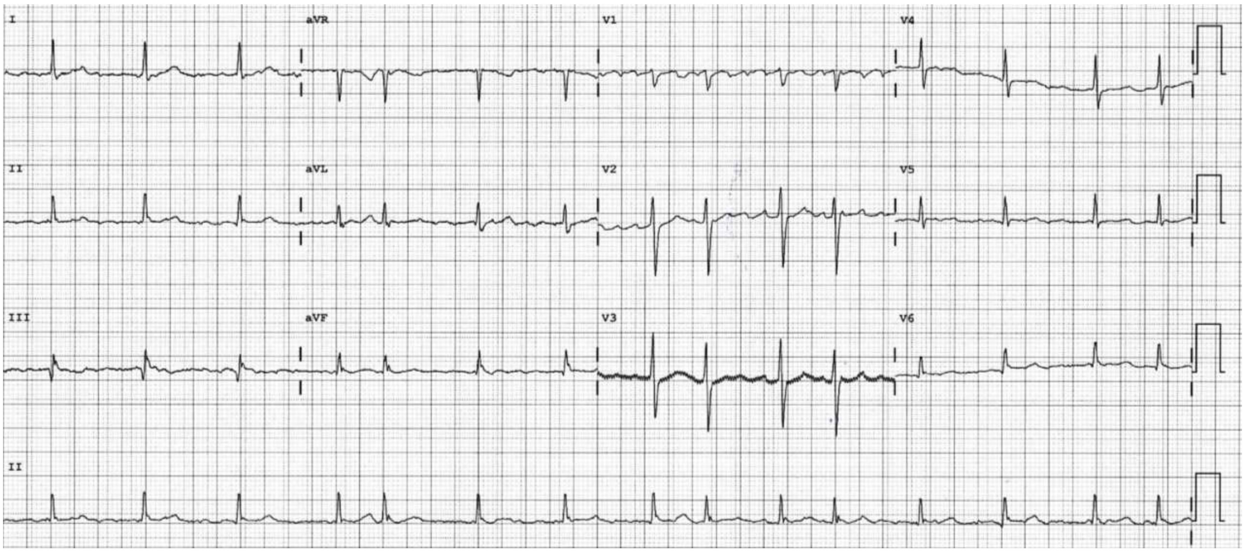

INTERPRET-AF_12-lead_ECG

* 14. How would you classify the following measurement?

- ◯ Regular rhythm
- ◯ One or more ectopic/missed heartbeats
- ◯ Atrial flutter
- ◯ Atrial fibrillation
- ◯ Unreadable
- ◯ Other (please specify)

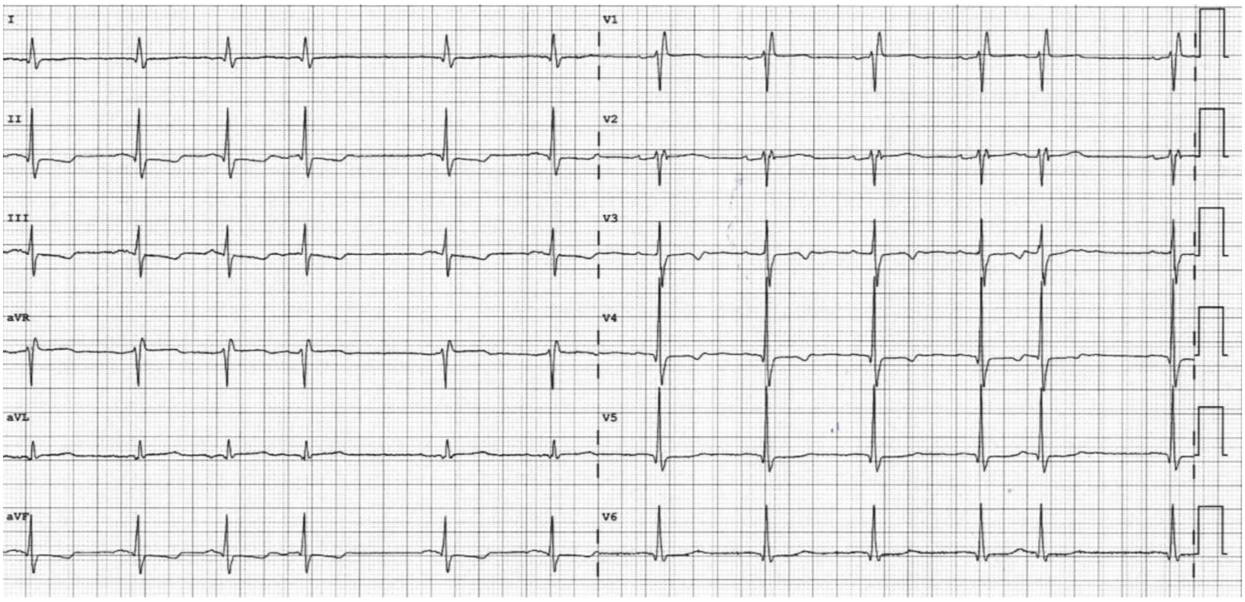

INTERPRET-AF_12-lead_ECG

* 15. How would you classify the following measurement?

- ◯ Regular rhythm
- ◯ One or more ectopic/missed heartbeats
- ◯ Atrial flutter
- ◯ Atrial fibrillation
- ◯ Unreadable
- ◯ Other (please specify)

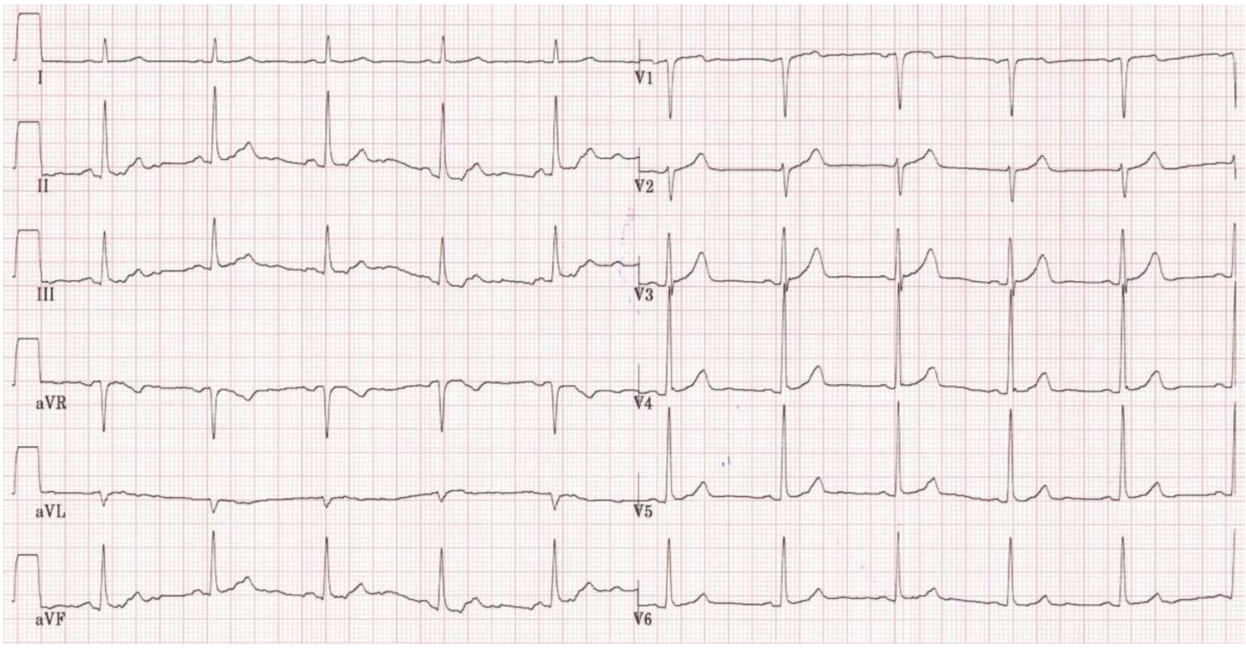

INTERPRET-AF_12-lead_ECG

* 16. How would you classify the following measurement?

- Regular rhythm
- One or more ectopic/missed heartbeats
- Atrial flutter
- Atrial fibrillation
- Unreadable
- Other (please specify)

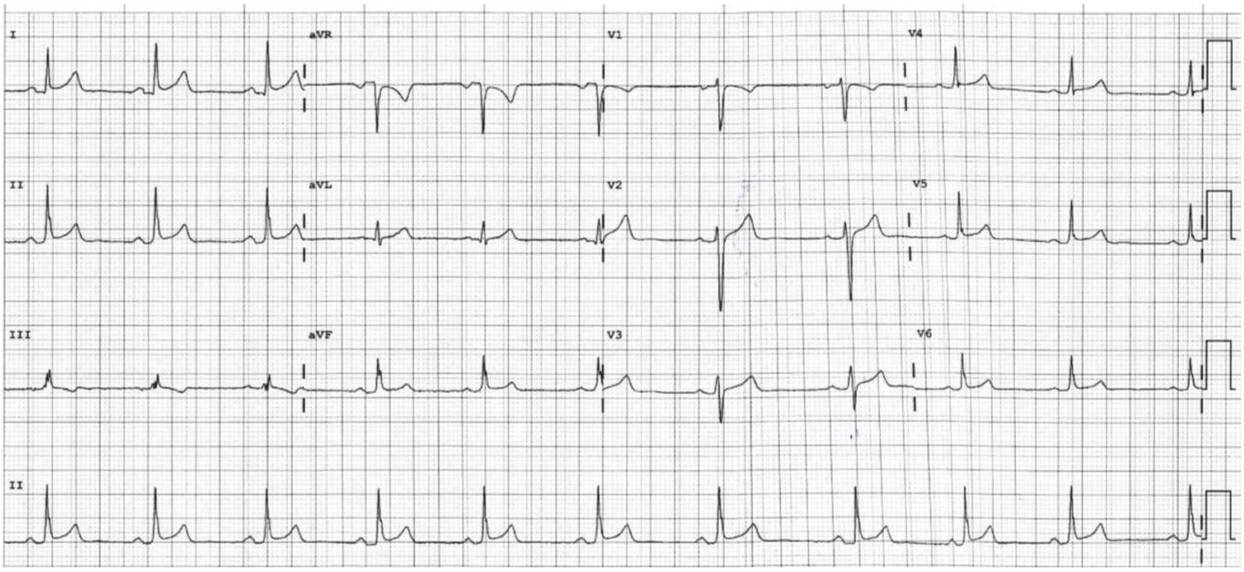

INTERPRET-AF_12-lead_ECG

* 17. How would you classify the following measurement?

- ◯ Regular rhythm
- ◯ One or more ectopic/missed heartbeats
- ◯ Atrial flutter
- ◯ Atrial fibrillation
- ◯ Unreadable
- ◯ Other (please specify)

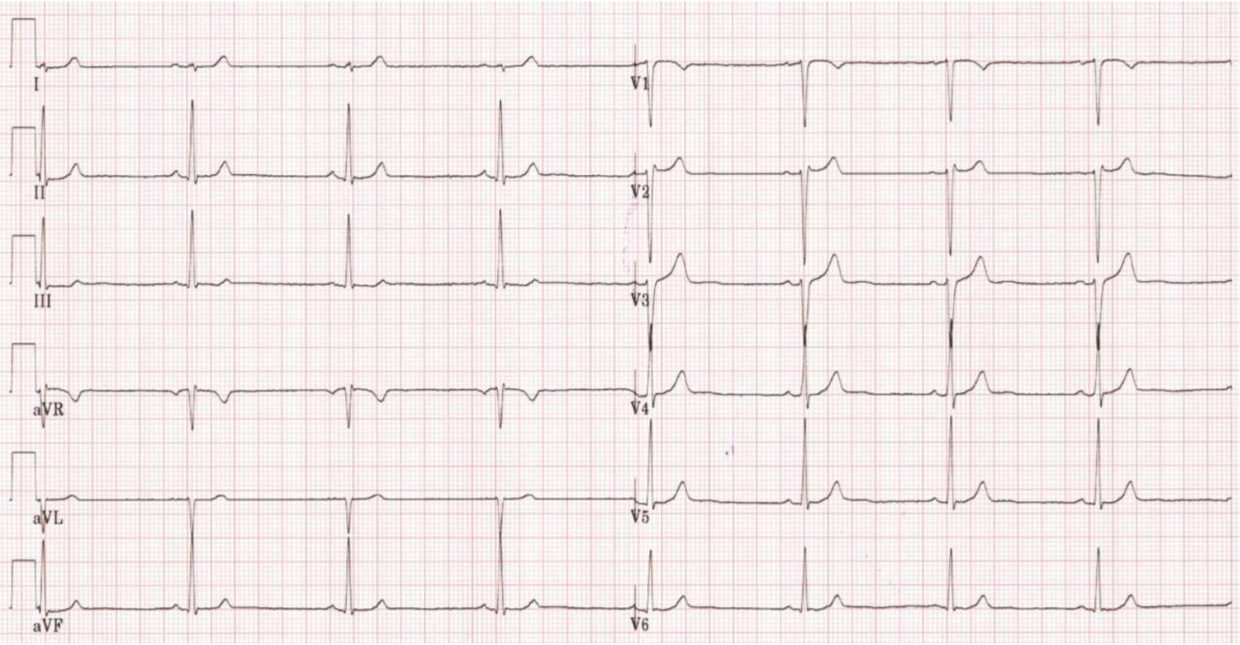

INTERPRET-AF_12-lead_ECG

* 18. How would you classify the following measurement?

- Regular rhythm
- One or more ectpic/missed heartbeats
- Atrial flutter
- Atrial fibrillation
- Unreadable
- Other (please specify)

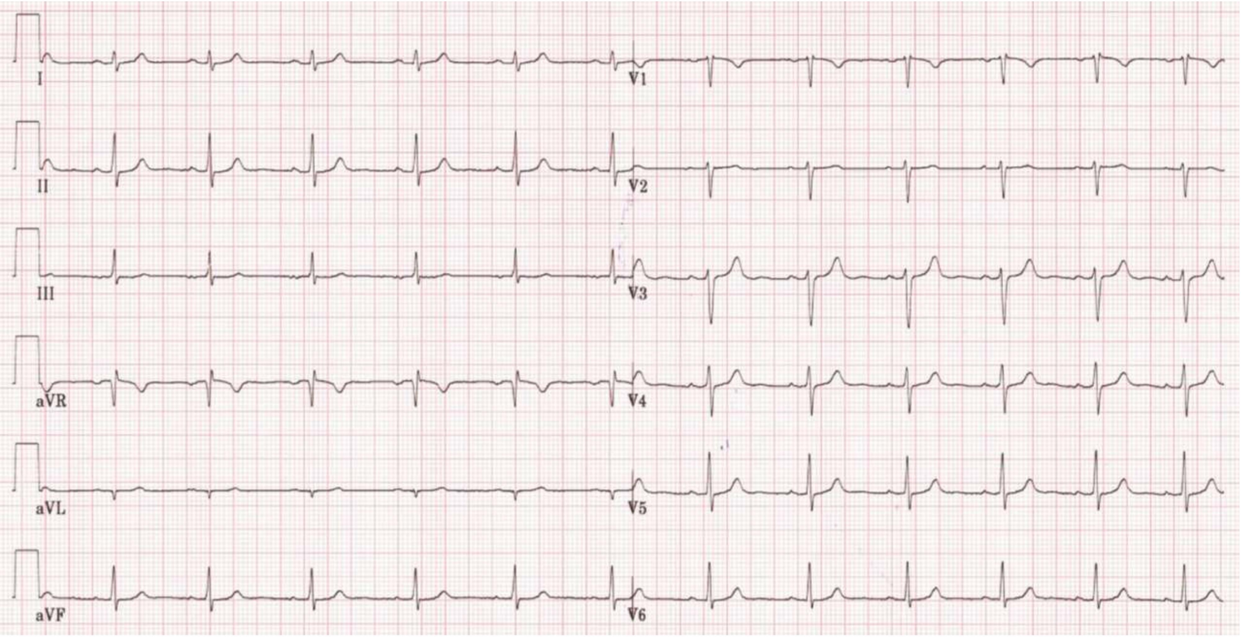

INTERPRET-AF_12-lead_ECG

* 19. How would you classify the following measurement?

- Regular rhythm
- One or more ectopic/missed heartbeats
- Atrial flutter
- Atrial fibrillation
- Unreadable
- Other (please specify)

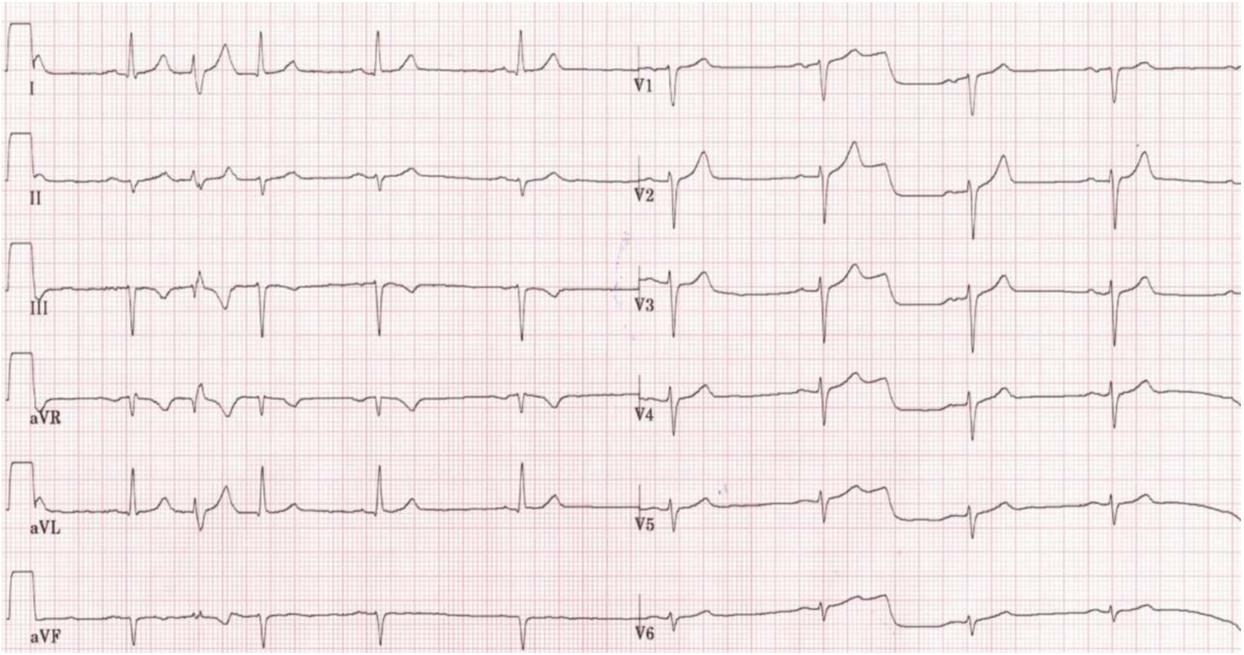

INTERPRET-AF_12-lead_ECG

* 20. How would you classify the following measurement?

- ◯ Regular rhythm
- ◯ One or more ectopic/missed heartbeats
- ◯ Atrial flutter
- ◯ Atrial fibrillation
- ◯ Unreadable
- ◯ Other (please specify)

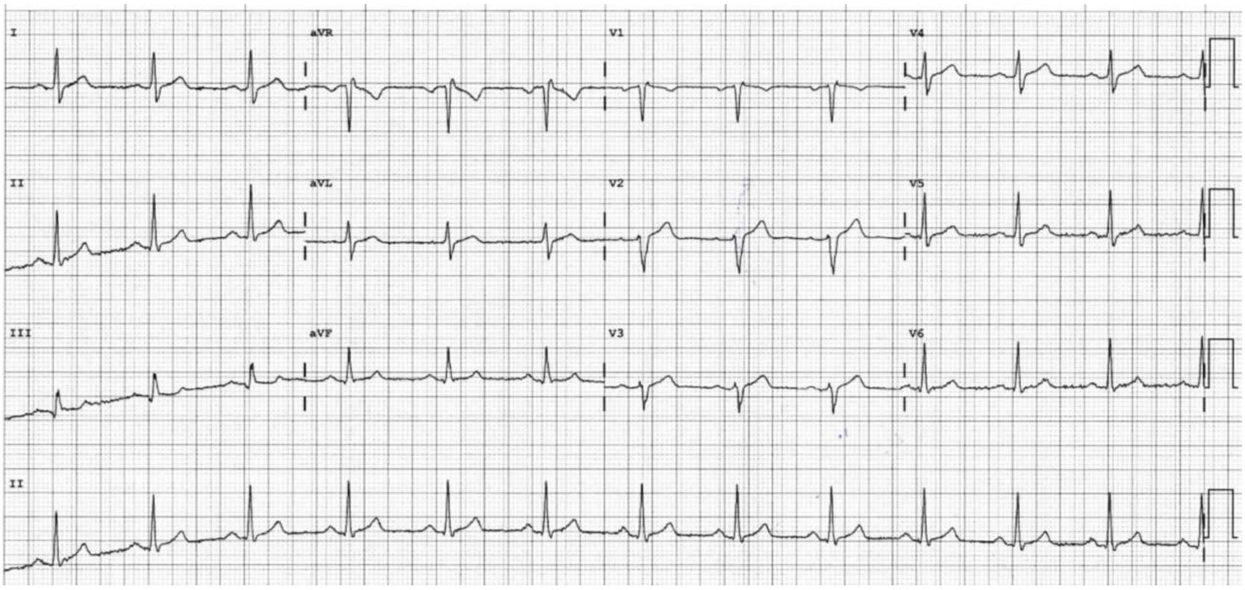

INTERPRET-AF_12-lead_ECG

* 21. How would you classify the following measurement?

- Regular rhythm
- One or more ectopic/missed heartbeats
- Atrial flutter
- Atrial fibrillation
- Unreadable
- Other (please specify)

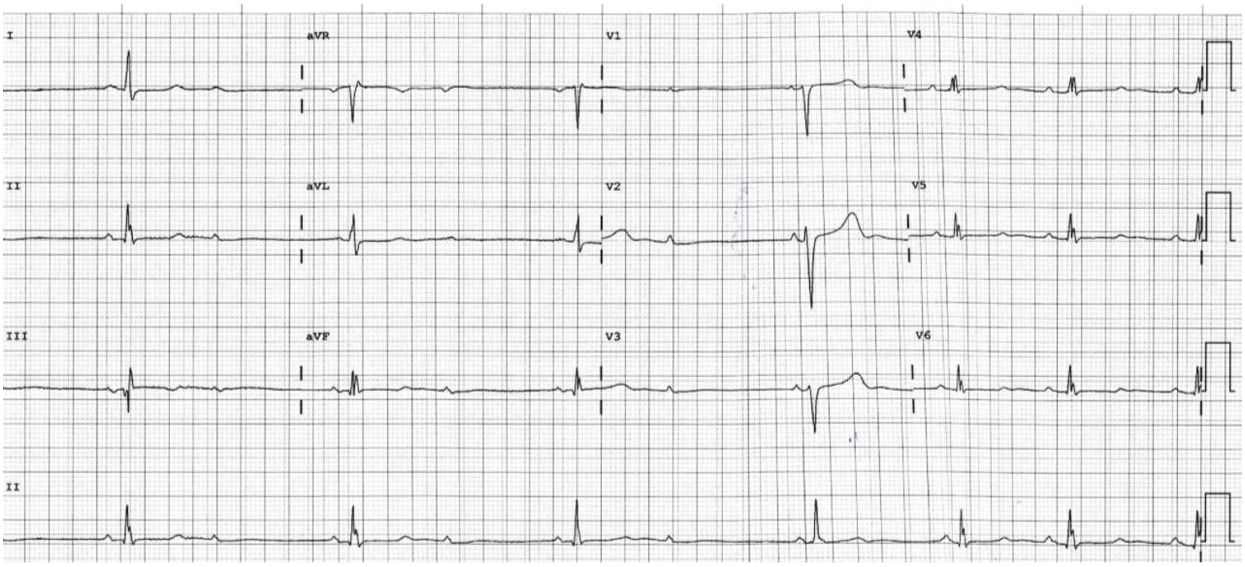

INTERPRET-AF_12-lead_ECG

* 22. How would you classify the following measurement?

- Regular rhythm
- One or more ectopic/missed heartbeats
- Atrial flutter
- Atrial fibrillation
- Unreadable
- Other (please specify)

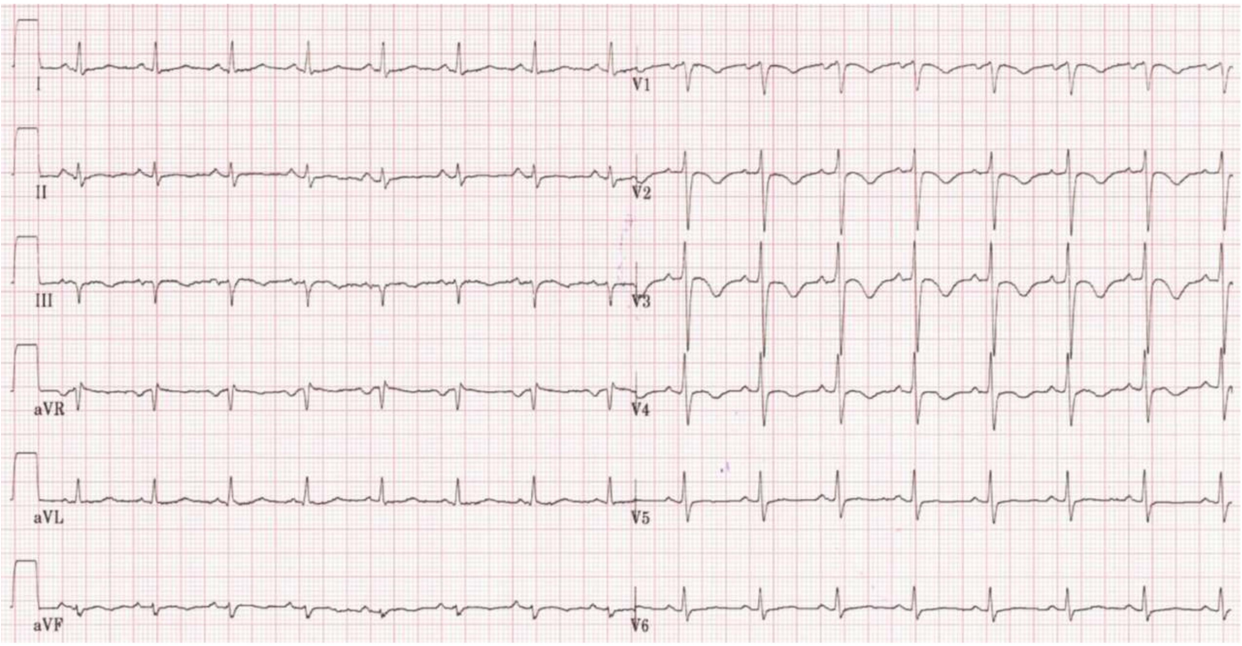

INTERPRET-AF_12-lead_ECG

* 23. How would you classify the following measurement?

- ◯ Regular rhythm
- ◯ One or more ectopic/missed heartbeats
- ◯ Atrial flutter
- ◯ Atrial fibrillation
- ◯ Unreadable
- ◯ Other (please specify)

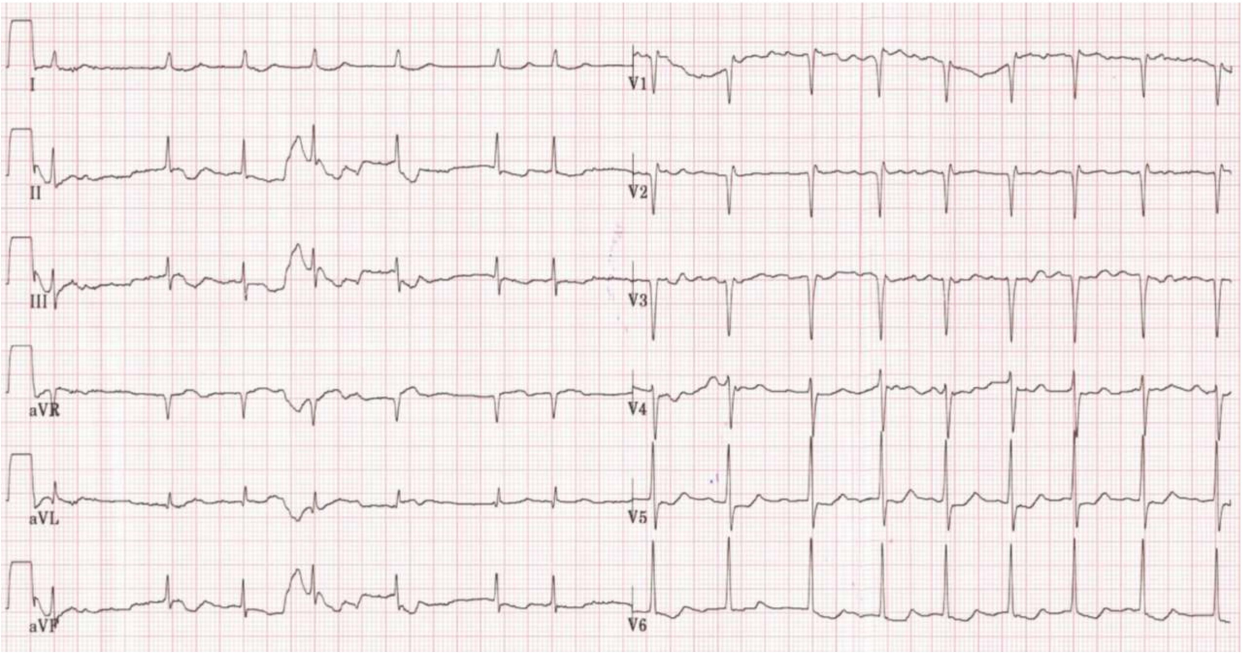

INTERPRET-AF_12-lead_ECG

* 24. How would you classify the following measurement?

- ◯ Regular rhythm
- ◯ One or more ectopic/missed heartbeats
- ◯ Atrial flutter
- ◯ Atrial fibrillation
- ◯ Unreadable
- ◯ Other (please specify)

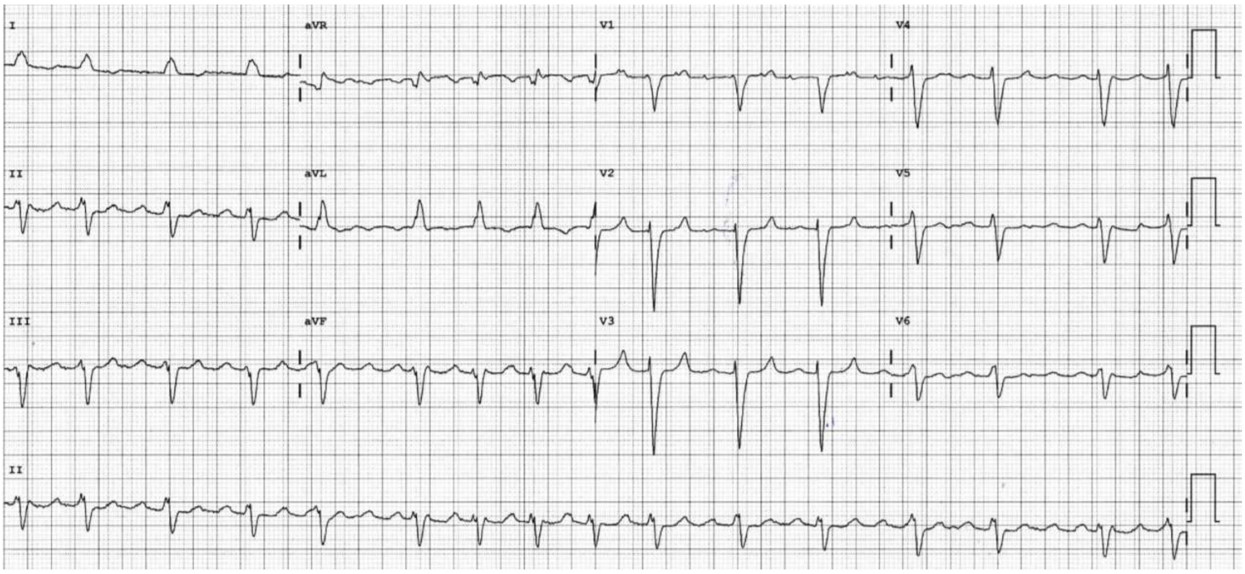

INTERPRET-AF_12-lead_ECG

* 25. How would you classify the following measurement?

- ◯ Regular rhythm
- ◯ One or more ectopic/missed heartbeats
- ◯ Atrial flutter
- ◯ Atrial fibrillation
- ◯ Unreadable
- ◯ Other (please specify)

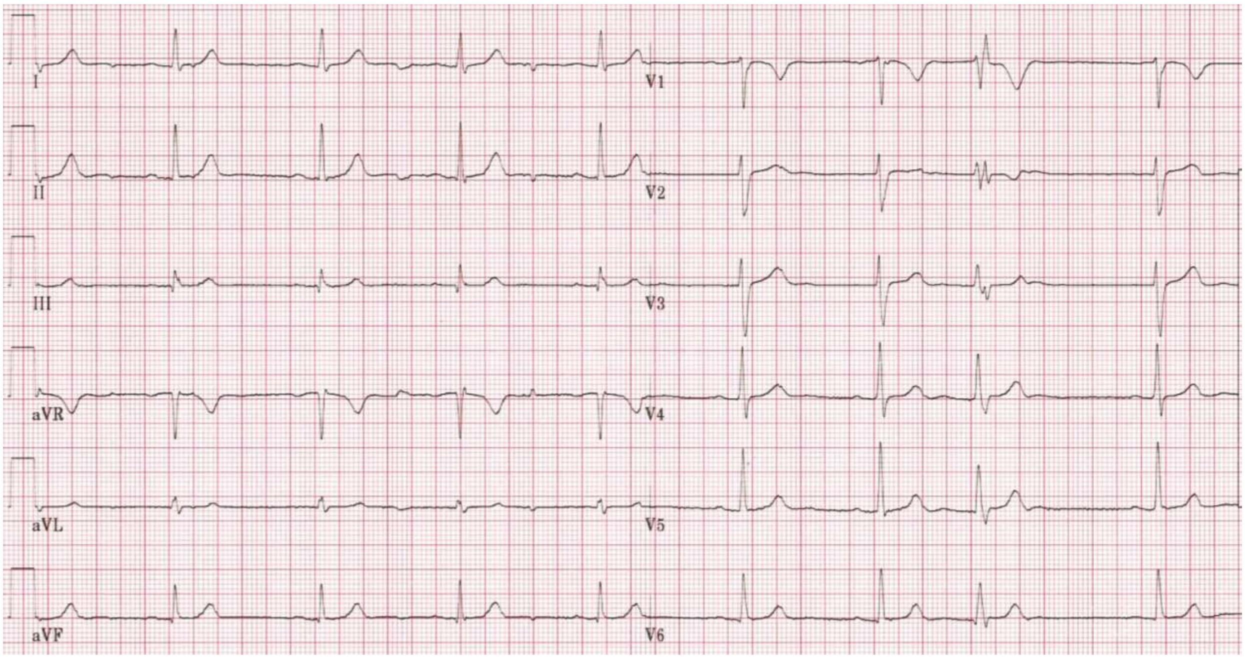

INTERPRET-AF_12-lead_ECG

* 26. How would you classify the following measurement?

- ◯ Regular rhythm
- ◯ One or more ectopic/missed heartbeats
- ◯ Atrial flutter
- ◯ Atrial fibrillation
- ◯ Unreadable
- ◯ Other (please specify)

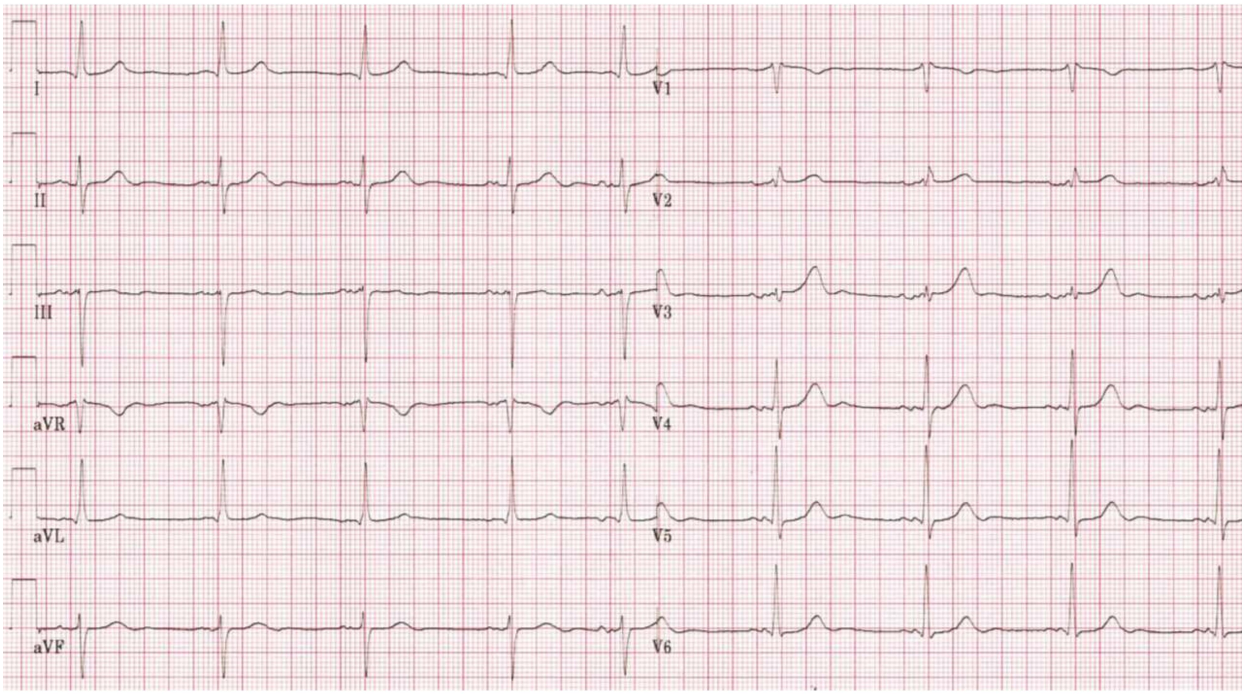

INTERPRET-AF_12-lead_ECG

* 27. How would you classify the following measurement?

- ◯ Regular rhythm
- ◯ One or more ectopic/missed heartbeats
- ◯ Atrial flutter
- ◯ Atrial fibrillation
- ◯ Unreadable
- ◯ Other (please specify)

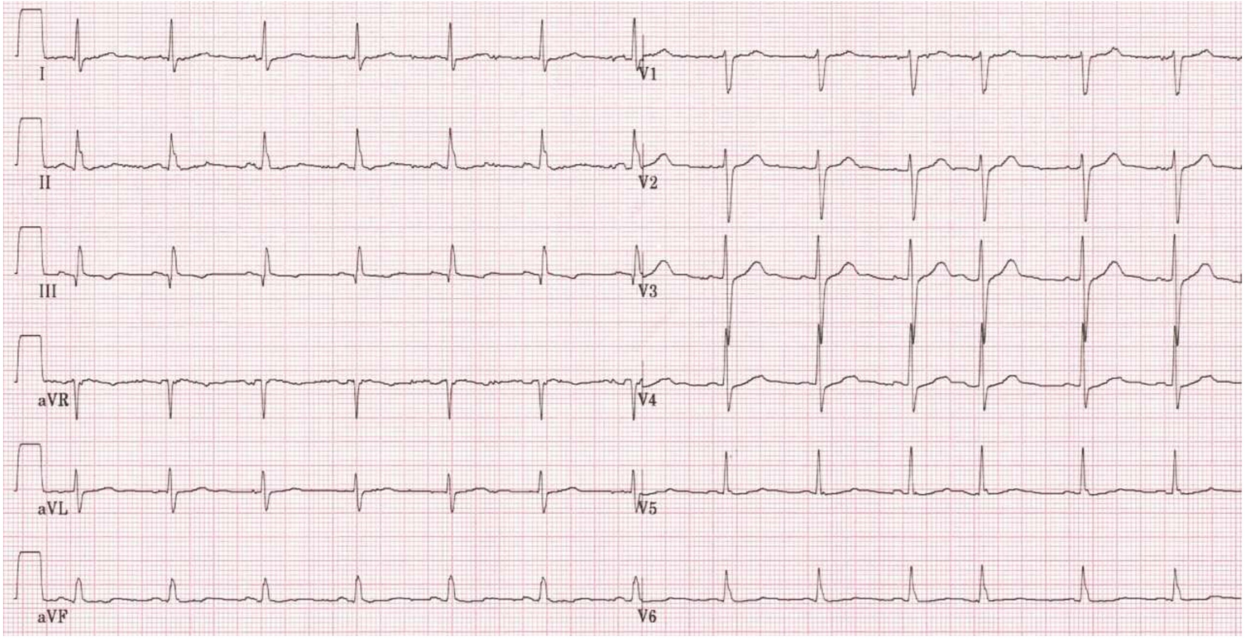

INTERPRET-AF_12-lead_ECG

* 28. How would you classify the following measurement?

- ◯ Regular rhythm
- ◯ One or more ectopic/missed heartbeats
- ◯ Atrial flutter
- ◯ Atrial fibrillation
- ◯ Unreadable
- ◯ Other (please specify)

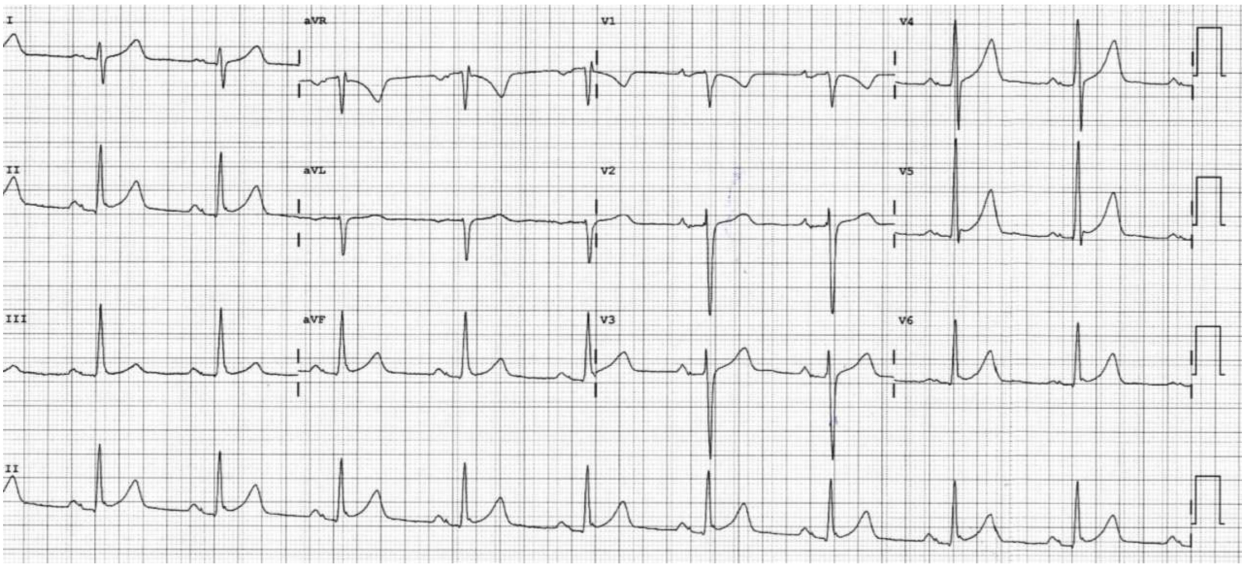

INTERPRET-AF_12-lead_ECG

* 29. How would you classify the following measurement?

- Regular rhythm
- One or more ectopic/missed heartbeats
- Atrial flutter
- Atrial fibrillation
- Unreadable
- Other (please specify)

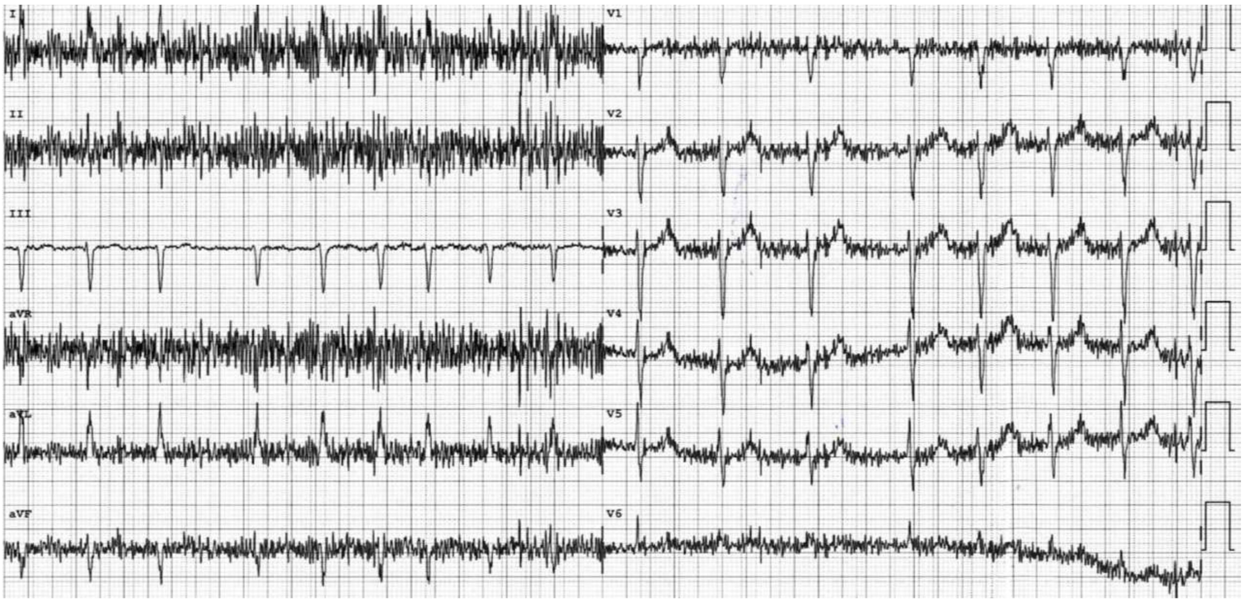

INTERPRET-AF_12-lead_ECG

* 30. How would you classify the following measurement?

- Regular rhythm
- One or more ectopic/missed heartbeats
- Atrial flutter
- Atrial fibrillation
- Unreadable
- Other (please specify)

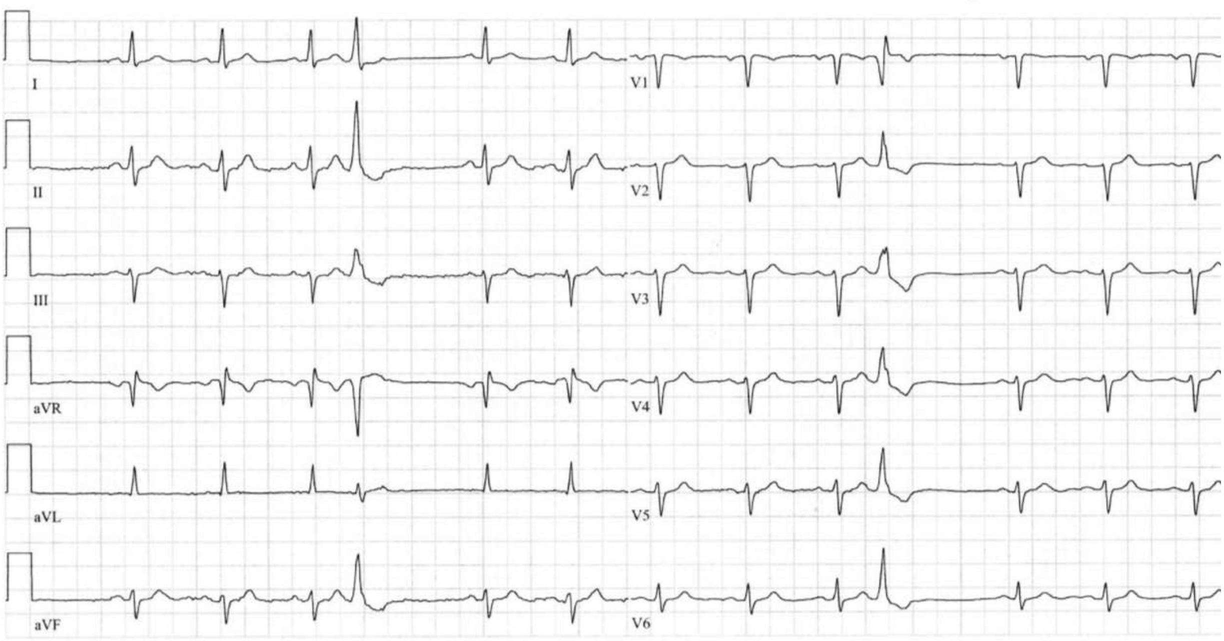

INTERPRET-AF_12-lead_ECG

* 31. How would you classify the following measurement?

- Regular rhythm
- One or more ectopic/missed heartbeats
- Atrial flutter
- Atrial fibrillation
- Unreadable
- Other (please specify)

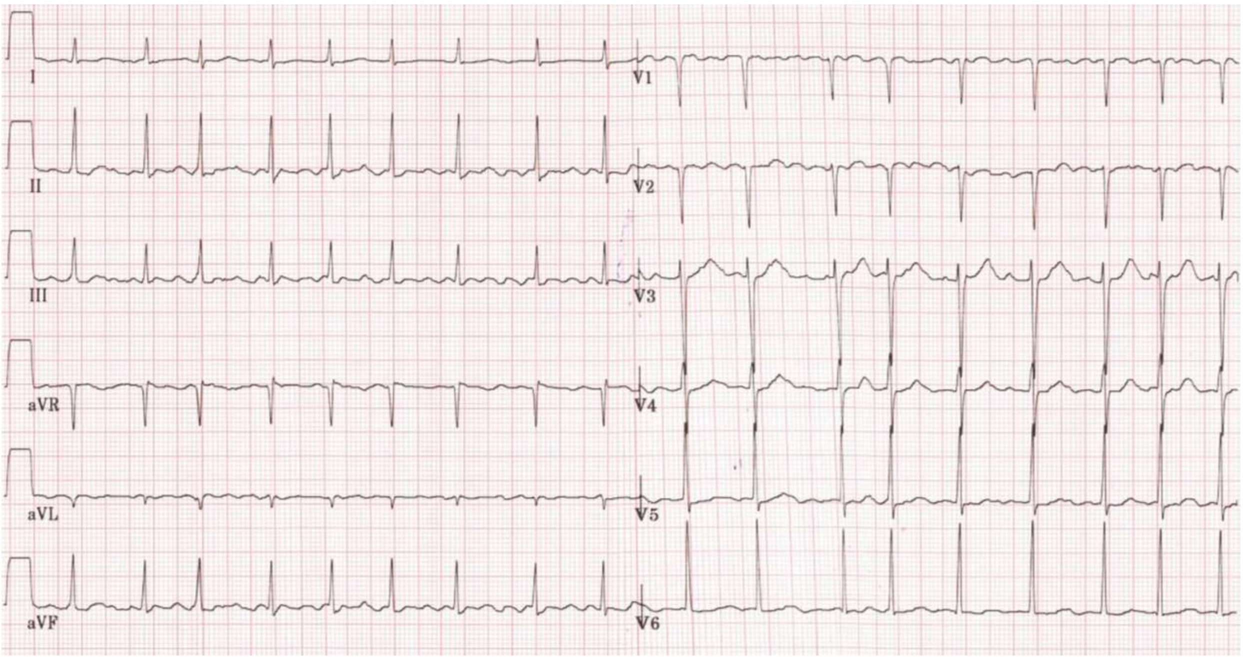

Supplement: Supplementary file 1 [file Data_Sheet_1.PDF]
