# Supplementary material for: Accuracy of Physicians Interpreting Photoplethysmography and Electrocardiography Tracings to Detect Atrial Fibrillation: INTERPRET-AF
Source: Front Cardiovasc Med. 2021 Sep 20;8:734737. doi: 10.3389/fcvm.2021.734737 (PMC8488290; doi:10.3389/fcvm.2021.734737)
Supplement: Supplementary file 2 [file Data_Sheet_2.PDF]

INTERPRET-AF_Single-lead_ECG

* 2. How would you classify the following measurement?

- ◯ Regular rhythm
- ◯ One or more ectopic/missed heartbeats
- ◯ Atrial flutter
- ◯ Atrial fibrillation
- ◯ Unreadable
- ◯ Other (please specify)

Verbeterd filter, Netfilter: 50Hz Scale: 25mm/s, 10mm/mV

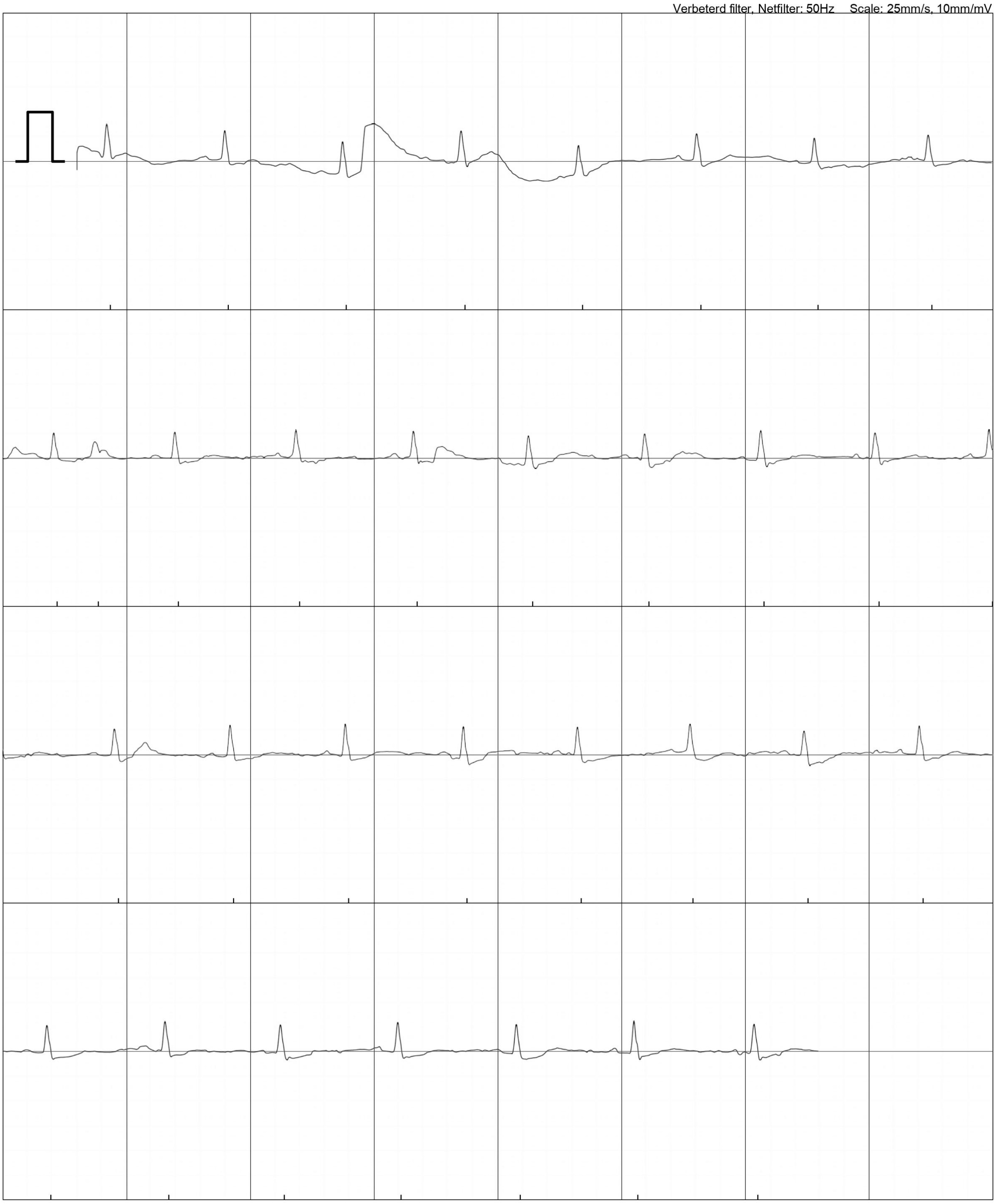

(c) Copyright 2012-2018, AliveCor Inc, Kardia v5.3.0.1584, Report v5.3.0, UUID: A5A9E5B7-5C92-4B3E-8652-815897635834

INTERPRET-AF_Single-lead_ECG

* 3. How would you classify this measurement?

- ◯ Regular rhythm
- ◯ One or more ectopic/missed heartbeats
- ◯ Atrial flutter
- ◯ Atrial fibrillation
- ◯ Unreadable
- ◯ Other (please specify)

Enhanced filter, Netfilter: 50 Hz   Schaal: 25mm/s, 10mm/mV

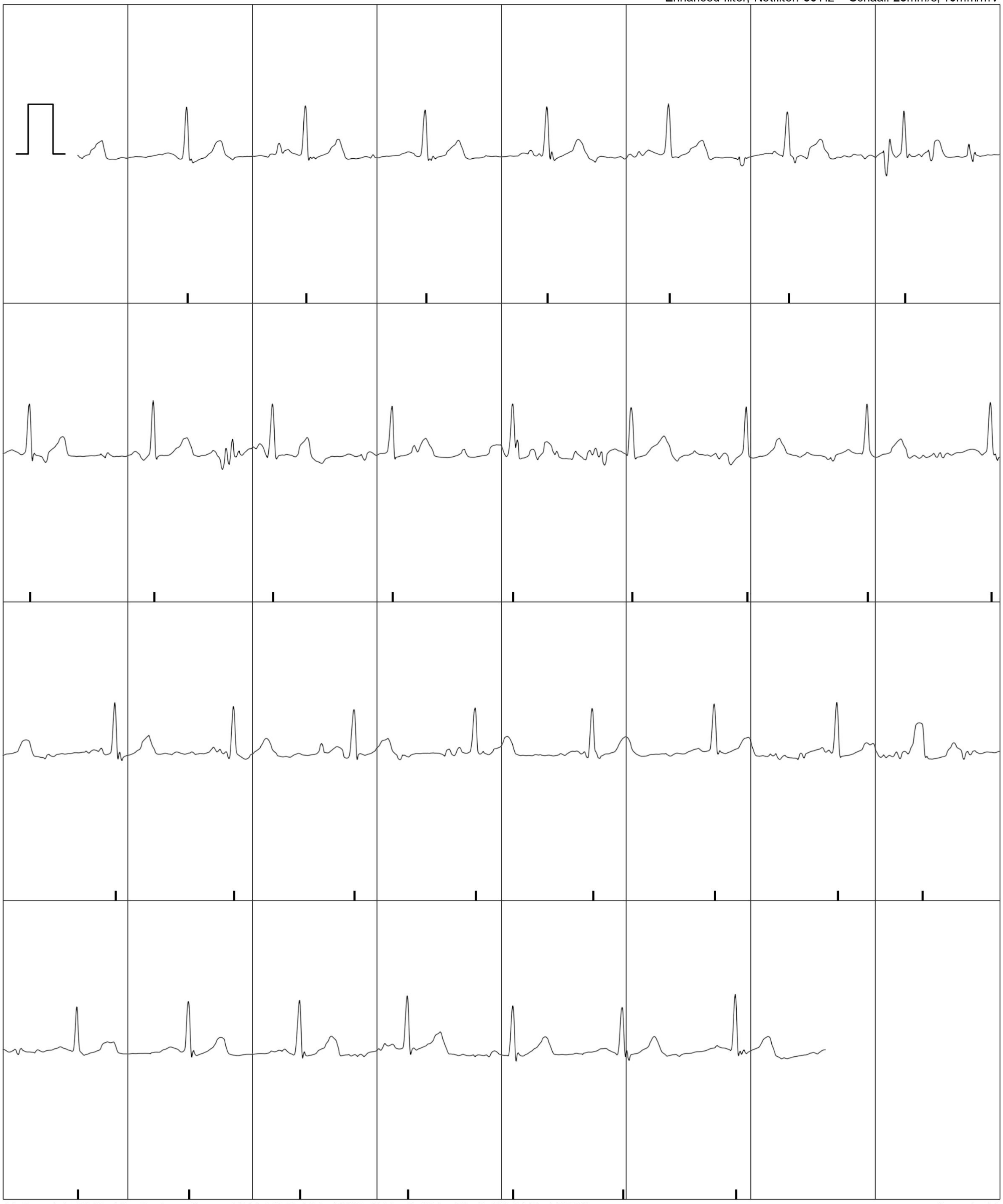

(c) Copyright 2013, AliveCor Inc. aliveecg 5.7.4-06029d739, UUID: d199ccfe-634c-4f4e-bd80-6119cdd932f0

INTERPRET-AF_Single-lead_ECG

* 4. How would you classify the following measurement?

- ◯ Regular rhythm
- ◯ One or more ectopic/missed heartbeats
- ◯ Atrial flutter
- ◯ Atrial fibrillation
- ◯ Unreadable
- ◯ Other (please specify)

Verbeterd filter, Netfilter: 50Hz Scale: 25mm/s, 10mm/mV

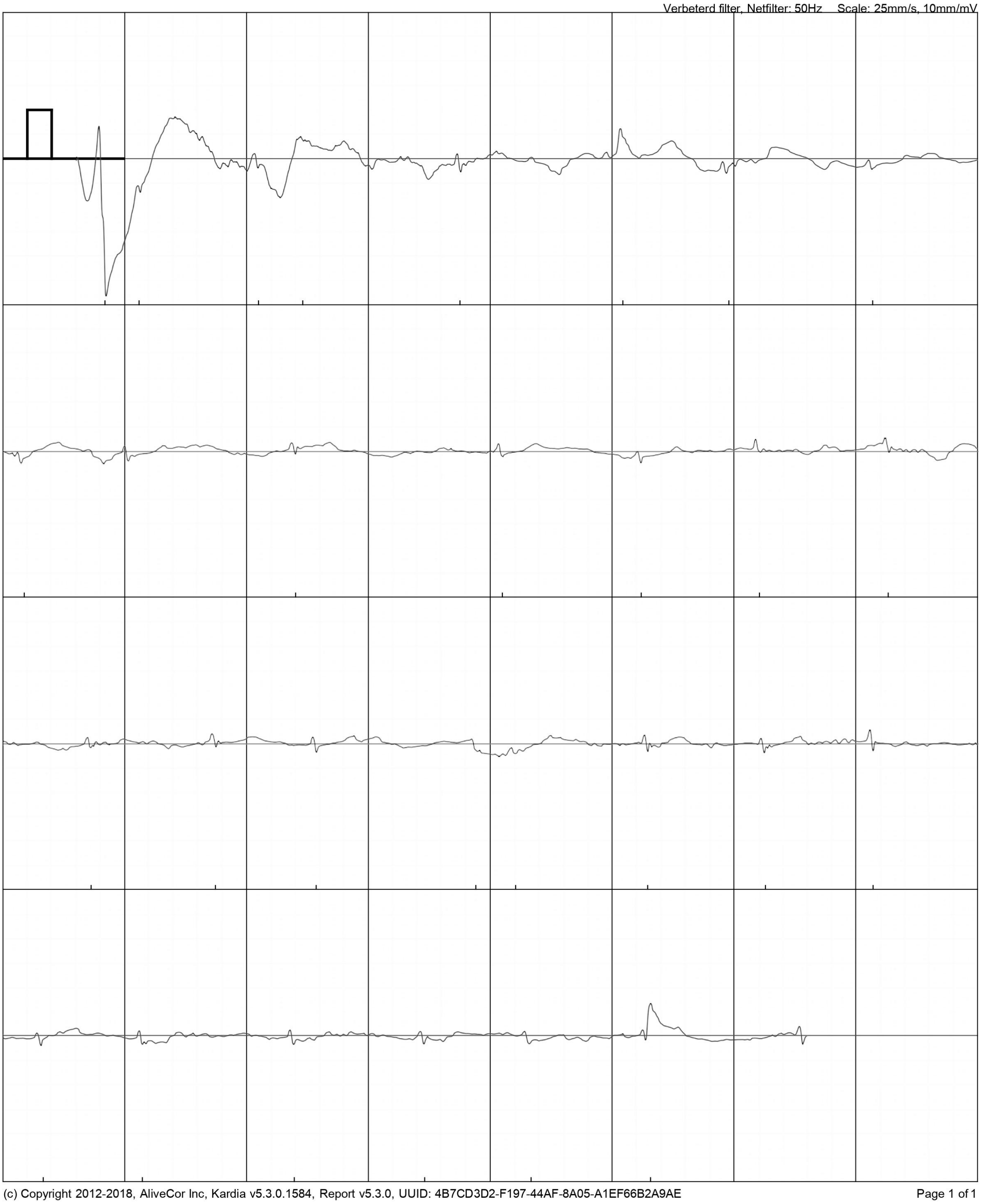

(c) Copyright 2012-2018, AliveCor Inc, Kardia v5.3.0.1584, Report v5.3.0, UUID: 4B7CD3D2-F197-44AF-8A05-A1EF66B2A9AE

INTERPRETET-AF_Single-lead_ECG

* 5. How would you classify the following measurement?

- ○ Regular rhythm
- ○ One or more ectopic/missed heartbeats
- ○ Atrial flutter
- ○ Atrial fibrillation
- ○ Unreadable
- ○ Other (please specify)

Verbeterd filter, Netfilter: 50Hz   Scale: 25mm/s, 10mm/mV

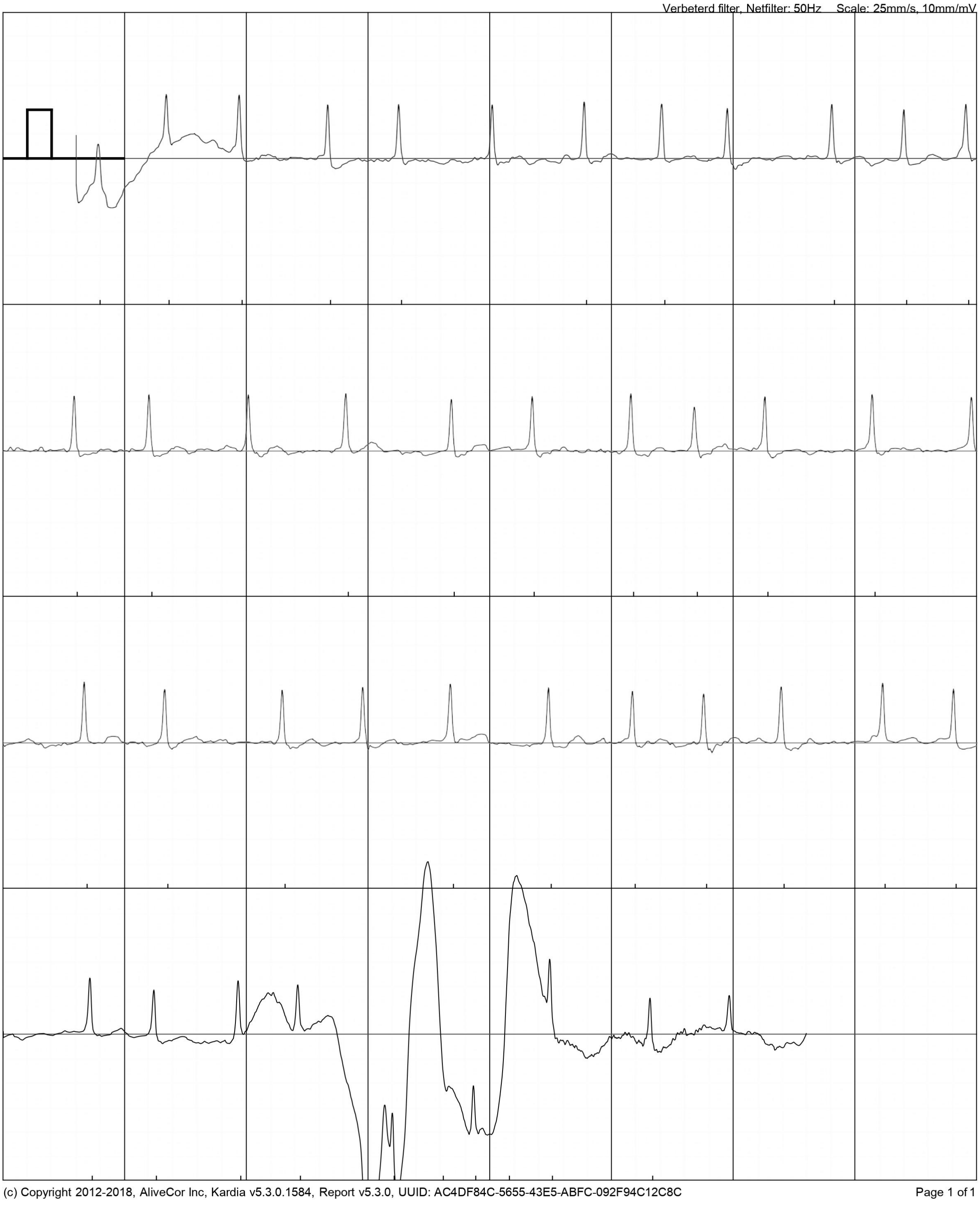

(c) Copyright 2012-2018, AliveCor Inc, Kardia v5.3.0.1584, Report v5.3.0, UUID: AC4DF84C-5655-43E5-ABFC-092F94C12C8C

INTERPRET-AF_Single-lead_ECG

* 6. How would you classify the following measurement?

- Regular rhythm
- One or more ectopic/missed heartbeats
- Atrial flutter
- Atrial fibrillation
- Unreadable
- Other (please specify)

Verbeterd filter, Netfilter: 50Hz   Scale: 25mm/s, 10mm/mV

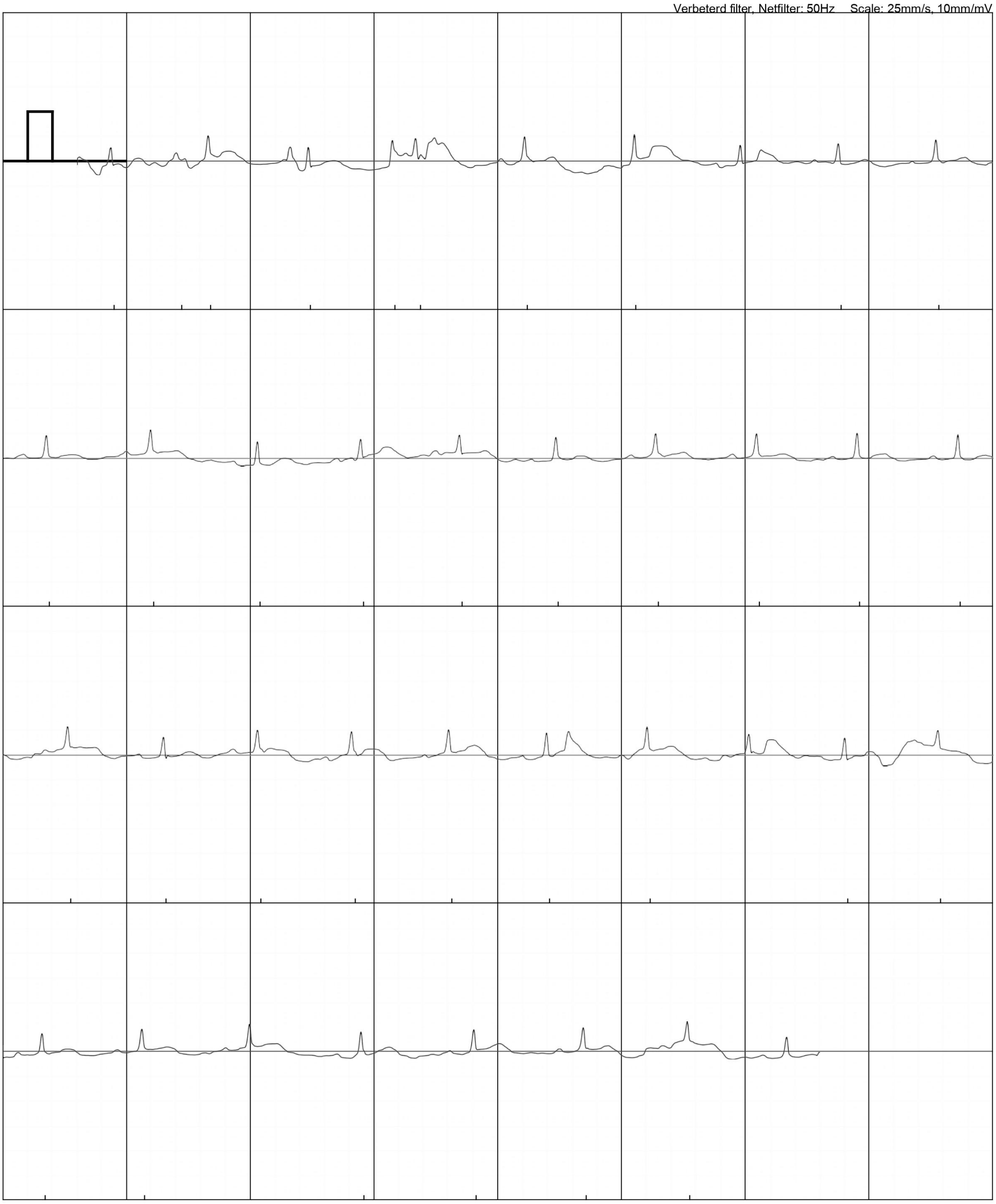

(c) Copyright 2012-2018, AliveCor Inc, Kardia v5.3.0.1584, Report v5.3.0, UUID: 66B31F01-C54B-4A0D-A9DE-BEDEC0CB4643

INTERPRET-AF_Single-lead_ECG

* 7. How would you classify the following measurement?

- Regular rhythm
- One or more ectopic/missed heartbeats
- Atrial flutter
- Atrial fibrillation
- Unreadable
- Other (please specify)

Verbeterd filter, Netfilter: 50Hz Scale: 25mm/s, 10mm/mV

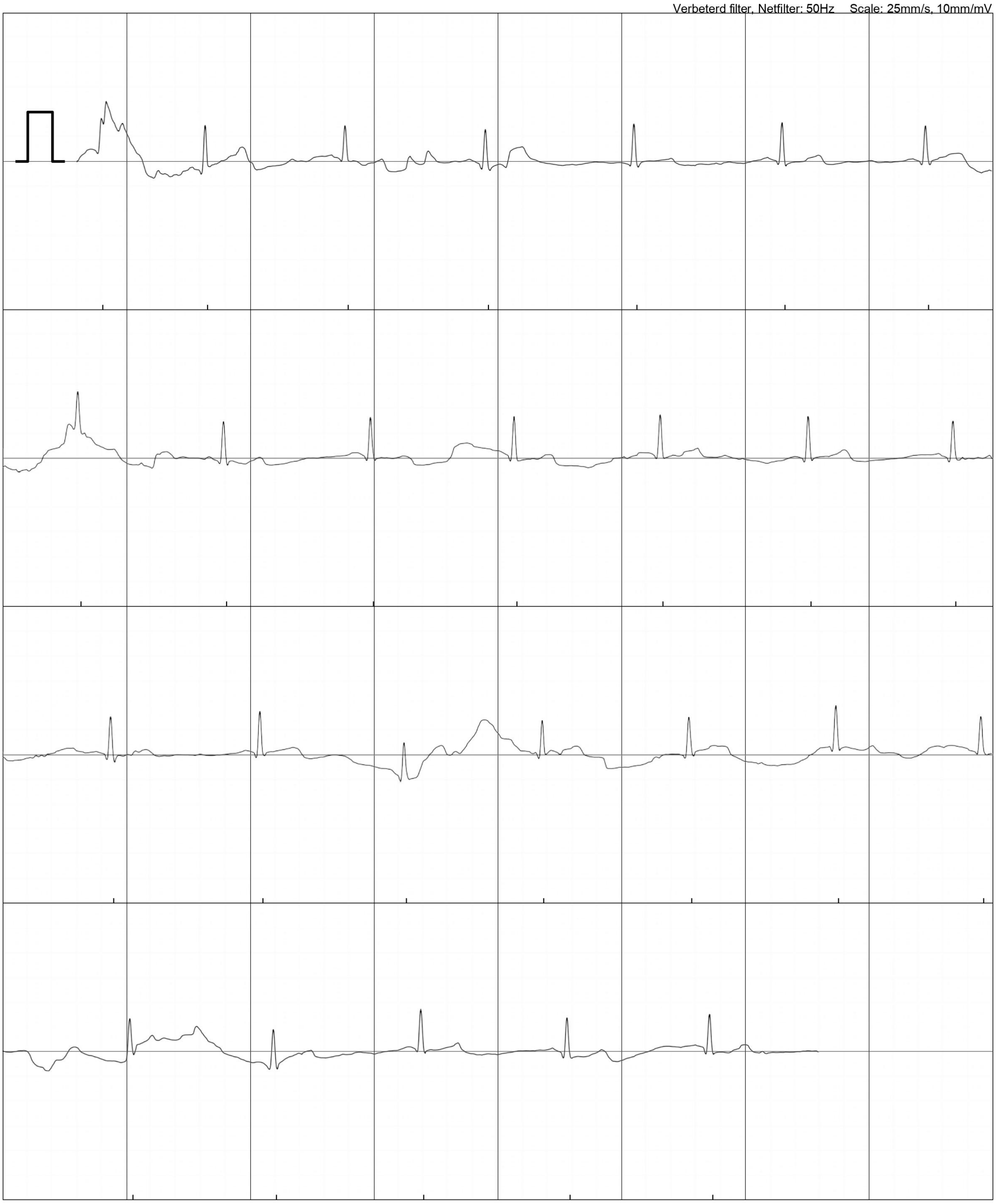

(c) Copyright 2012-2018, AliveCor Inc, Kardia v5.3.0.1584, Report v5.3.0, UUID: 6933603B-F95E-4197-9173-54F18A937A5B

INTERPRET-AF_Single-lead_ECG

* 8. How would you classify the following measurement?

- ◯ Regular rhythm
- ◯ One or more ectopic/missed heartbeats
- ◯ Atrial flutter
- ◯ Atrial fibrillation
- ◯ Unreadable
- ◯ Other (please specify)

Verbeterd filter, Netfilter: 50Hz   Scale: 25mm/s, 10mm/mV

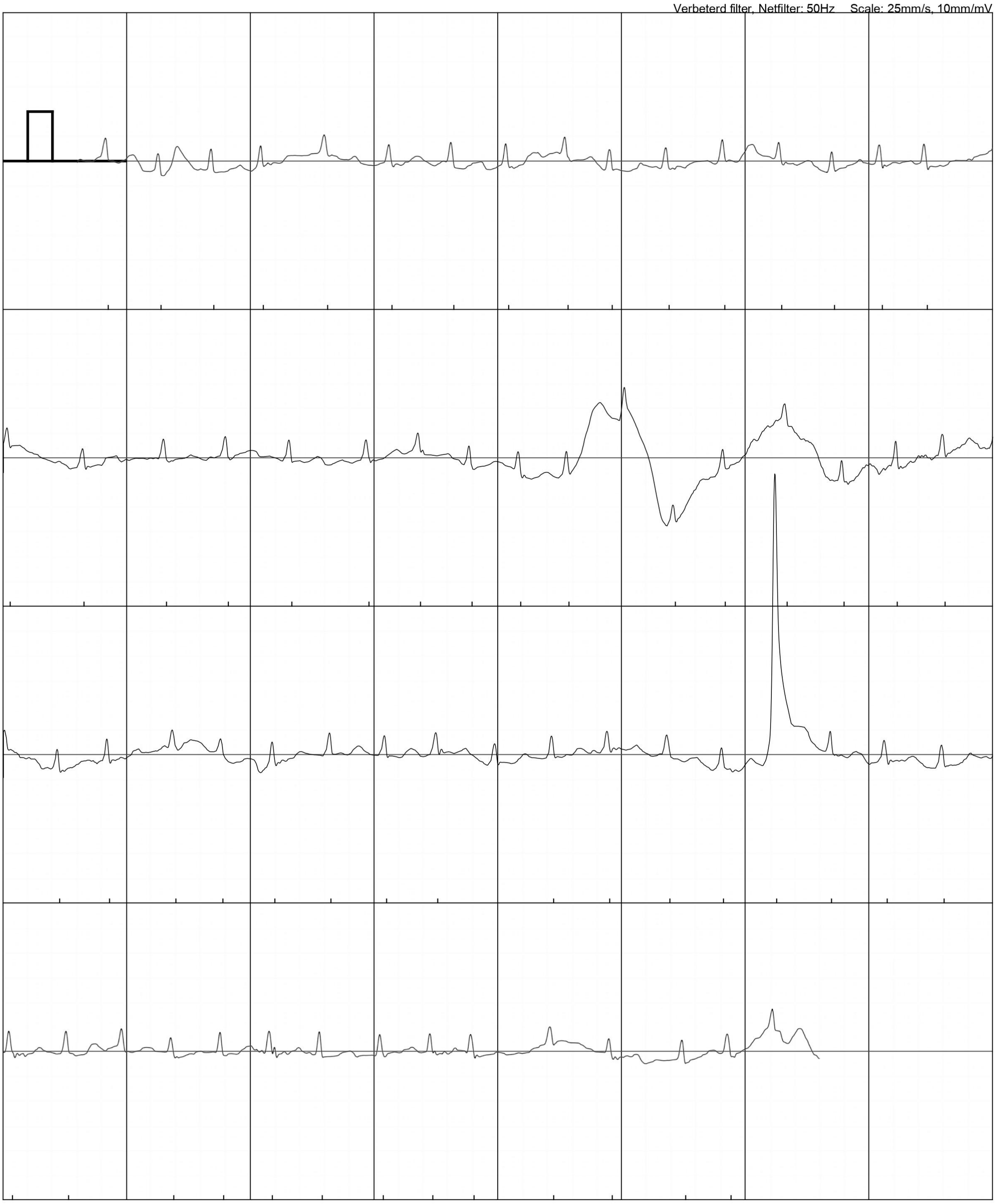

(c) Copyright 2012-2018, AliveCor Inc, Kardia v5.3.0.1584, Report v5.3.0, UUID: 28C36ED9-0166-4478-9A88-A77B33D2C8F8

INTERPRET-AF_Single-lead_ECG

* 9. How would you classify the following measurement?

- ◯ Regular rhythm
- ◯ One or more ectopic/missed heartbeats
- ◯ Atrial flutter
- ◯ Atrial fibrillation
- ◯ Unreadable
- ◯ Other (please specify)

Verbeterd filter, Netfilter: 50Hz   Scale: 25mm/s, 10mm/mV

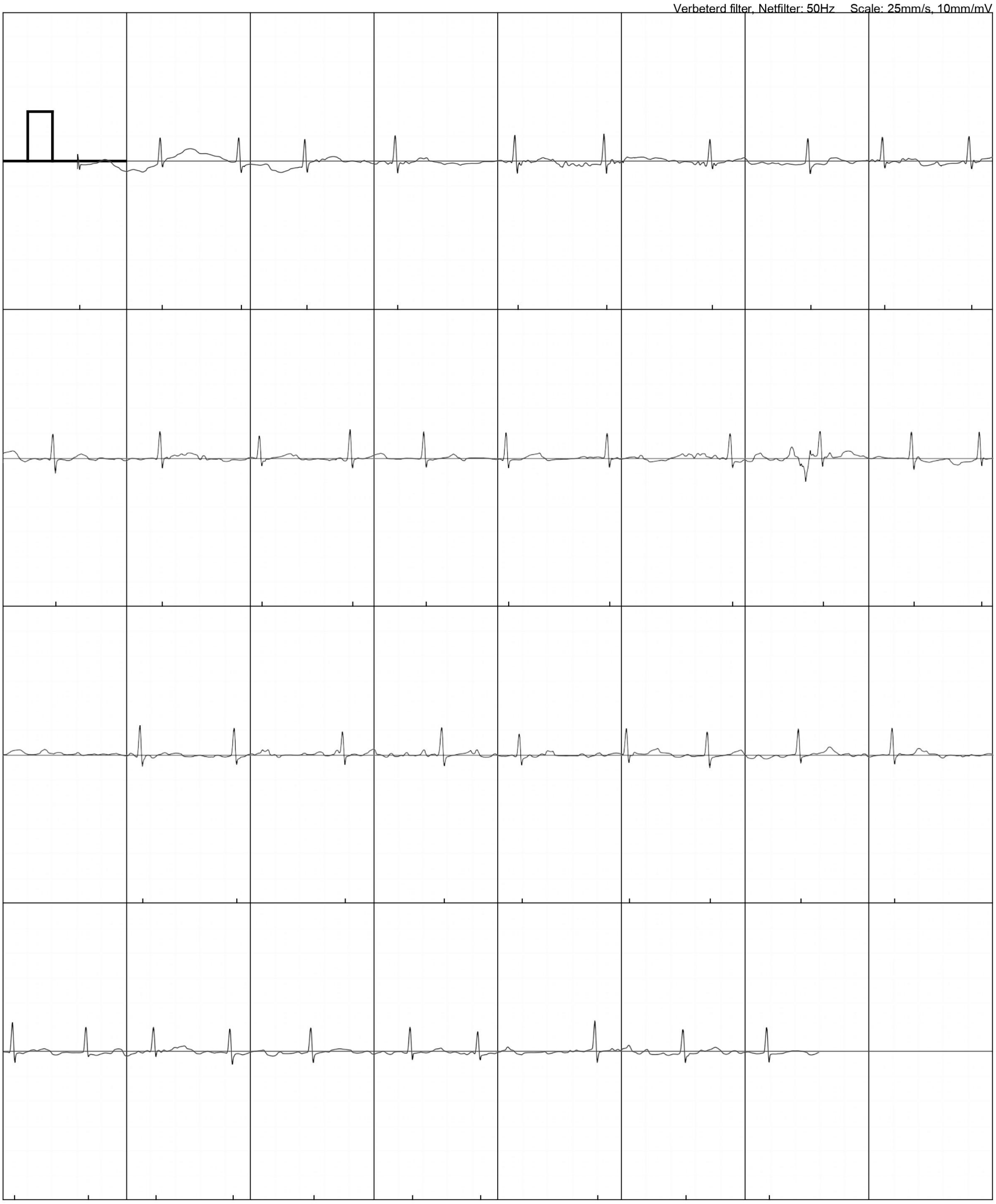

(c) Copyright 2012-2018, AliveCor Inc, Kardia v5.3.0.1584, Report v5.3.0, UUID: BD3626ED-BAE0-4A0F-A268-71989618F88B

INTERPRET-AF_Single-lead_ECG

* 10. How would you classify the following measurement?

- Regular rhythm
- One or more ectopic/missed heartbeats
- Atrial flutter
- Atrial fibrillation
- Unreadable
- Other (please specify)

Verbeterd filter, Netfilter: 50Hz   Scale: 25mm/s, 10mm/mV

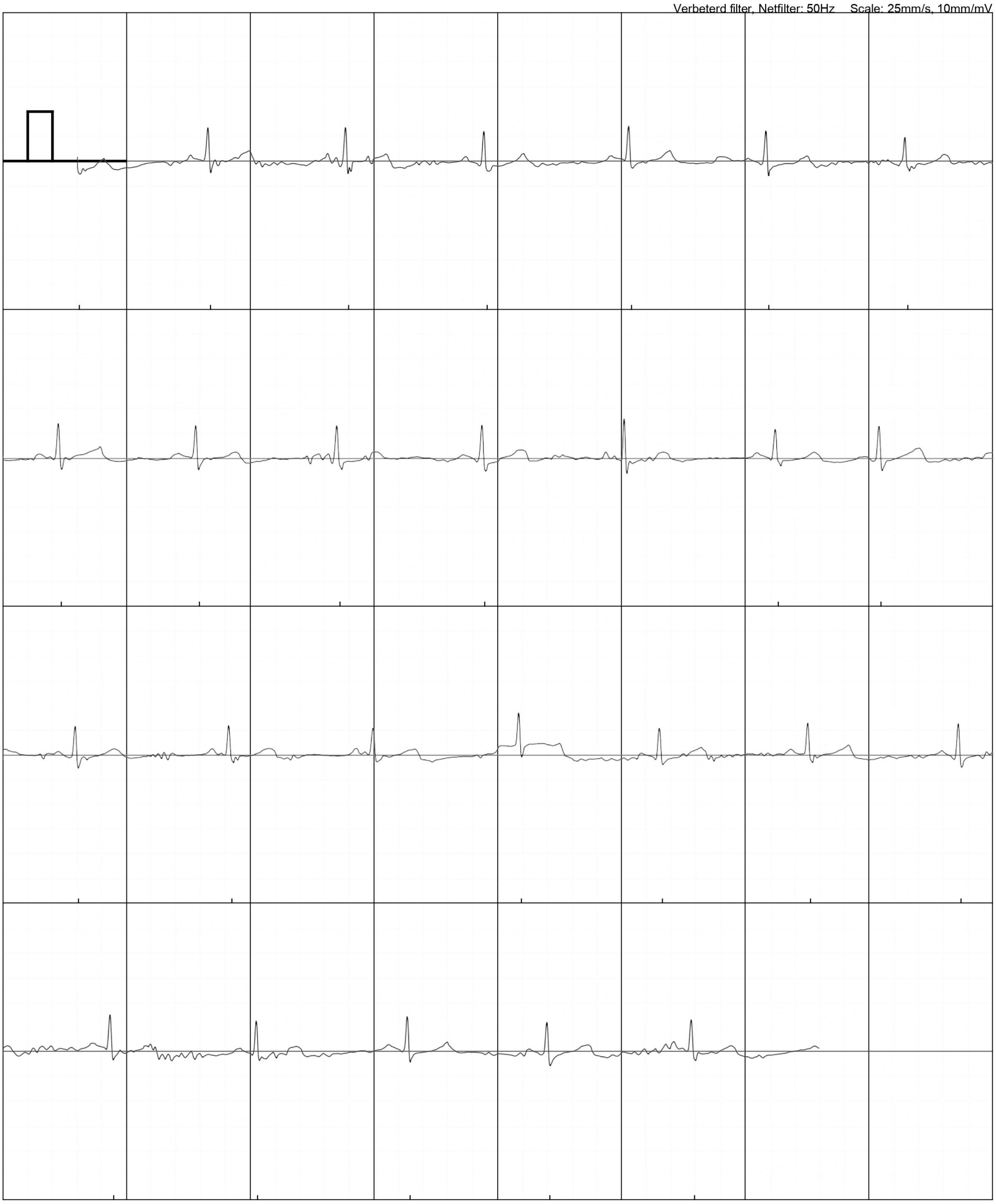

(c) Copyright 2012-2018, AliveCor Inc, Kardia v5.3.0.1584, Report v5.3.0, UUID: DA72C84B-202F-4A28-BC90-7E383EEF965D

INTERPRET-AF_Single-lead_ECG

* 11. How would you classify the following measurement?

- ◯ Regular rhythm
- ◯ One or more ectopic/missed heartbeats
- ◯ Atrial flutter
- ◯ Atrial fibrillation
- ◯ Unreadable
- ◯ Other (please specify)

Verbeterd filter, Netfilter: 50Hz Scale: 25mm/s, 10mm/mV

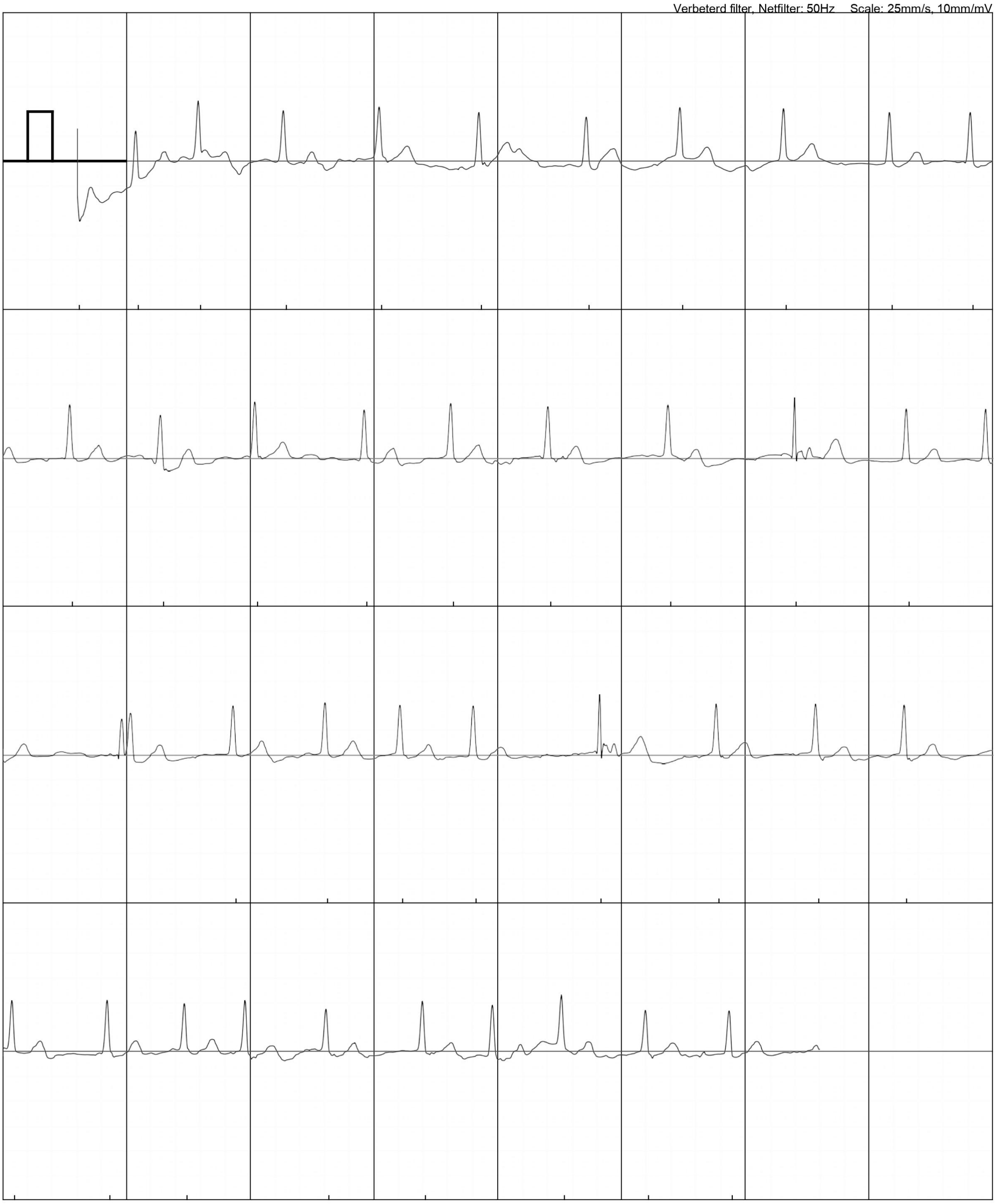

(c) Copyright 2012-2018, AliveCor Inc, Kardia v5.3.0.1584, Report v5.3.0, UUID: 01E0CA33-C9F2-4B3C-A187-D5F8723FEA53

INTERPRET-AF_Single-lead_ECG

* 12. How would you classify the following measurement?

- Regular rhythm
- One or more ectopic/missed heartbeats
- Atrial flutter
- Atrial fibrillation
- Unreadable
- Other (please specify)

Verbeterd filter, Netfilter: 50Hz   Scale: 25mm/s, 10mm/mV

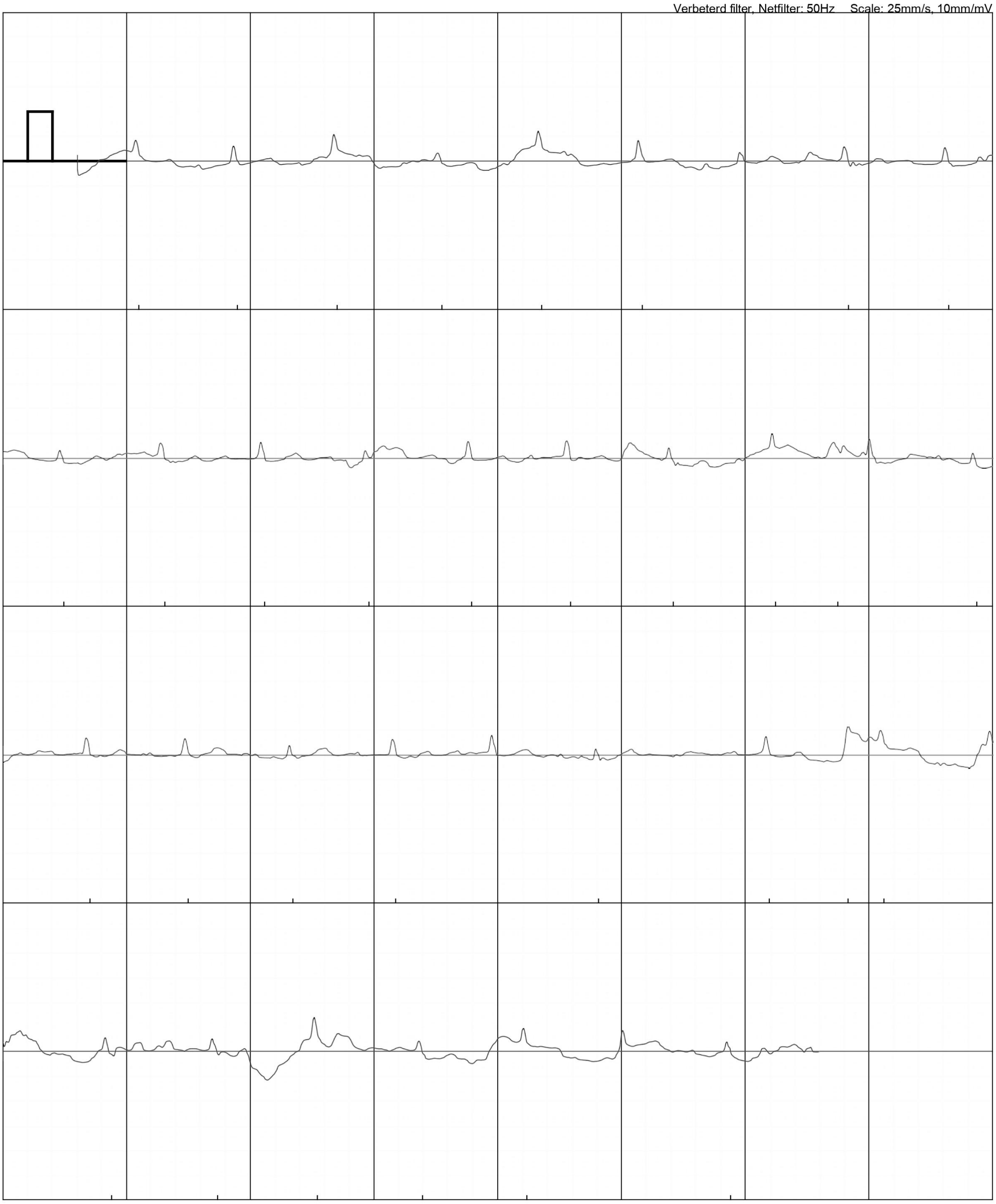

(c) Copyright 2012-2018, AliveCor Inc, Kardia v5.3.0.1584, Report v5.3.0, UUID: B0EE585B-DC6D-4890-8A21-C9AE72DC11CE

INTERPRET-AF_Single-lead_ECG

* 13. How would you classify the following measurement?

- ◯ Regular rhythm
- ◯ One or more ectopic/missed heartbeats
- ◯ Atrial flutter
- ◯ Atrial fibrillation
- ◯ Unreadable
- ◯ Other (please specify)

Verbeterd filter, Netfilter: 50Hz   Scale: 25mm/s, 10mm/mV

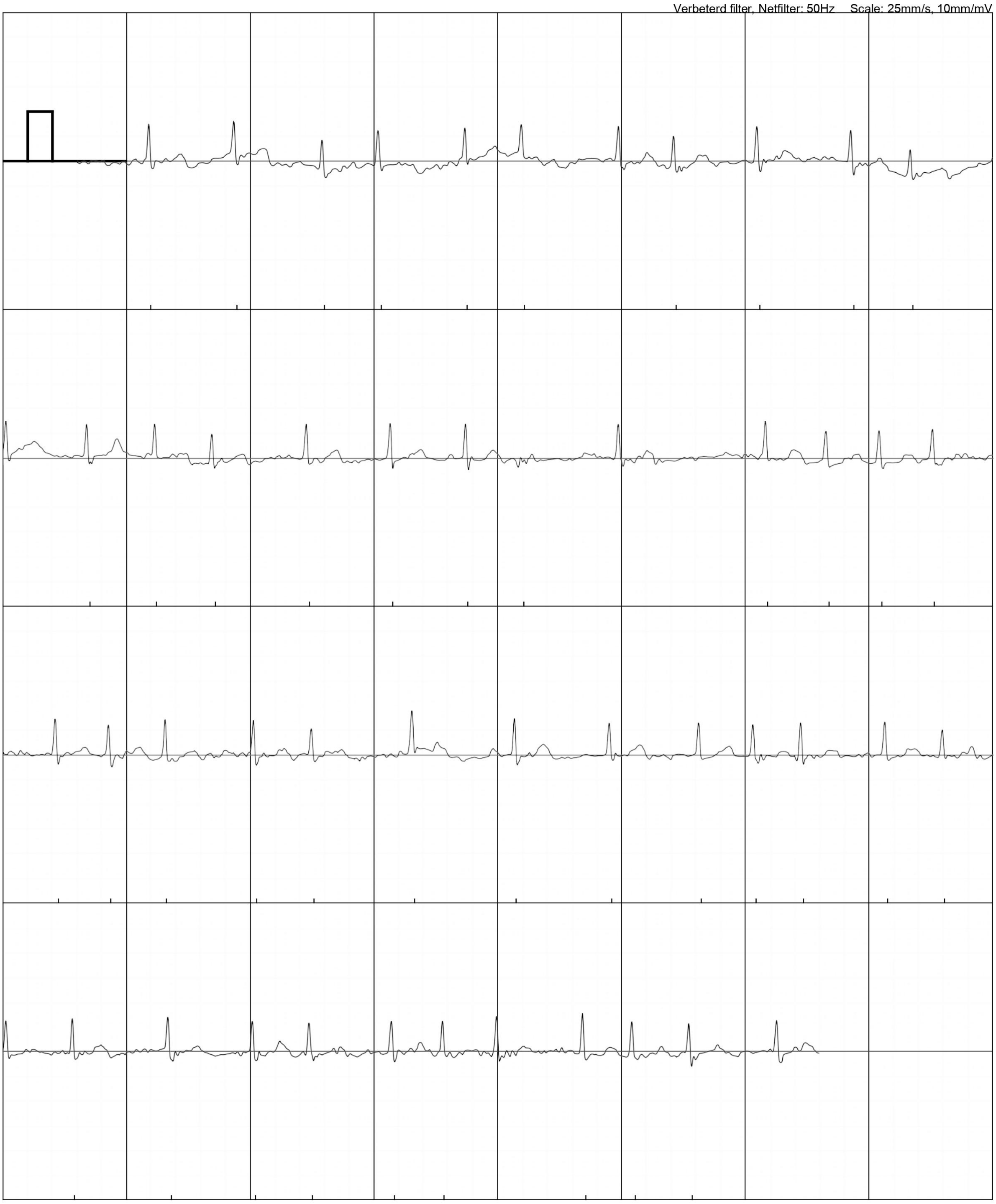

(c) Copyright 2012-2018, AliveCor Inc, Kardia v5.3.0.1584, Report v5.3.0, UUID: 29D6C19C-0F04-404F-ABC0-F26066CE7419

INTERPRET-AF_Single-lead_ECG

* 14. How would you classify the following measurement?

- ◯ Regular rhythm
- ◯ One or more ectopic/missed heartbeats
- ◯ Atrial flutter
- ◯ Atrial fibrillation
- ◯ Unreadable
- ◯ Other (please specify)

Verbeterd filter, Netfilter: 50Hz   Scale: 25mm/s, 10mm/mV

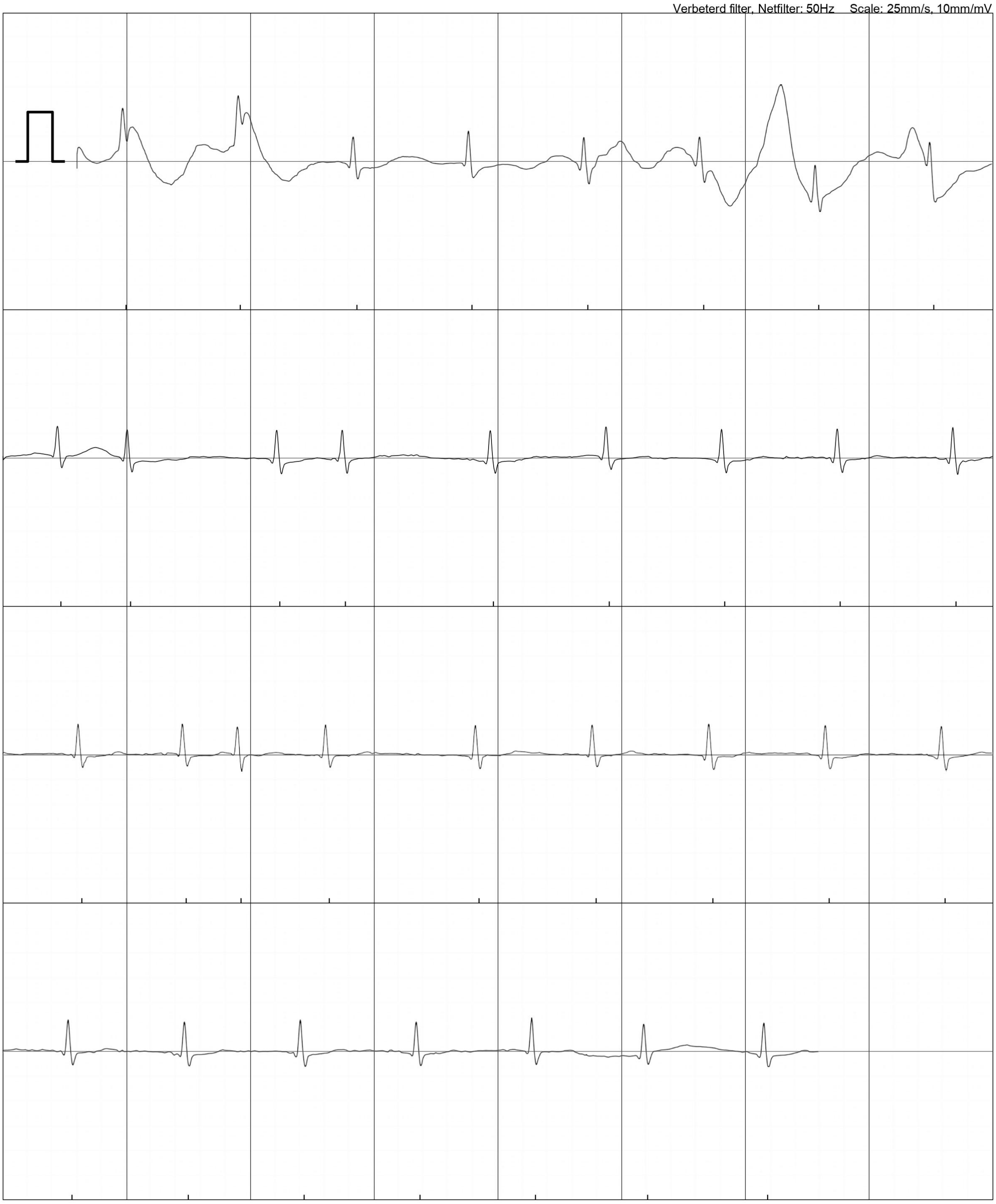

(c) Copyright 2012-2018, AliveCor Inc, Kardia v5.3.0.1584, Report v5.3.0, UUID: A0808763-653C-4370-BD41-829D40FF55C8

INTERPRET-AF_Single-lead_ECG

* 15. How would you classify the following measurement?

- Regular rhythm
- One or more ectopic/missed heartbeats
- Atrial flutter
- Atrial fibrillation
- Unreadable
- Other (please specify)

Verbeterd filter, Netfilter: 50Hz   Scale: 25mm/s, 10mm/mV

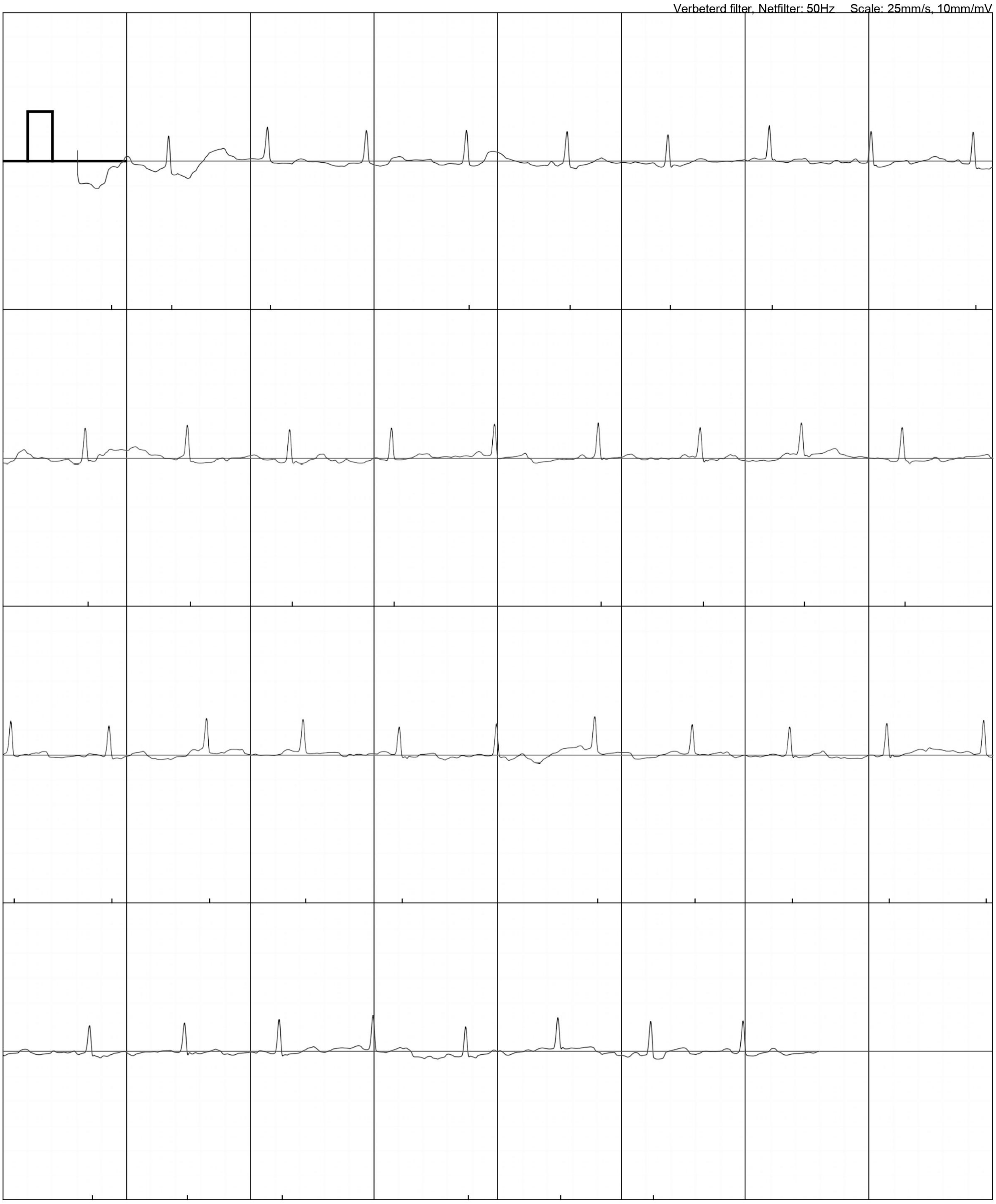

(c) Copyright 2012-2018, AliveCor Inc, Kardia v5.3.0.1584, Report v5.3.0, UUID: 3B7005DA-2D04-4CB7-9909-4937CD849321

INTERPRET-AF_Single-lead_ECG

* 16. How would you classify the following measurement?

- Regular rhythm
- One or more ectopic/missed heartbeats
- Atrial flutter
- Atrial fibrillation
- Unreadable
- Other (please specify)

Verbeterd filter, Netfilter: 50Hz Scale: 25mm/s, 10mm/mV

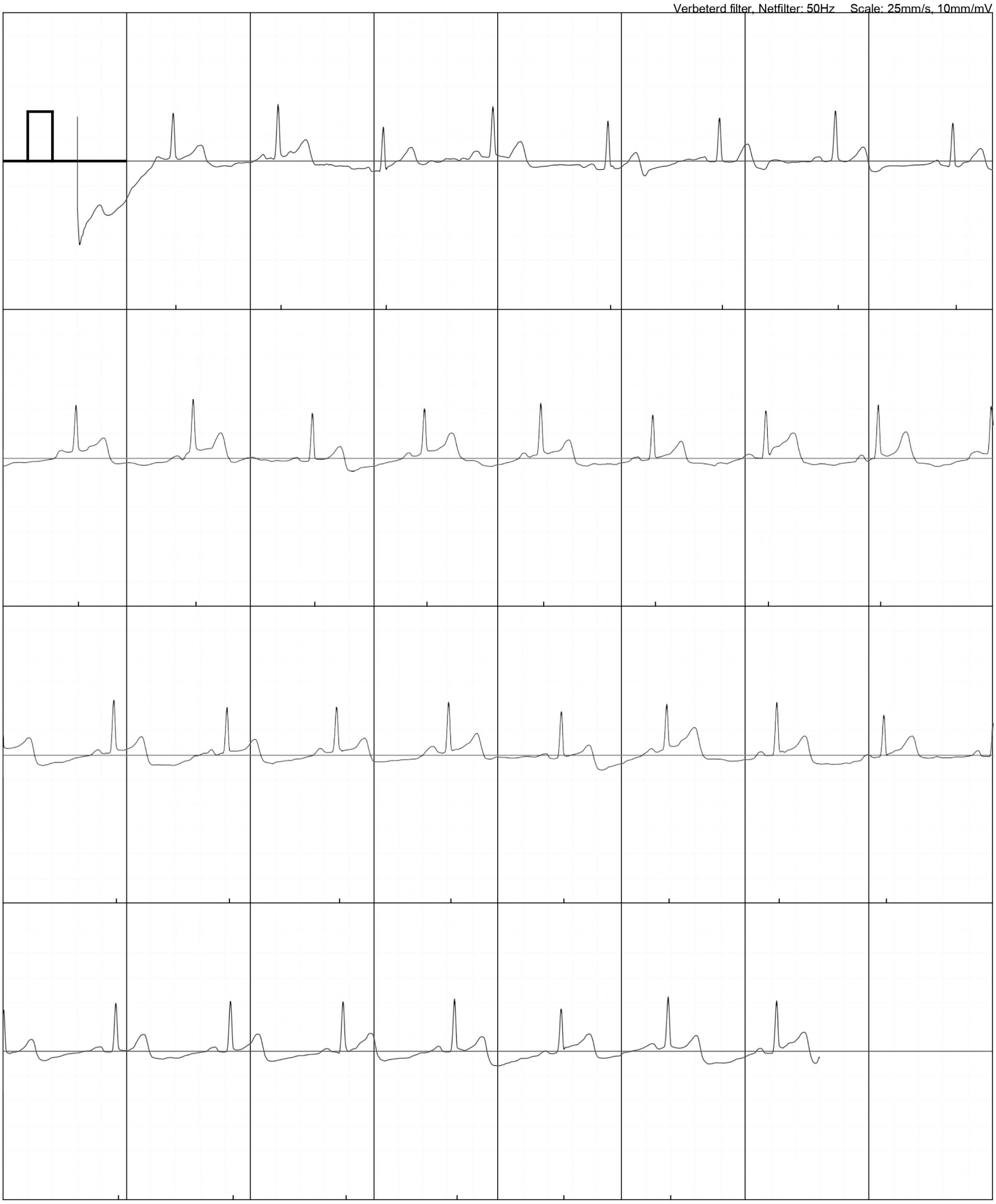

(c) Copyright 2012-2018, AliveCor Inc, Kardia v5.3.0.1584, Report v5.3.0, UUID: 674581F5-1E81-44B6-8BD3-D480261C9E48

INTERPRET-AF_Single-lead_ECG

* 17. How would you classify the following measurement?

- Regular rhythm
- One or more ectopic/missed heartbeats
- Atrial flutter
- Atrial fibrillation
- Unreadable
- Other (please specify)

Verbeterd filter, Netfilter: 50Hz   Scale: 25mm/s, 10mm/mV

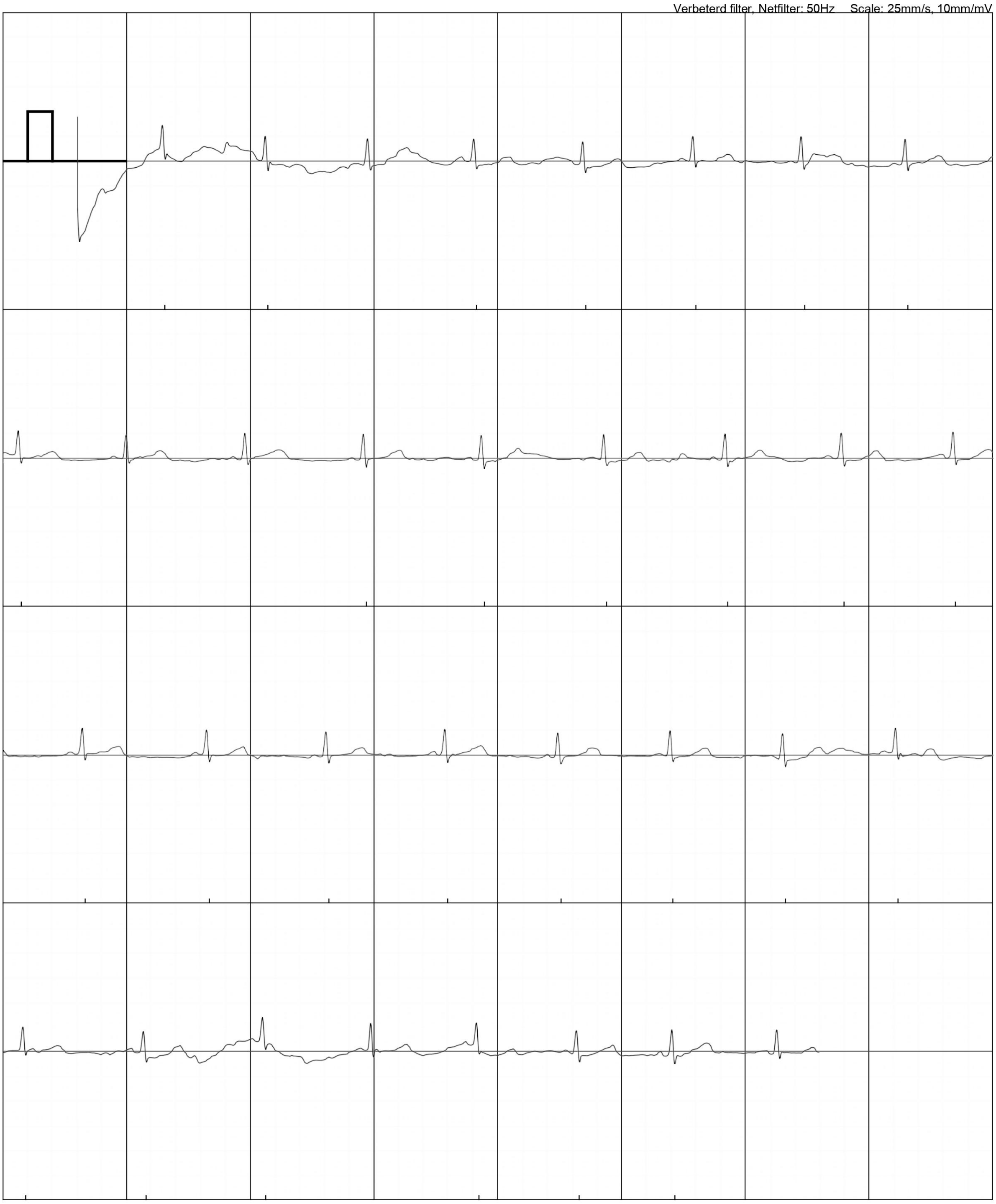

(c) Copyright 2012-2018, AliveCor Inc, Kardia v5.3.0.1584, Report v5.3.0, UUID: CA7DC989-1244-4F1E-B412-4080F39AD37E

INTERPRET-AF_Single-lead_ECG

* 18. How would you classify the following measurement?

- ◯ Regular rhythm
- ◯ One or more ectpic/missed heartbeats
- ◯ Atrial flutter
- ◯ Atrial fibrillation
- ◯ Unreadable
- ◯ Other (please specify)

Verbeterd filter, Netfilter: 50Hz   Scale: 25mm/s, 10mm/mV

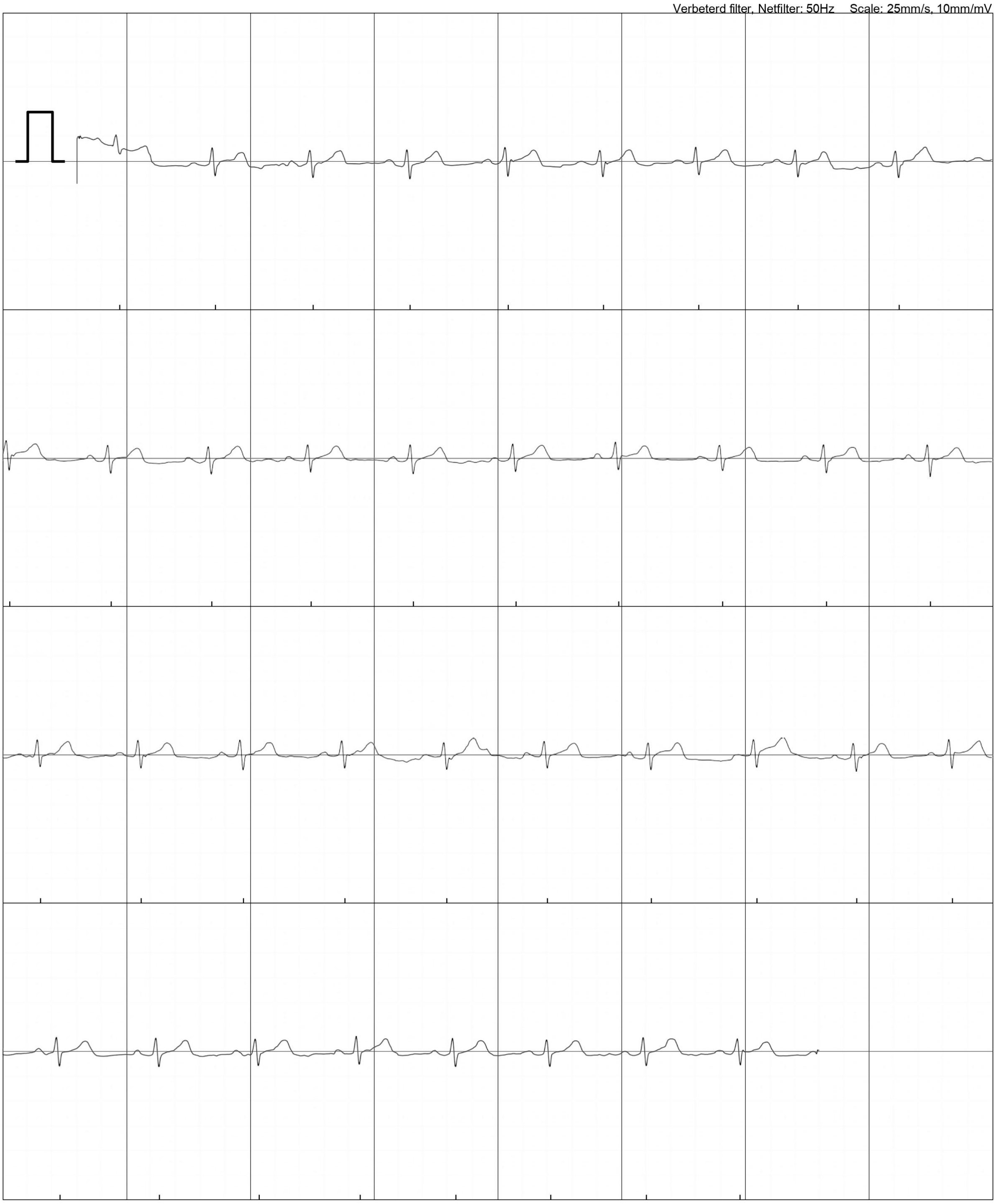

(c) Copyright 2012-2018, AliveCor Inc, Kardia v5.3.0.1584, Report v5.3.0, UUID: 754124C8-4280-4E56-A2F2-6898C6345946

INTERPRET-AF_Single-lead_ECG

* 19. How would you classify the following measurement?

- Regular rhythm
- One or more ectopic/missed heartbeats
- Atrial flutter
- Atrial fibrillation
- Unreadable
- Other (please specify)

Verbeterd filter, Netfilter: 50Hz   Scale: 25mm/s, 10mm/mV

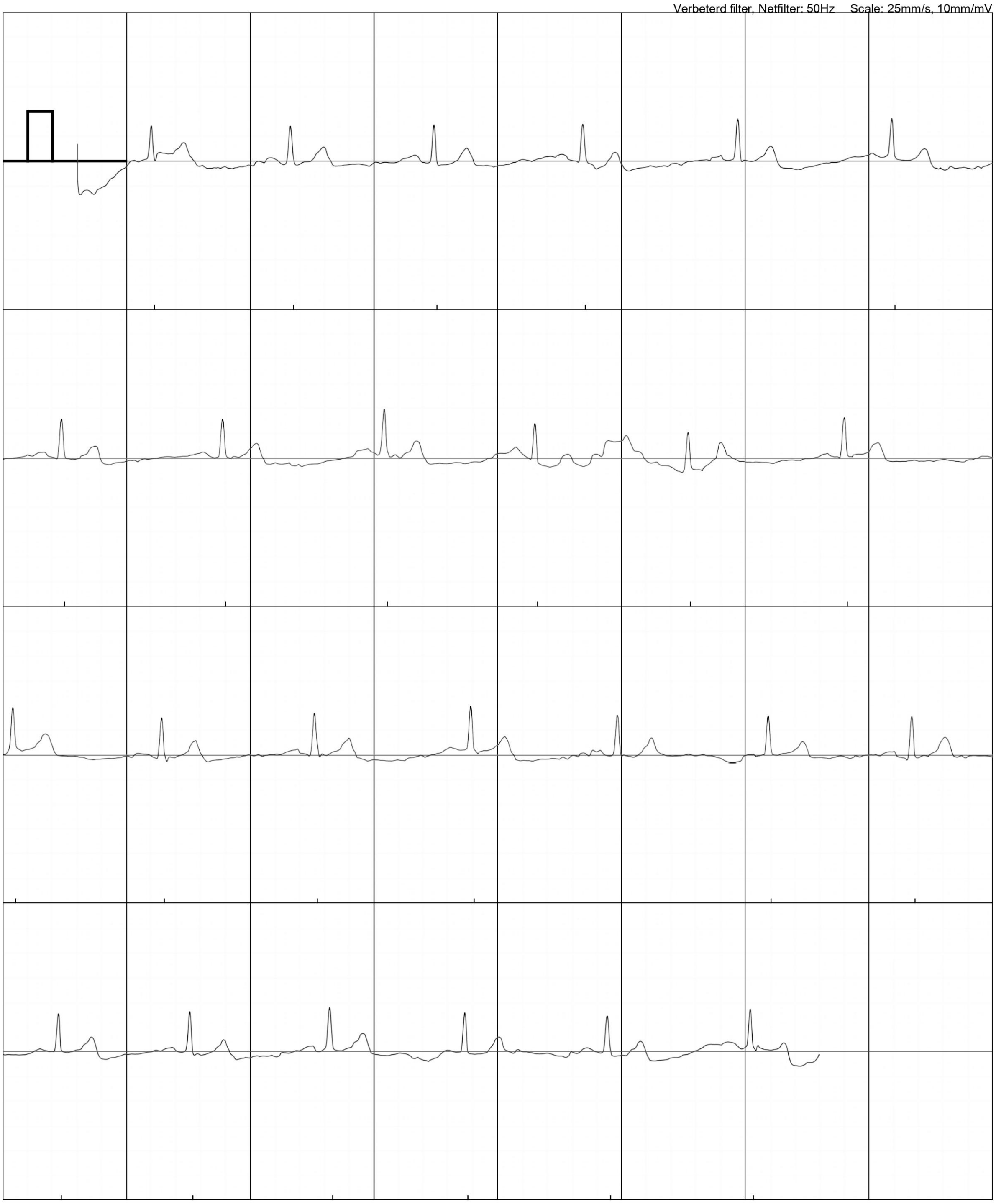

(c) Copyright 2012-2018, AliveCor Inc, Kardia v5.3.0.1584, Report v5.3.0, UUID: 7681EDB3-57B5-4343-8645-5C5BBD6037ED

INTERPRET-AF_Single-lead_ECG

* 20. How would you classify the following measurement?

- Regular rhythm
- One or more ectopic/missed heartbeats
- Atrial flutter
- Atrial fibrillation
- Unreadable
- Other (please specify)

Verbeterd filter, Netfilter: 50Hz Scale: 25mm/s, 10mm/mV

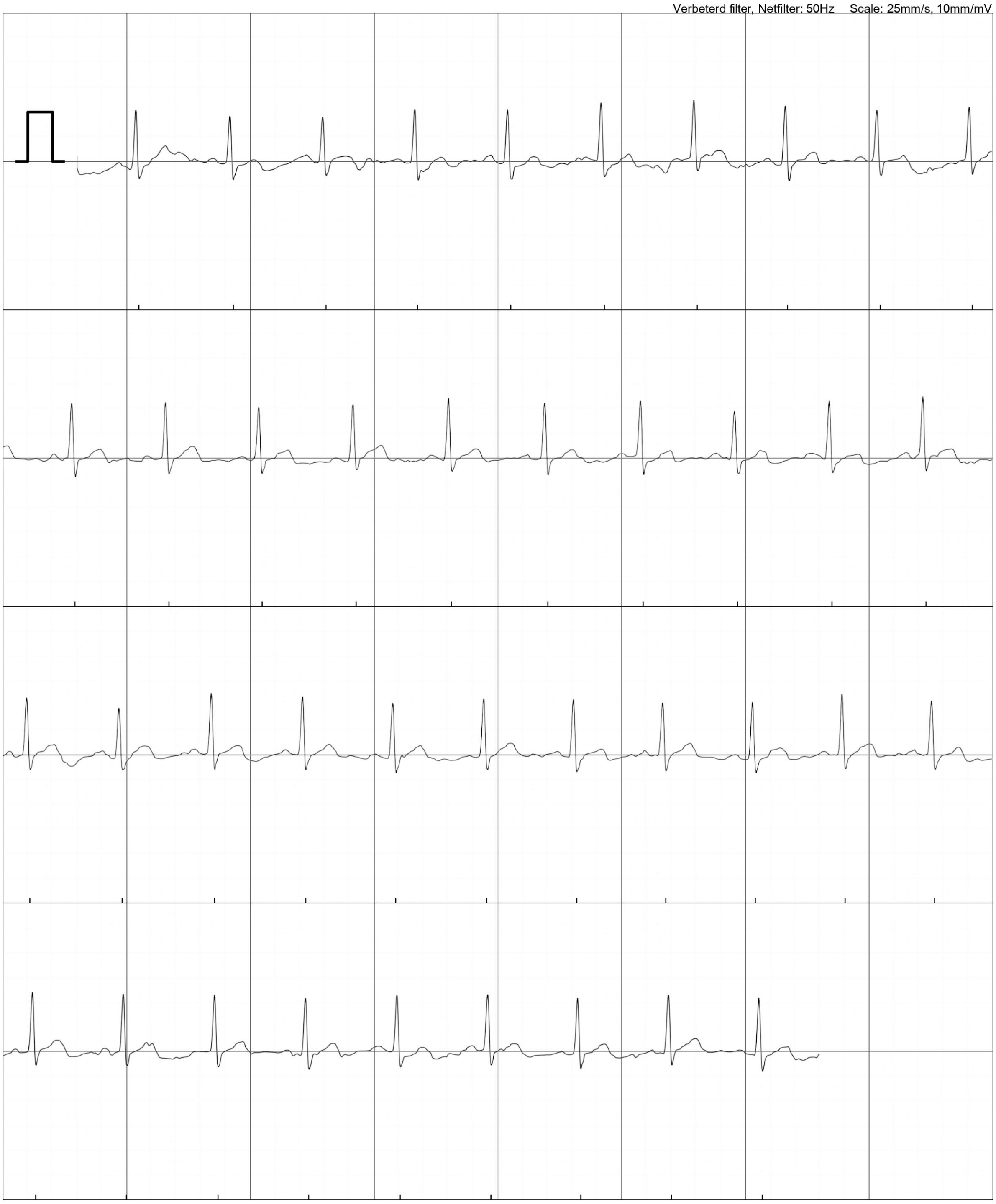

(c) Copyright 2012-2018, AliveCor Inc, Kardia v5.3.0.1584, Report v5.3.0, UUID: 9B7766B3-2526-47DF-99C4-BB38137349BE

INTERPRET-AF_Single-lead_ECG

* 21. How would you classify the following measurement?

- Regular rhythm
- One or more ectopic/missed heartbeats
- Atrial flutter
- Atrial fibrillation
- Unreadable
- Other (please specify)

Scale: 25mm/s, 10mm/mV

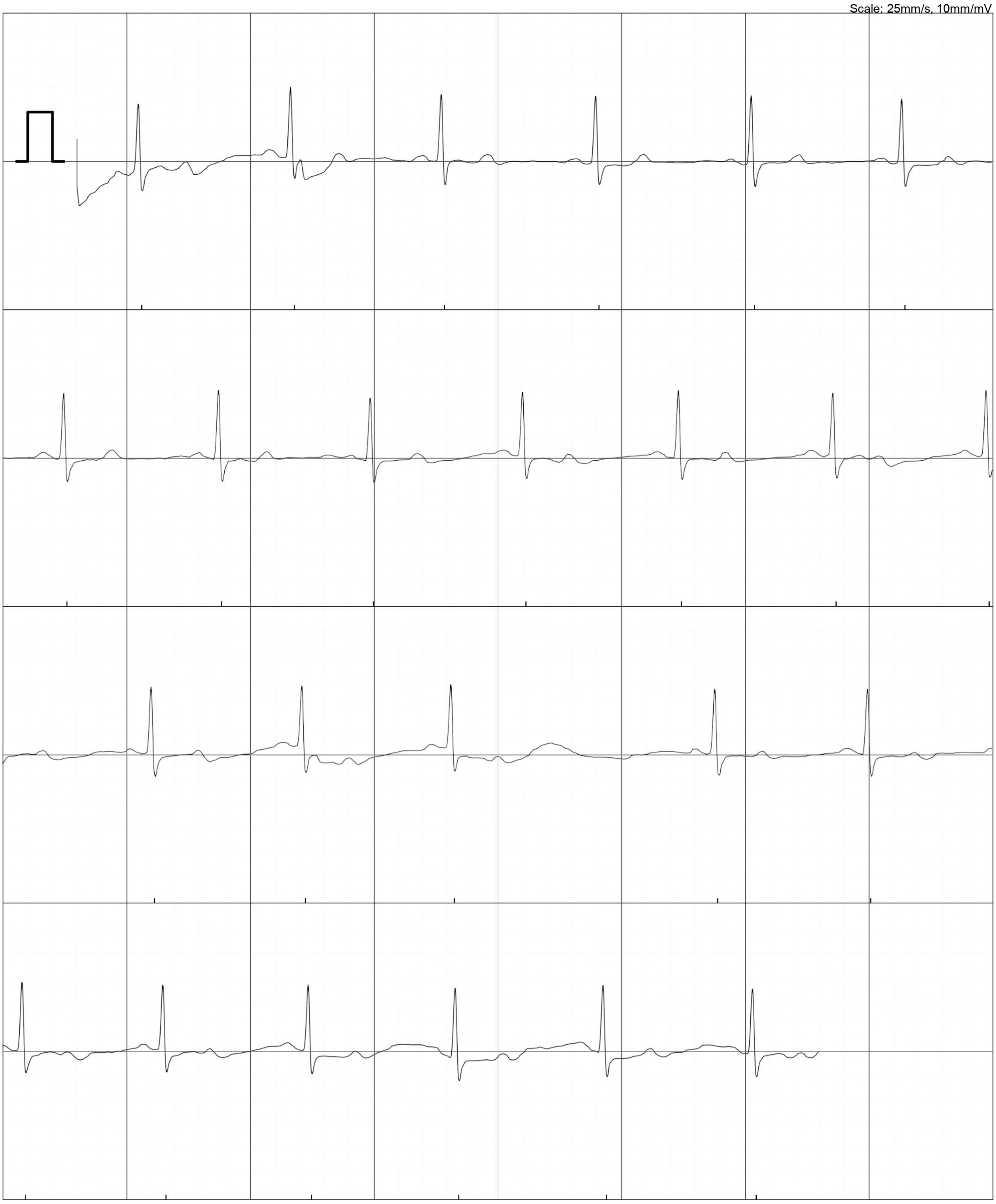

(c) Copyright 2012-2018, AliveCor Inc, Kardia v5.3.0.1584, Report v5.3.0, UUID: 479C6533-28CE-41E7-A5E8-839AF2190560

INTERPRET-AF_Single-lead_ECG

* 22. How would you classify the following measurement?

- ◯ Regular rhythm
- ◯ One or more ectopic/missed heartbeats
- ◯ Atrial flutter
- ◯ Atrial fibrillation
- ◯ Unreadable
- ◯ Other (please specify)

Verbeterd filter, Netfilter: 50Hz Scale: 25mm/s, 10mm/mV

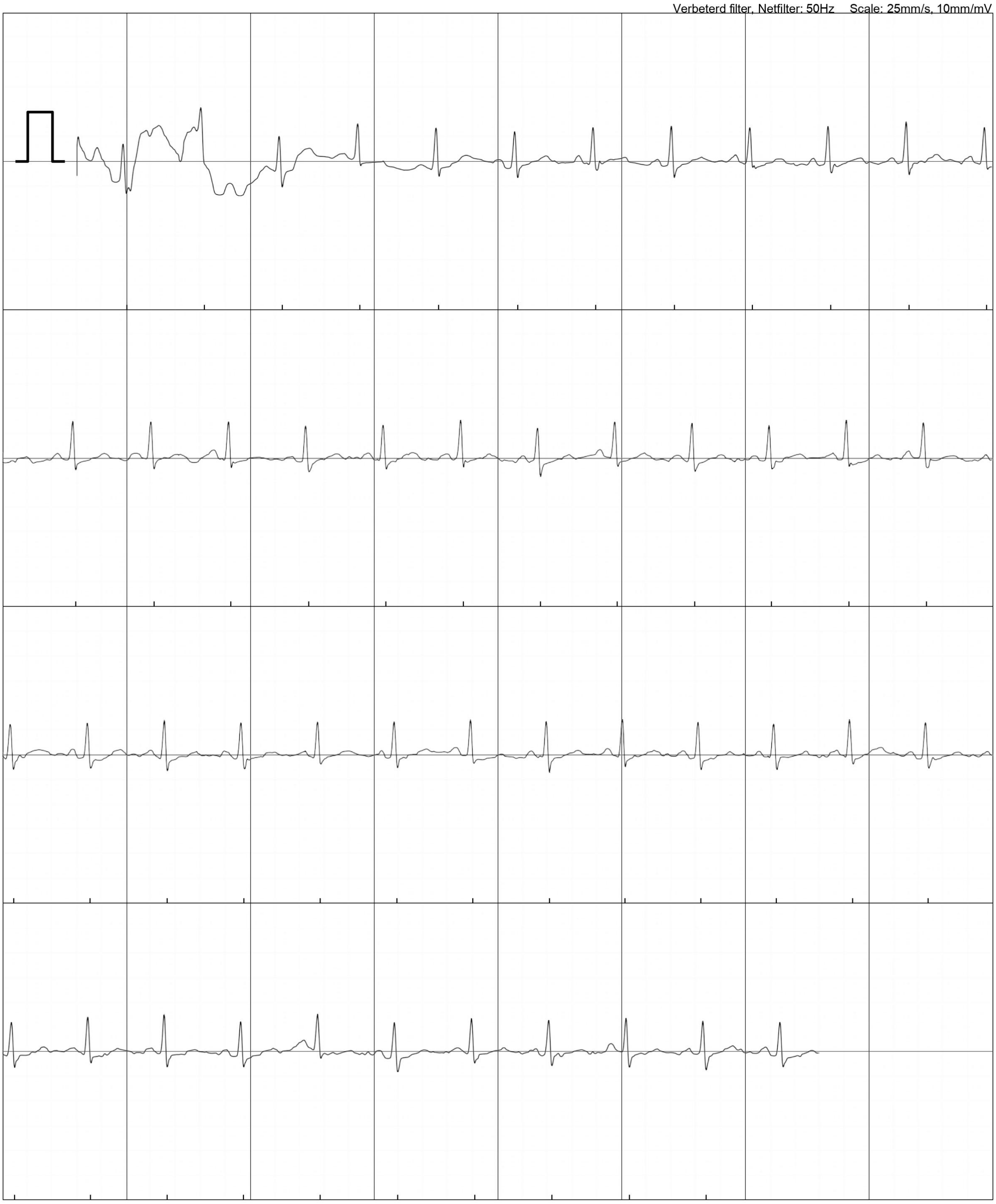

(c) Copyright 2012-2018, AliveCor Inc, Kardia v5.3.0.1584, Report v5.3.0, UUID: 2DB722D9-93E7-4A21-9F66-E67DFF49BF58

INTERPRET-AF_Single-lead_ECG

* 23. How would you classify the following measurement?

- ◯ Regular rhythm
- ◯ One or more ectopic/missed heartbeats
- ◯ Atrial flutter
- ◯ Atrial fibrillation
- ◯ Unreadable
- ◯ Other (please specify)

Verbeterd filter, Netfilter: 50Hz Scale: 25mm/s, 10mm/mV

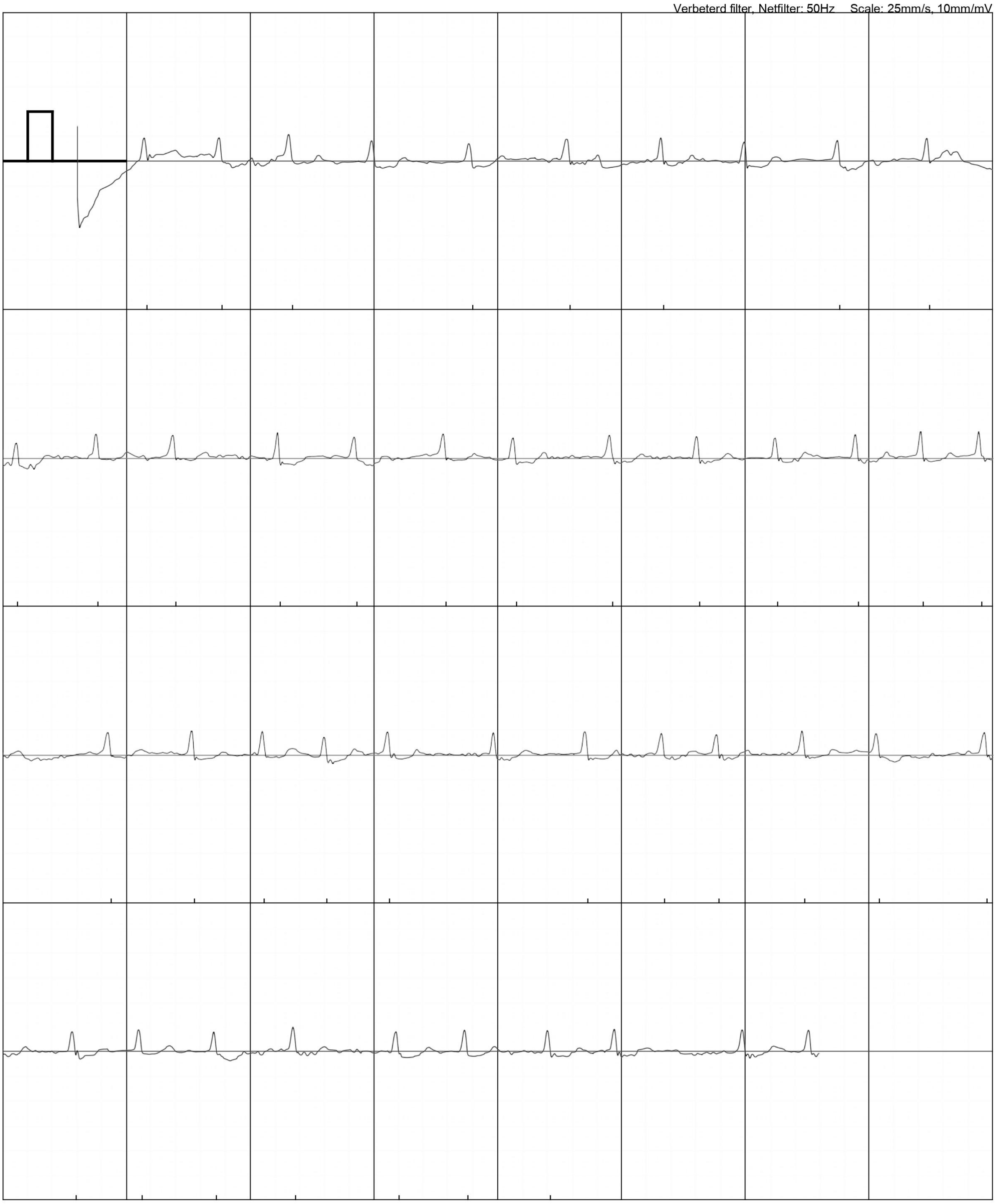

(c) Copyright 2012-2018, AliveCor Inc, Kardia v5.3.0.1584, Report v5.3.0, UUID: 0A7BCF2C-2166-46F7-ADE1-51522517A253

INTERPRET-AF_Single-lead_ECG

* 24. How would you classify the following measurement?

- ◯ Regular rhythm
- ◯ One or more ectopic/missed heartbeats
- ◯ Atrial flutter
- ◯ Atrial fibrillation
- ◯ Unreadable
- ◯ Other (please specify)

Verbeterd filter, Netfilter: 50Hz   Scale: 25mm/s, 10mm/mV

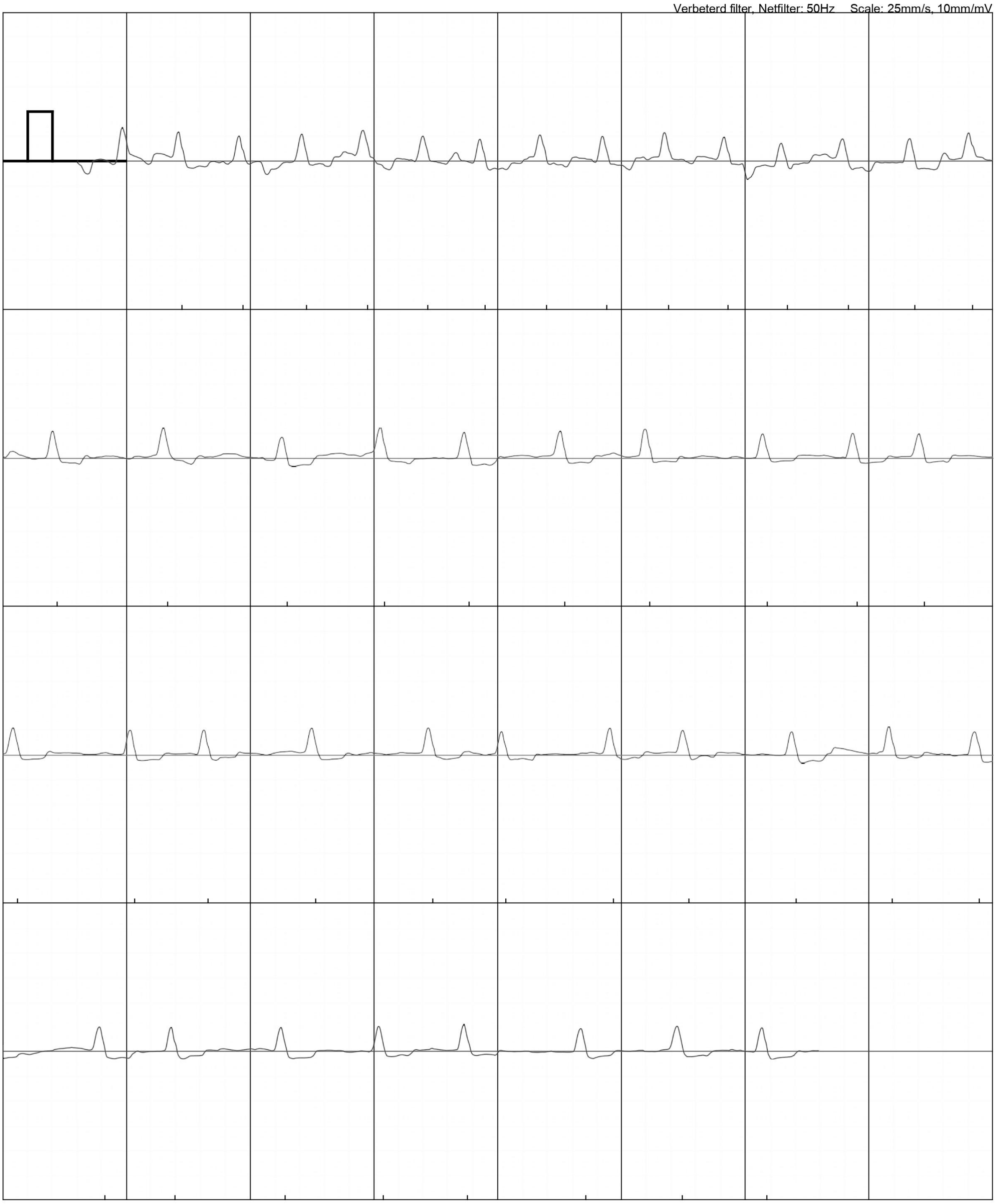

(c) Copyright 2012-2018, AliveCor Inc, Kardia v5.3.0.1584, Report v5.3.0, UUID: 760642A8-86E6-4CF5-80C4-918BEE8D9185

INTERPRET-AF_Single-lead_ECG

* 25. How would you classify the following measurement?

- ◯ Regular rhythm
- ◯ One or more ectopic/missed heartbeats
- ◯ Atrial flutter
- ◯ Atrial fibrillation
- ◯ Unreadable
- ◯ Other (please specify)

Enhanced filter, Netfilter: 50 Hz   Schaal: 25mm/s, 10mm/mV

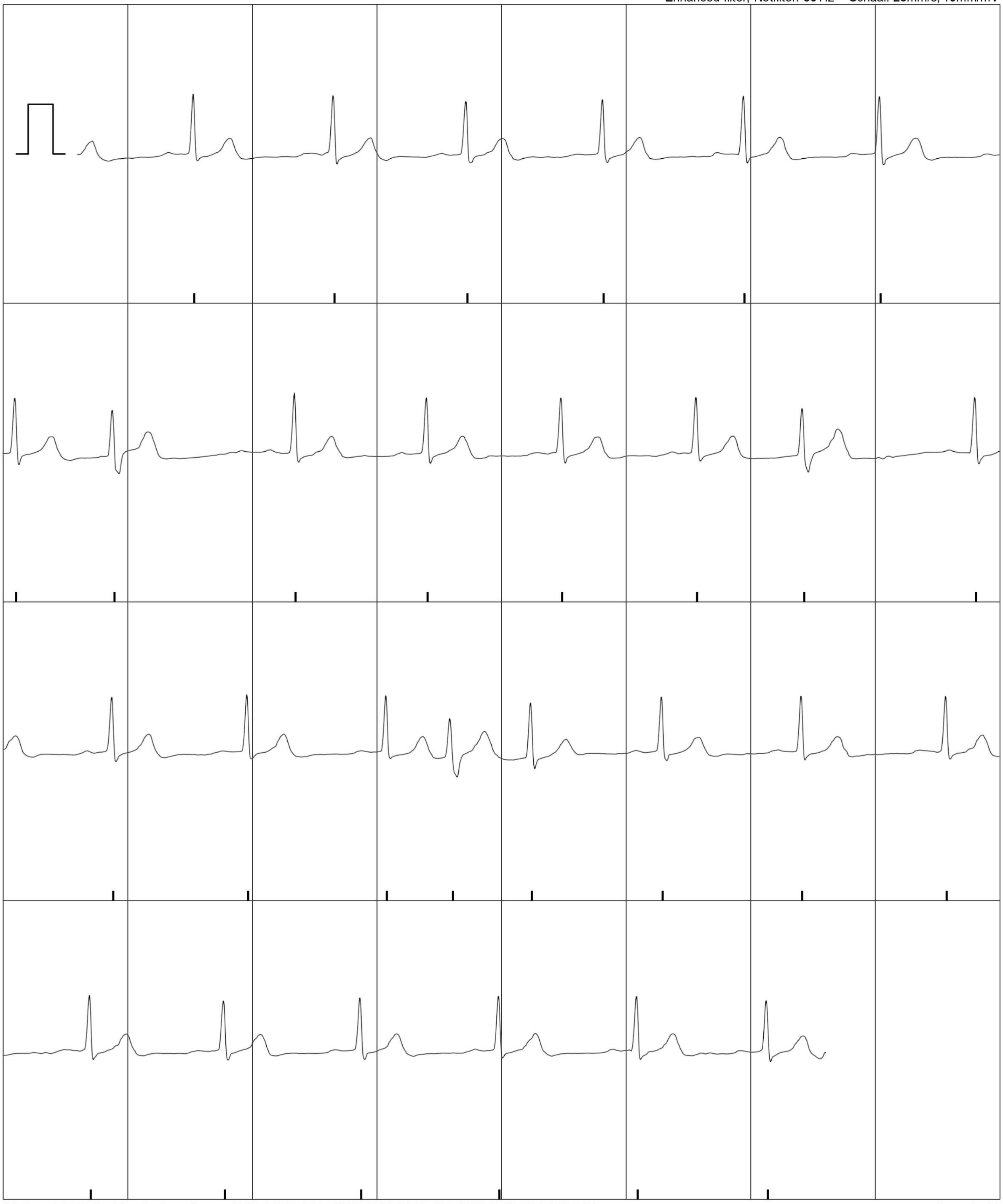

(c) Copyright 2013, AliveCor Inc. aliveecg 5.7.4-06029d739, UUID: b5761587-c2b6-4069-9b70-8f24cb6fe815

INTERPRET-AF_Single-lead_ECG

* 26. How would you classify the following measurement?

- Regular rhythm
- One or more ectopic/missed heartbeats
- Atrial flutter
- Atrial fibrillation
- Unreadable
- Other (please specify)

Enhanced filter, Netfilter: 50 Hz   Schaal: 25mm/s, 10mm/mV

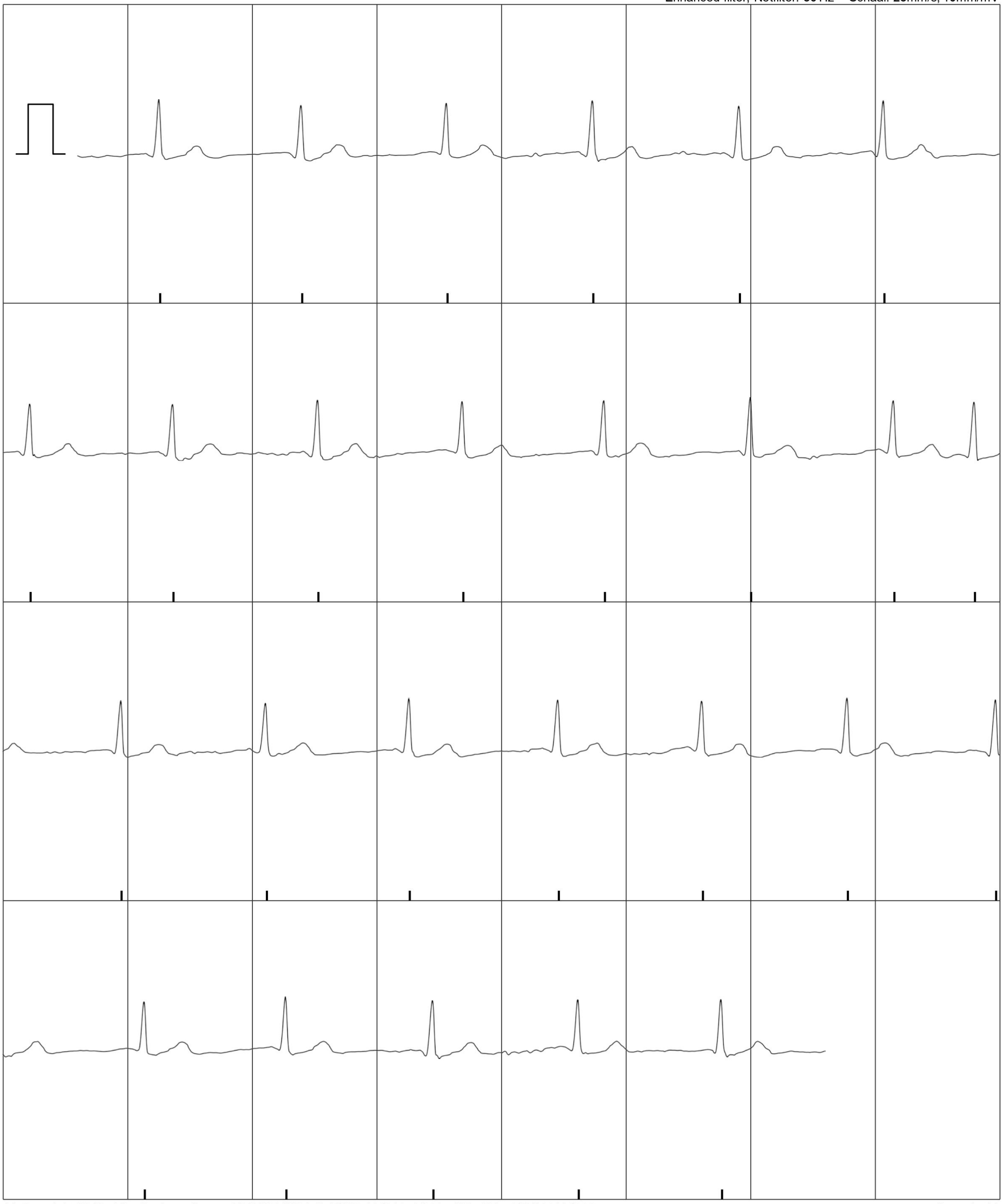

(c) Copyright 2013, AliveCor Inc. aliveecg 5.7.5-b7df46da5, UUID: 14d812a4-bf7e-409a-97ec-70518314d338

INTERPRET-AF_Single-lead_ECG

* 27. How would you classify the following measurement?

- ◯ Regular rhythm
- ◯ One or more ectopic/missed heartbeats
- ◯ Atrial flutter
- ◯ Atrial fibrillation
- ◯ Unreadable
- ◯ Other (please specify)

Schaal: 25mm/s, 10mm/mV

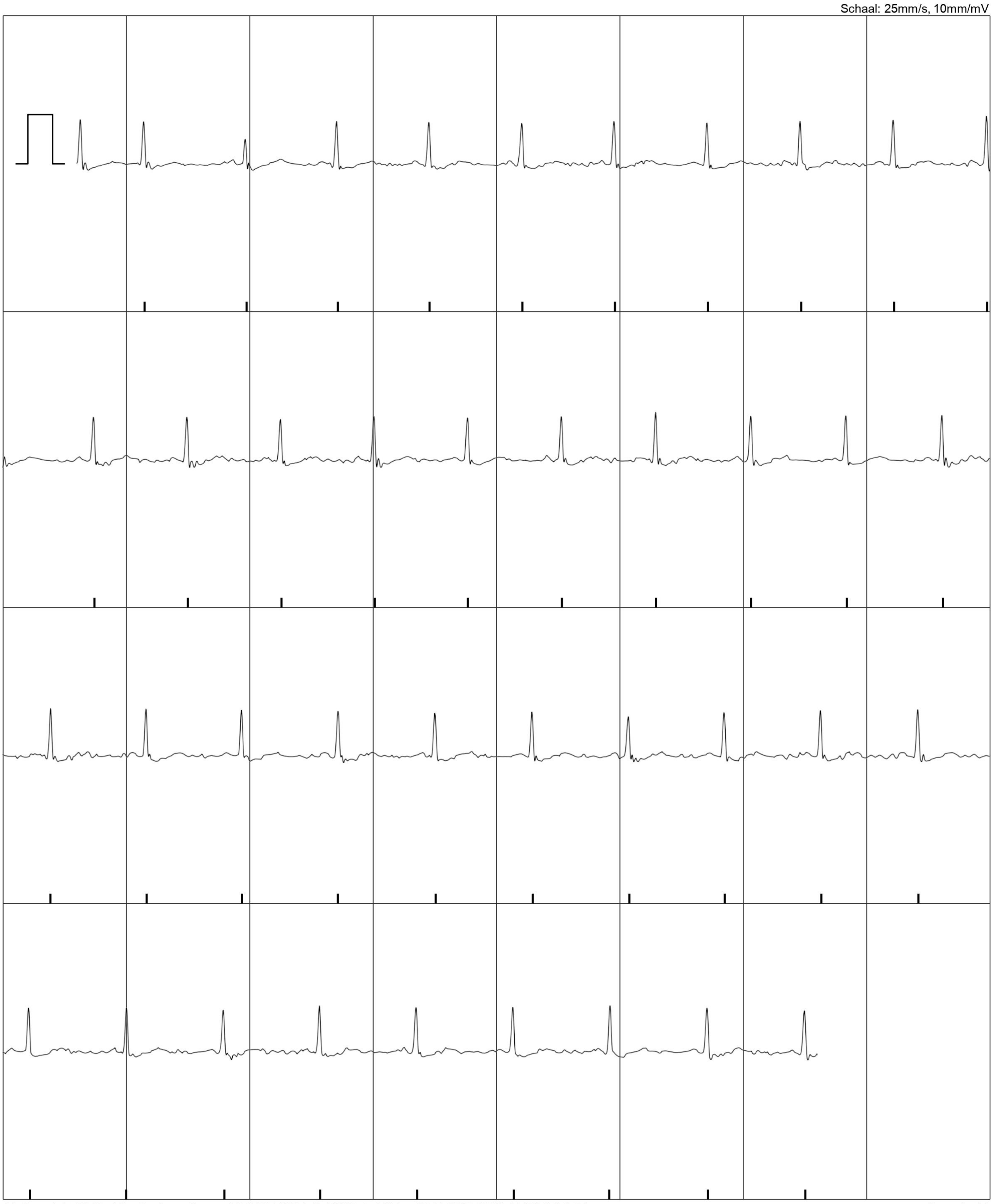

(c) Copyright 2013, AliveCor Inc. aliveecg 5.7.5-b7df46da5, UUID: 3e0f17ae-46c5-4143-8705-4449b974dc81

INTERPRET-AF_Single-lead_ECG

* 28. How would you classify the following measurement?

- ◯ Regular rhythm
- ◯ One or more ectopic/missed heartbeats
- ◯ Atrial flutter
- ◯ Atrial fibrillation
- ◯ Unreadable
- ◯ Other (please specify)

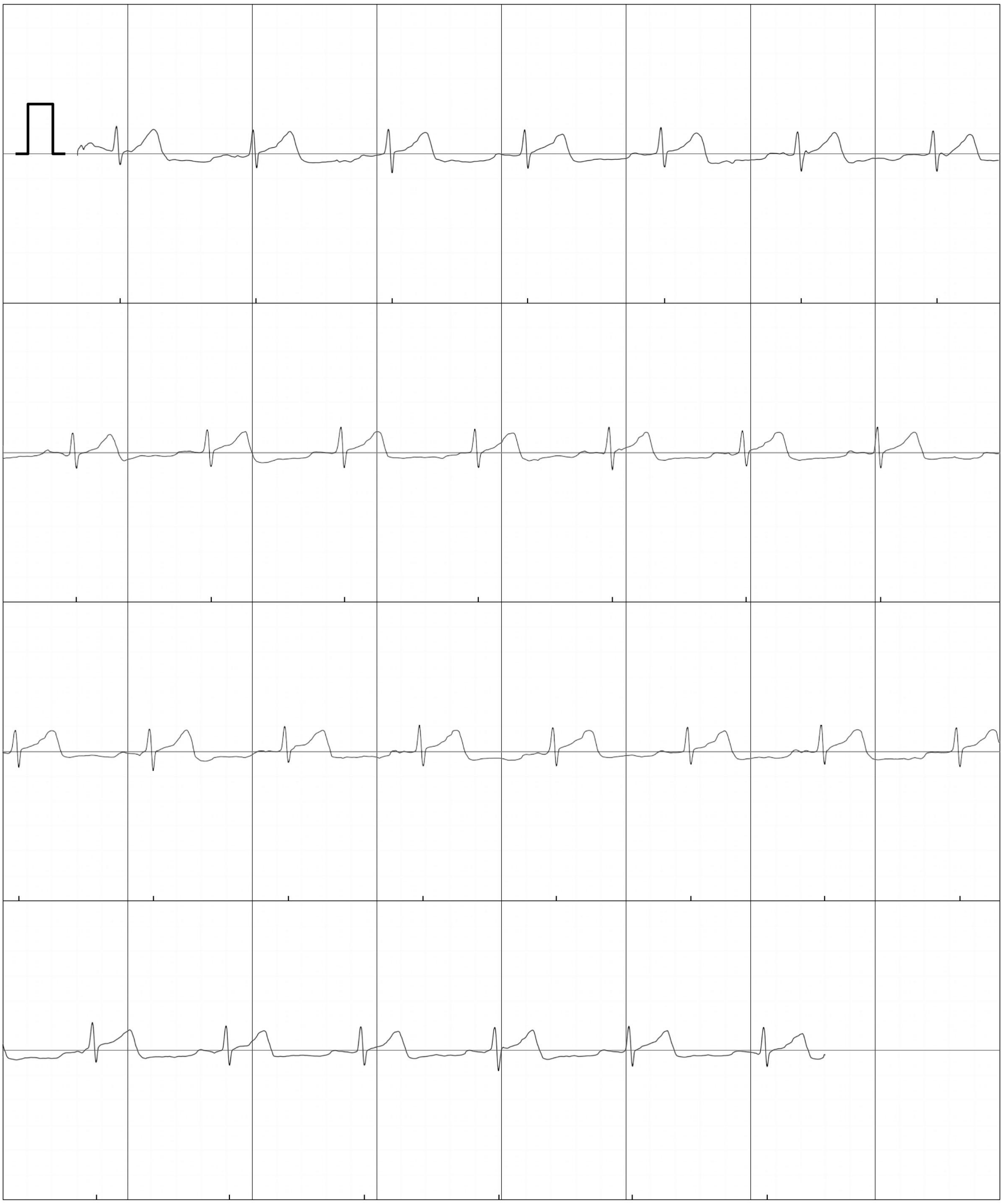

(c) Copyright 2012-2018, AliveCor Inc, Kardia v5.3.0.1584, Report v5.3.0, UUID: 96C7437D-3077-497C-A767-87A4AA2A2619

INTERPRET-AF_Single-lead_ECG

* 29. How would you classify the following measurement?

- Regular rhythm
- One or more ectopic/missed heartbeats
- Atrial flutter
- Atrial fibrillation
- Unreadable
- Other (please specify)

Verbeterd filter, Netfilter: 50Hz   Scale: 25mm/s, 10mm/mV

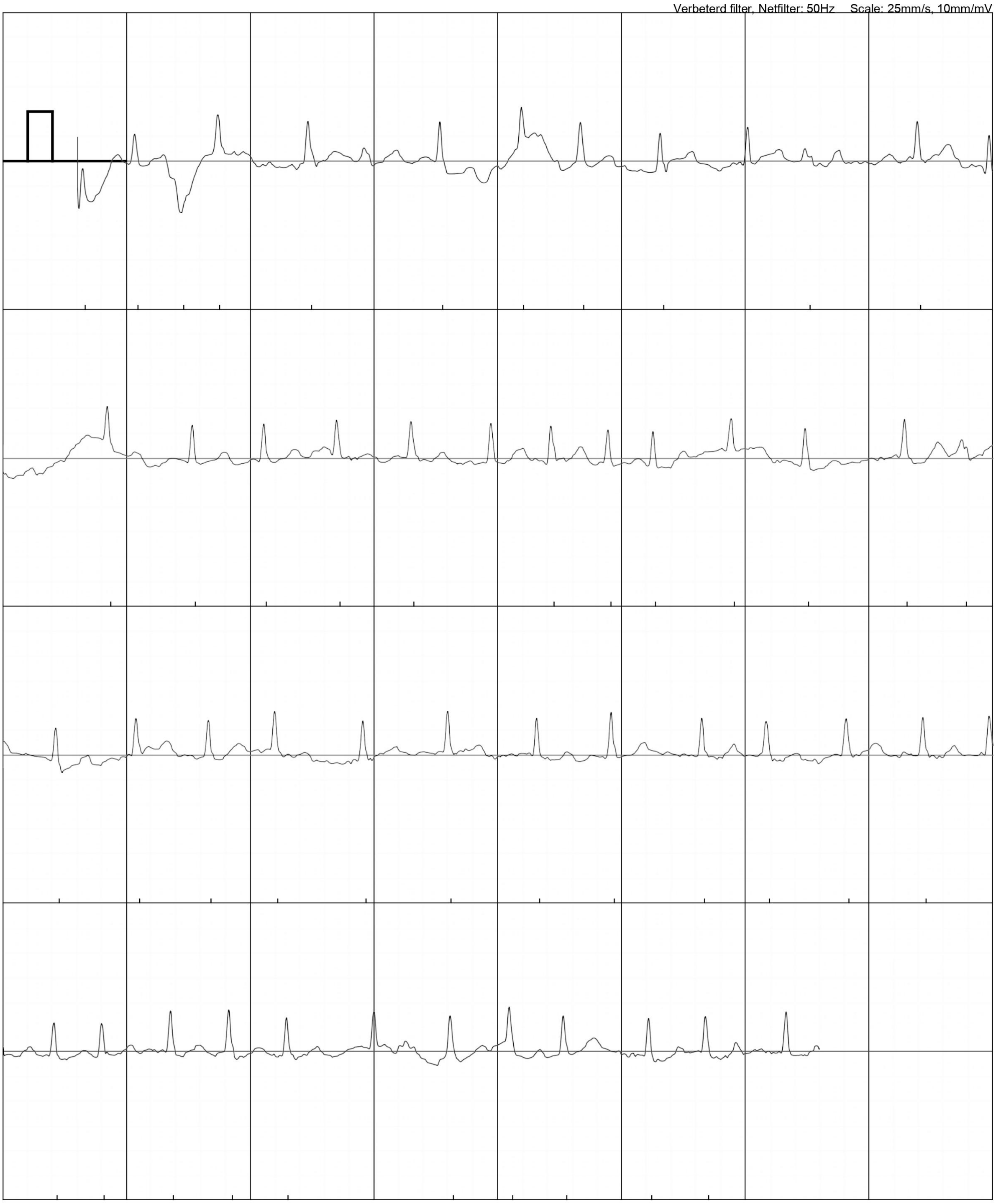

(c) Copyright 2012-2018, AliveCor Inc, Kardia v5.3.0.1584, Report v5.3.0, UUID: 39C3A76F-BC19-4B6E-AFC4-07892EBC4FF1

INTERPRET-AF_Single-lead_ECG

* 30. How would you classify the following measurement?

- ◯ Regular rhythm
- ◯ One or more ectopic/missed heartbeats
- ◯ Atrial flutter
- ◯ Atrial fibrillation
- ◯ Unreadable
- ◯ Other (please specify)

Enhanced filter, Netfilter: 50 Hz   Schaal: 25mm/s, 10mm/mV

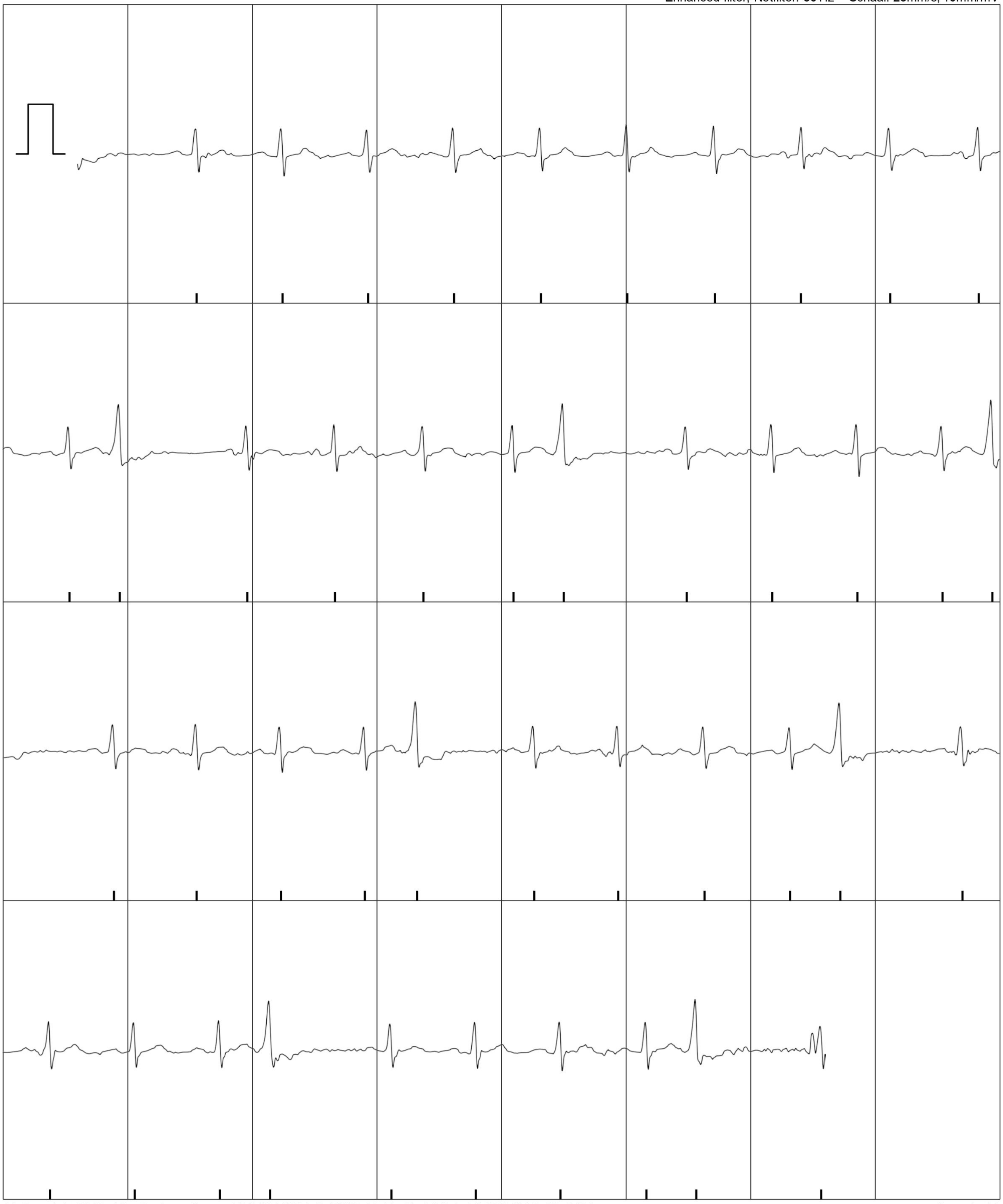

(c) Copyright 2013, AliveCor Inc. aliveecg 5.7.5-b7df46da5, UUID: 06e7cc2b-4653-4e95-86f9-5572c87c9424

INTERPRET-AF_Single-lead_ECG

* 31. How would you classify the following measurement?

- ◯ Regular rhythm
- ◯ One or more ectopic/missed heartbeats
- ◯ Atrial flutter
- ◯ Atrial fibrillation
- ◯ Unreadable
- ◯ Other (please specify)

Verbeterd filter, Netfilter: 50Hz Scale: 25mm/s, 10mm/mV

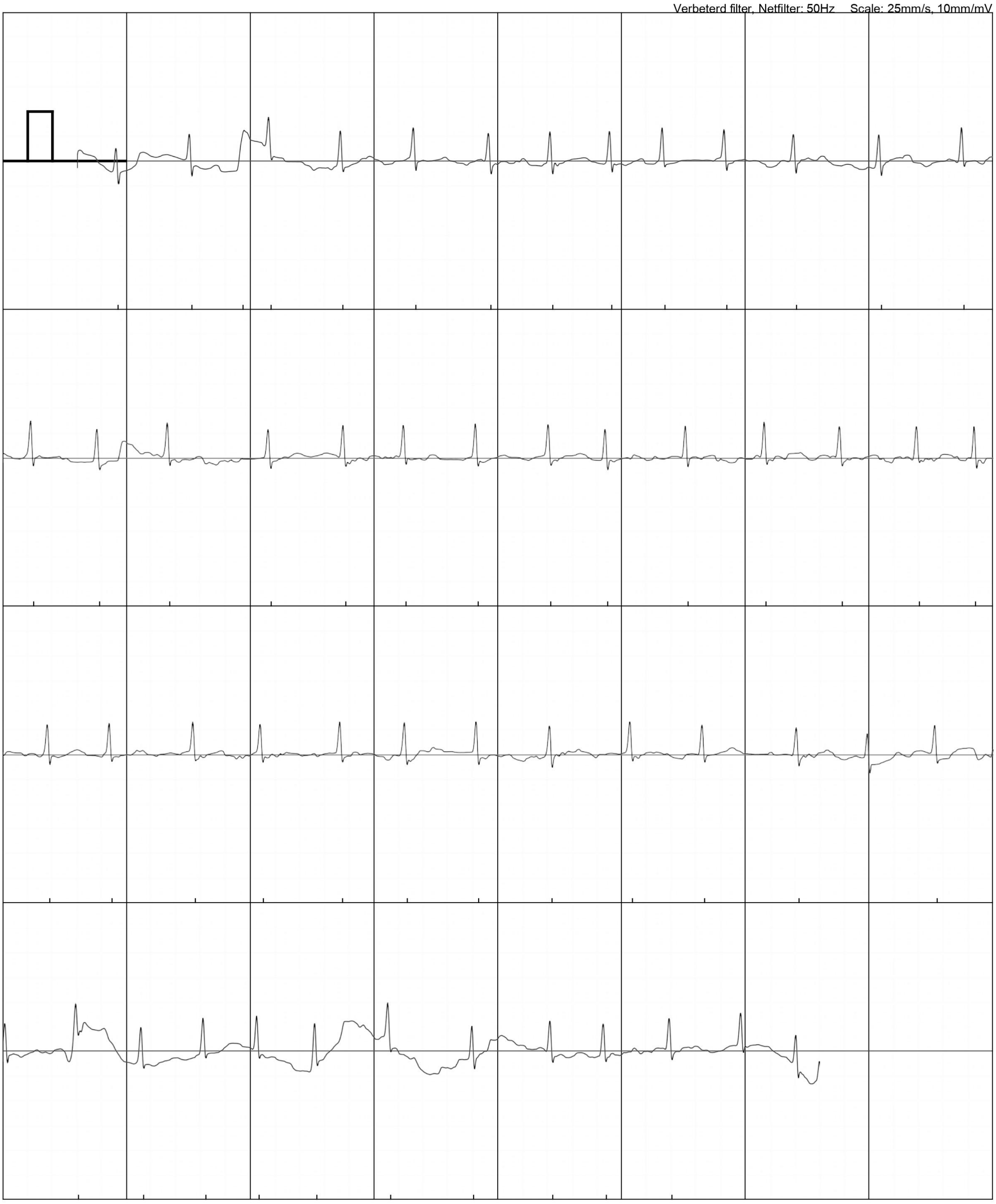

(c) Copyright 2012-2018, AliveCor Inc, Kardia v5.3.0.1584, Report v5.3.0, UUID: 4CC56A46-3B2C-47CC-BBF8-6C915D70F447
