# Supplementary material for: Accuracy of Physicians Interpreting Photoplethysmography and Electrocardiography Tracings to Detect Atrial Fibrillation: INTERPRET-AF
Source: Front Cardiovasc Med. 2021 Sep 20;8:734737. doi: 10.3389/fcvm.2021.734737 (PMC8488290; doi:10.3389/fcvm.2021.734737)

INTERPRET-AF I_PPG

* 9. How would you classify the following measurement?

- Regular rhythm
- One or more ectopic/missed heartbeats
- Atrial flutter
- Atrial fibrillation
- Unreadable
- Other (please specify)

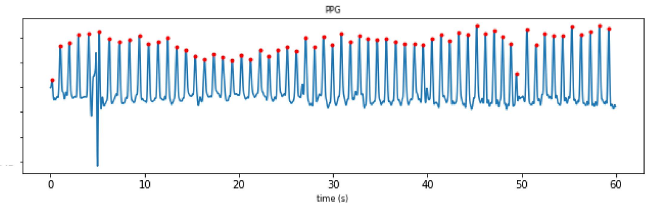

Red dot: potential heartbeat detected by the algorithm

INTERPRET-AF I_PPG

* 10. How would you classify this measurement?

- Regular rhythm
- One or more ectopic/missed heartbeats
- Atrial flutter
- Atrial fibrillation
- Unreadable
- Other (please specify)

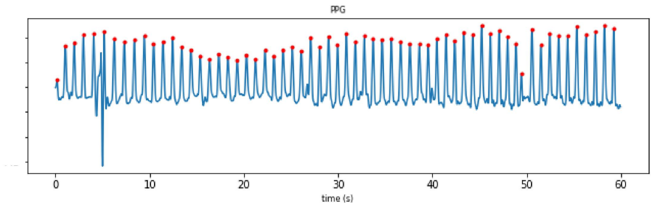

Red dot: potential heartbeat detected by the algorithm
Tachogram: time between the detected peaks (RR-interval)
Poincaré: duration of each RR-interval (RR n) in function of its preceding RR-interval (RR n-1)

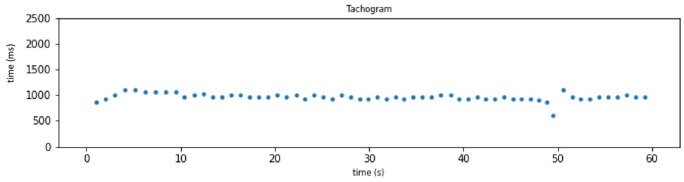

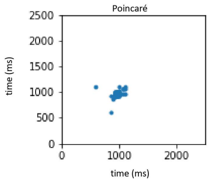

INTERPRET-AF I_PPG

* 11. How would you classify the following measurement?

- ◯ Regular rhythm
- ◯ One or more ectopic/missed heartbeats
- ◯ Atrial flutter
- ◯ Atrial fibrillation
- ◯ Unreadable
- ◯ Other (please specify)

Algorithm output: normal (62 beats per minute)

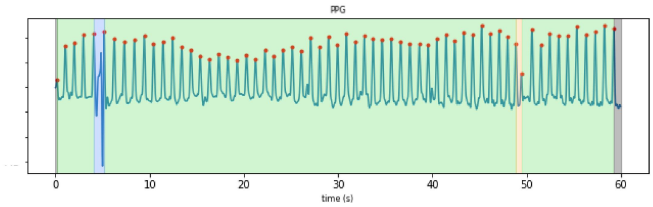

Red dot: potential heartbeat detected by the algorithm
Tachogram: time between the detected peaks (RR-interval)
Poincaré: duration of each RR-interval (RR n) in function of its preceding RR-interval (RR n-1)
Green colour: regular rhythm
Orange colour: one or more ectopic heartbeats
Red colour: atrial fibrillation
Blue: insufficient signal quality

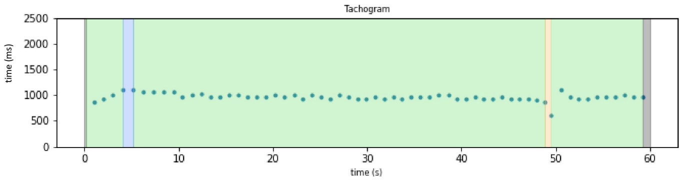

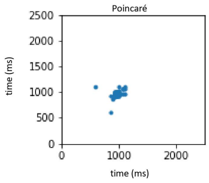

INTERPRET-AF I_PPG

* 12. How would you classify the following measurement?

- Regular rhythm
- One or more ectopic/missed heartbeats
- Atrial flutter
- Atrial fibrillation
- Unreadable
- Other (please specify)

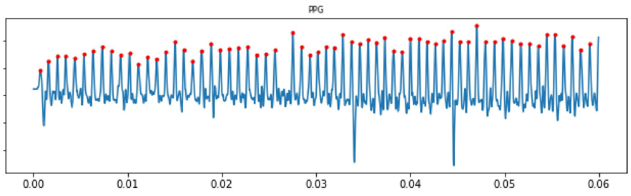

Red dot: potential heartbeat detected by the algorithm

INTERPRET-AF I_PPG

* 13. How would you classify the following measurement?

- Regular rhythm
- One or more ectopic/missed heartbeats
- Atrial flutter
- Atrial fibrillation
- Unreadable
- Other (please specify)

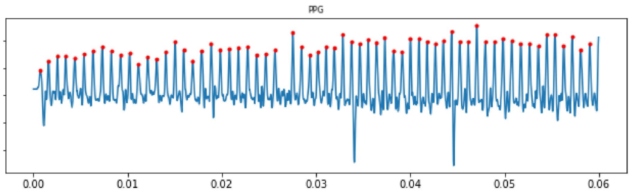

Red dot: potential heartbeat detected by the algorithm
Tachogram: time between the detected peaks (RR-interval)
Poincaré: duration of each RR-interval (RR n) in function of its preceding RR-interval (RR n-1)

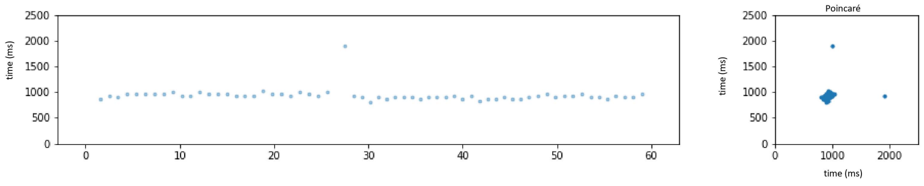

INTERPRET-AF I_PPG

* 14. How would you classify the following measurement?

- Regular rhythm
- One or more ectopic/missed heartbeats
- Atrial flutter
- Atrial fibrillation
- Unreadable
- Other (please specify)

Algorithm output: normal (55 beats per minute)

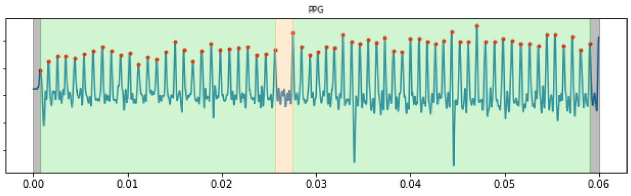

Red dot: potential heartbeat detected by the algorithm
Tachogram: time between the detected peaks (RR-interval)
Poincaré: duration of each RR-interval (RR n) in function of its preceding RR-interval (RR n-1)
Green colour: regular rhythm
Orange colour: one or more ectopic heartbeats
Red colour: atrial fibrillation
Blue: insufficient signal quality

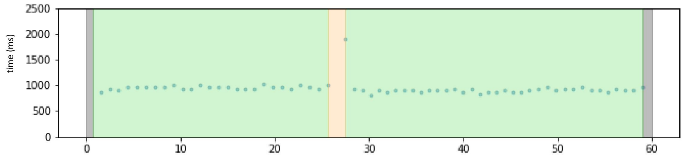

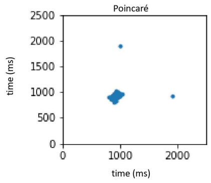

INTERPRET-AF I_PPG

* 15. How would you classify the following measurement?

- Regular rhythm
- One or more ectopic/missed heartbeats
- Atrial flutter
- Atrial fibrillation
- Unreadable
- Other (please specify)

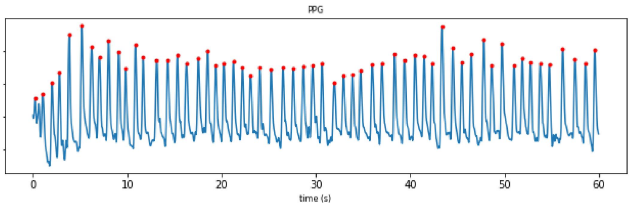

Red dot: potential heartbeat detected by the algorithm

INTERPRET-AF I_PPG

* 16. How would you classify the following measurement?

- ◯ Regular rhythm
- ◯ One or more ectopic/missed heartbeats
- ◯ Atrial flutter
- ◯ Atrial fibrillation
- ◯ Unreadable
- ◯ Other (please specify)

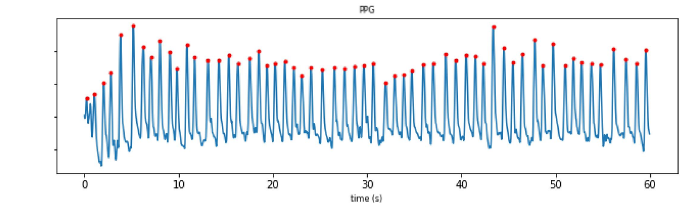

Red dot: potential heartbeat detected by the algorithm
Tachogram: time between the detected peaks (RR-interval)
Poincaré: duration of each RR-interval (RR n) in function of its preceding RR-interval (RR n-1)

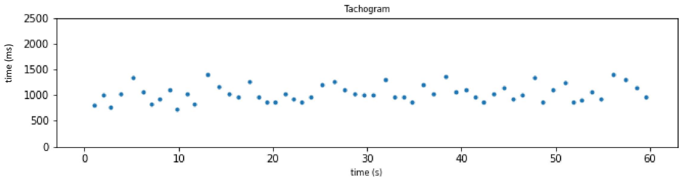

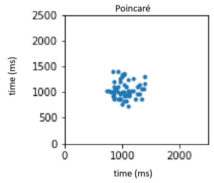

INTERPRET-AF I_PPG

* 17. How would you classify the following measurement?

- ◯ Regular rhythm
- ◯ One or more ectopic/missed heartbeats
- ◯ Atrial flutter
- ◯ Atrial fibrillation
- ◯ Unreadable
- ◯ Other (please specify)

Algorithm output: urgent (58 beats per minute)

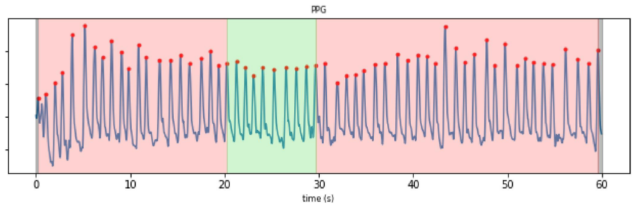

Red dot: potential heartbeat detected by the algorithm
Tachogram: time between the detected peaks (RR-interval)
Poincaré: duration of each RR-interval (RR n) in function of its preceding RR-interval (RR n-1)
Green colour: regular rhythm
Orange colour: one or more ectopic heartbeats
Red colour: atrial fibrillation
Blue: insufficient signal quality

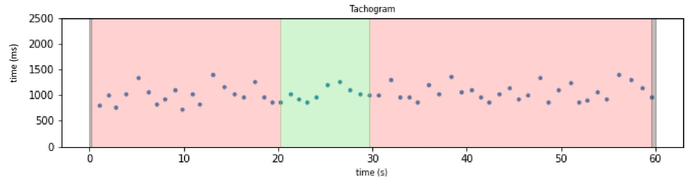

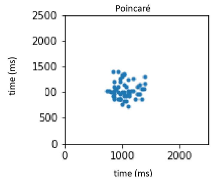

INTERPRET-AF I_PPG

* 18. How would you classify the following measurement?

- Regular rhythm
- One or more ectopic/missed heartbeats
- Atrial flutter
- Atrial fibrillation
- Unreadable
- Other (please specify)

Red dot: potential heartbeat detected by the algorithm

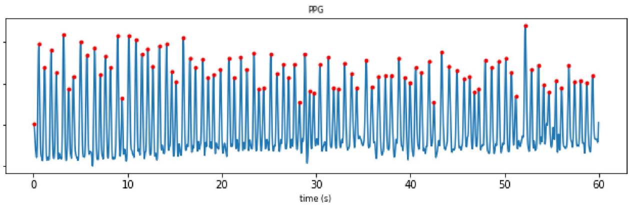

INTERPRET-AF I_PPG

* 19. How would you classify the following measurement?

- Regular rhythm
- One or more ectopic/missed heartbeats
- Atrial flutter
- Atrial fibrillation
- Unreadable
- Other (please specify)

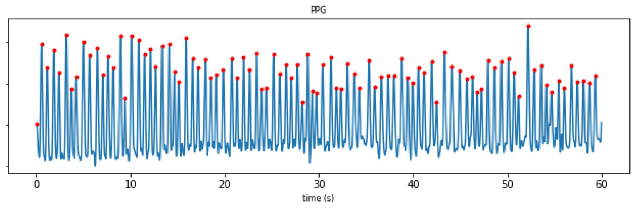

Red dot: potential heartbeat detected by the algorithm
Tachogram: time between the detected peaks (RR-interval)
Poincaré: duration of each RR-interval (RR n) in function of its preceding RR-interval (RR n-1)

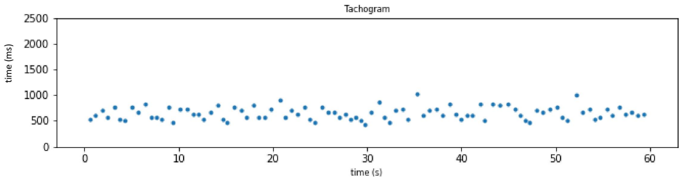

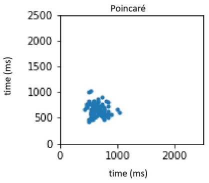

INTERPRET-AF I_PPG

* 20. How would you classify the following measurement?

- ◯ Regular rhythm
- ◯ One or more ectopic/missed heartbeats
- ◯ Atrial flutter
- ◯ Atrial fibrillation
- ◯ Unreadable
- ◯ Other (please specify)

Algorithm output: urgent (58 beats per minute)

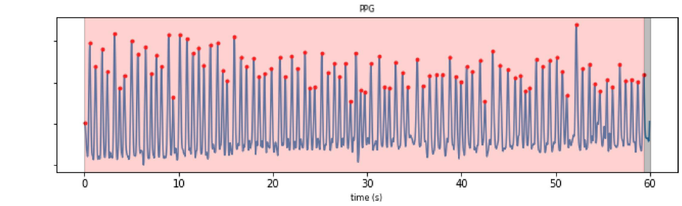

Red dot: potential heartbeat detected by the algorithm
Tachogram: time between the detected peaks (RR-interval)
Poincaré: duration of each RR-interval (RR n) in function of its preceding RR-interval (RR n-1)
Green colour: regular rhythm
Orange colour: one or more ectopic heartbeats
Red colour: atrial fibrillation
Blue: insufficient signal quality

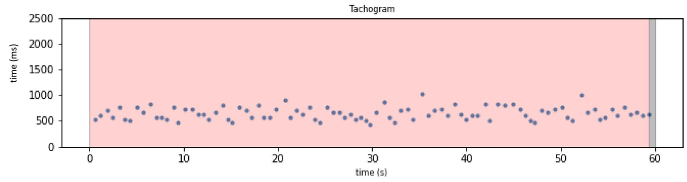

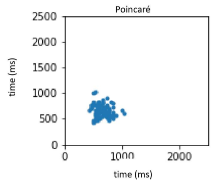

INTERPRET-AF I_PPG

* 21. How would you classify the following measurement?

- Regular rhythm
- One or more ectopic/missed heartbeats
- Atrial flutter
- Atrial fibrillation
- Unreadable
- Other (please specify)

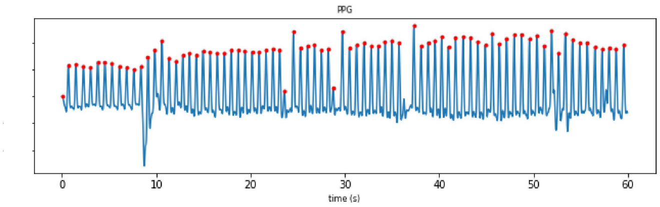

Red dot: potential heartbeat detected by the algorithm

INTERPRET-AF I_PPG

* 22. How would you classify the following measurement?

- ◯ Regular rhythm
- ◯ One or more ectopic/missed heartbeats
- ◯ Atrial flutter
- ◯ Atrial fibrillation
- ◯ Unreadable
- ◯ Other (please specify)

Red dot: potential heartbeat detected by the algorithm
Tachogram: time between the detected peaks (RR-interval)
Poincaré: duration of each RR-interval (RR n) in function of its preceding RR-interval (RR n-1)

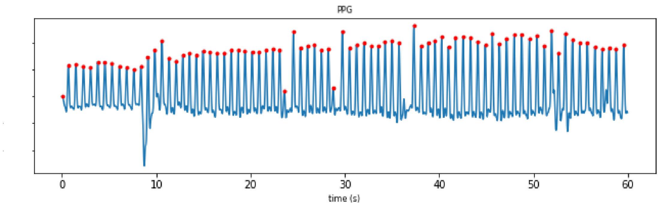

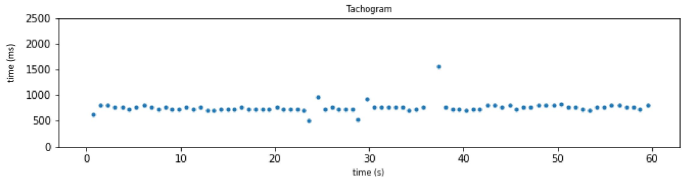

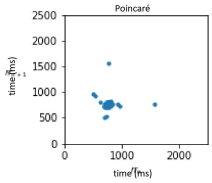

INTERPRET-AF I_PPG

* 23. How would you classify the following measurement?

- ◯ Regular rhythm
- ◯ One or more ectopic/missed heartbeats
- ◯ Atrial flutter
- ◯ Atrial fibrillation
- ◯ Unreadable
- ◯ Other (please specify)

Algorithm output: warning (79 beats per minute)

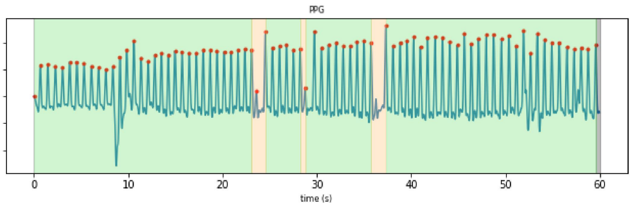

Red dot: potential heartbeat detected by the algorithm
Tachogram: time between the detected peaks (RR-interval)
Poincaré: duration of each RR-interval (RR n) in function of its preceding RR-interval (RR n-1)
Green colour: regular rhythm
Orange colour: one or more ectopic heartbeats
Red colour: atrial fibrillation
Blue: insufficient signal quality

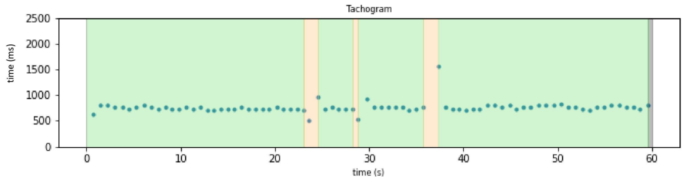

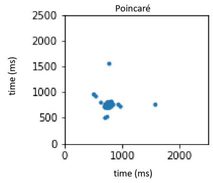

INTERPRET-AF I_PPG

* 24. How would you classify the following measurement?

- Regular rhythm
- One or more ectopic/missed heartbeats
- Atrial flutter
- Atrial fibrillation
- Unreadable
- Other (please specify)

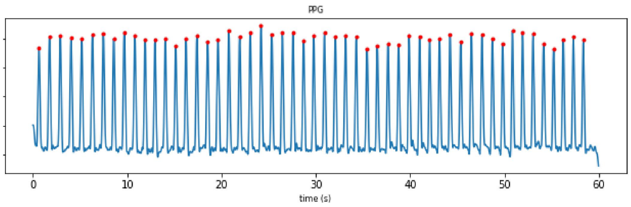

Red dot: potential heartbeat detected by the algorithm

INTERPRET-AF I_PPG

* 25. How would you classify the following measurement?

- ◯ Regular rhythm
- ◯ One or more ectpic/missed heartbeats
- ◯ Atrial flutter
- ◯ Atrial fibrillation
- ◯ Unreadable
- ◯ Other (please specify)

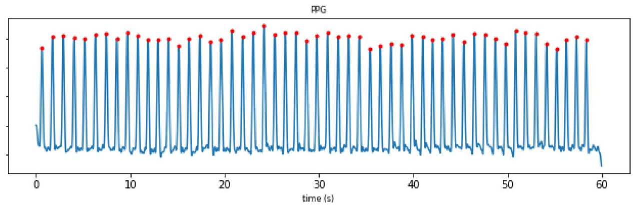

Red dot: potential heartbeat detected by the algorithm
Tachogram: time between the detected peaks (RR-interval)
Poincaré: duration of each RR-interval (RR n) in function of its preceding RR-interval (RR n-1)

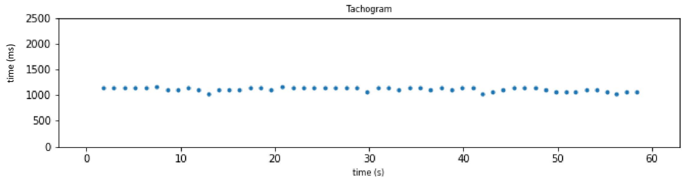

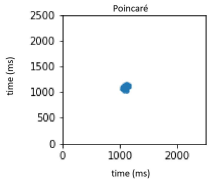

INTERPRET-AF I_PPG

* 26. How would you classify the following measurement?

- ◯ Regular rhythm
- ◯ One or more ectopic/missed heartbeats
- ◯ Atrial flutter
- ◯ Atrial fibrillation
- ◯ Unreadable
- ◯ Other (please specify)

Algorithm output: normal (54 beats per minute)

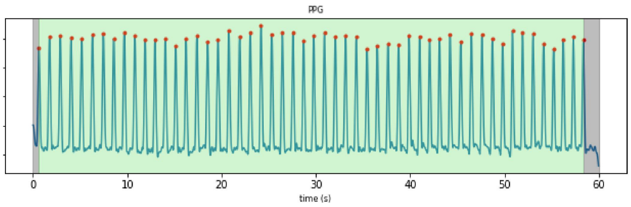

Red dot: potential heartbeat detected by the algorithm
Tachogram: time between the detected peaks (RR-interval)
Poincaré: duration of each RR-interval (RR n) in function of its preceding RR-interval (RR n-1)
Green colour: regular rhythm
Orange colour: one or more ectopic heartbeats
Red colour: atrial fibrillation
Blue: insufficient signal quality

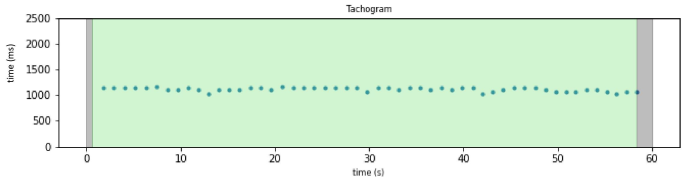

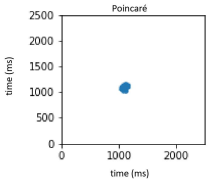

INTERPRET-AF I_PPG

* 27. How would you classify the following measurement?

- Regular rhythm
- One or more ectopic/missed heartbeats
- Atrial flutter
- Atrial fibrillation
- Unreadable
- Other (please specify)

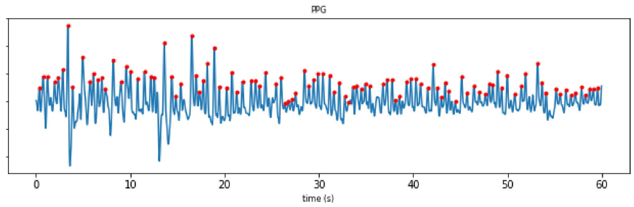

Red dot: potential heartbeat detected by the algorithm

INTERPRET-AF I_PPG

* 28. How would you classify the following measurement?

- Regular rhythm
- One or more ectopic/missed heartbeats
- Atrial flutter
- Atrial fibrillation
- Unreadable
- Other (please specify)

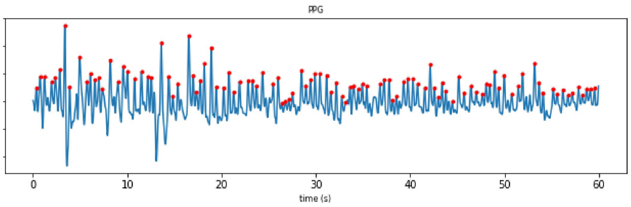

Red dot: potential heartbeat detected by the algorithm
Tachogram: time between the detected peaks (RR-interval)
Poincaré: duration of each RR-interval (RR n) in function of its preceding RR-interval (RR n-1)

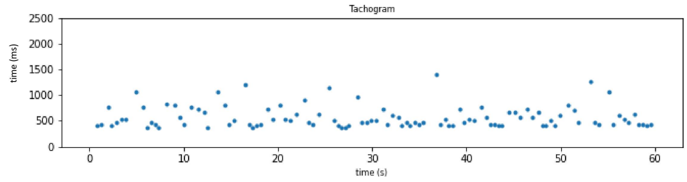

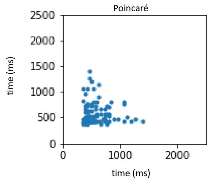

INTERPRET-AF I_PPG

* 29. How would you classify the following measurement?

- ◯ Regular rhythm
- ◯ One or more ectopic/missed heartbeats
- ◯ Atrial flutter
- ◯ Atrial fibrillation
- ◯ Unreadable
- ◯ Other (please specify)

Algorithm output: urgent (105 beats per minute)

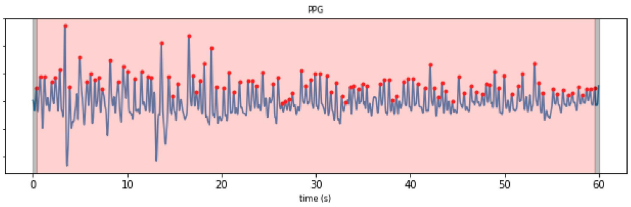

Red dot: potential heartbeat detected by the algorithm
Tachogram: time between the detected peaks (RR-interval)
Poincaré: duration of each RR-interval (RR n) in function of its preceding RR-interval (RR n-1)
Green colour: regular rhythm
Orange colour: one or more ectopic heartbeats
Red colour: atrial fibrillation
Blue: insufficient signal quality

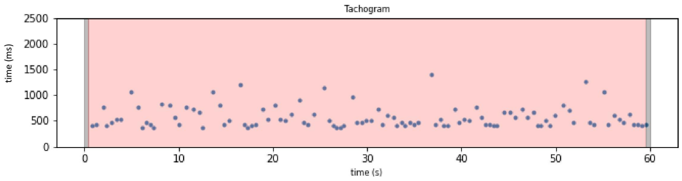

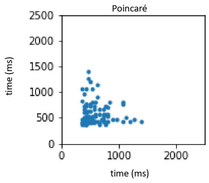

INTERPRET-AF I_PPG

* 30. How would you classify the following measurement?

- Regular rhythm
- One or more ectopic/missed heartbeats
- Atrial flutter
- Atrial fibrillation
- Unreadable
- Other (please specify)

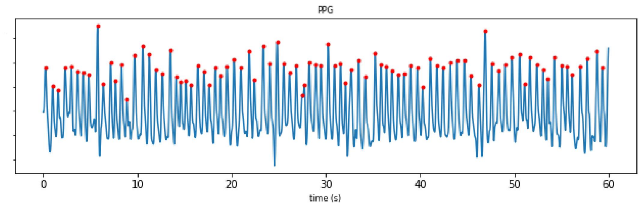

Red dot: potential heartbeat detected by the algorithm

INTERPRET-AF I_PPG

* 31. How would you classify the following measurement?

- Regular rhythm
- One or more ectopic/missed heartbeats
- Atrial flutter
- Atrial fibrillation
- Unreadable
- Other (please specify)

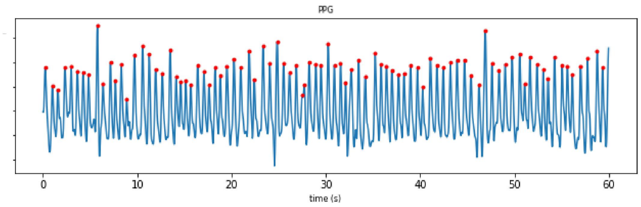

Red dot: potential heartbeat detected by the algorithm
Tachogram: time between the detected peaks (RR-interval)
Poincaré: duration of each RR-interval (RR n) in function of its preceding RR-interval (RR n-1)

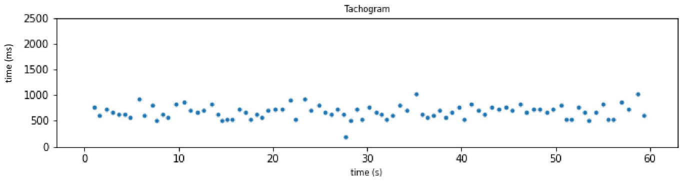

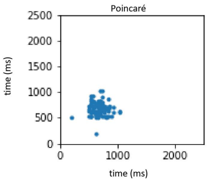

INTERPRET-AF I_PPG

* 32. How would you classify the following measurement?

- Regular rhythm
- One or more ectopic/missed heartbeats
- Atrial flutter
- Atrial fibrillation
- Unreadable
- Other (please specify)

Algorithm output: urgent (88 beats per minute)

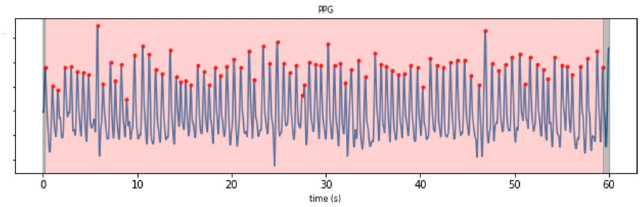

Red dot: potential heartbeat detected by the algorithm
Tachogram: time between the detected peaks (RR-interval)
Poincaré: duration of each RR-interval (RR n) in function of its preceding RR-interval (RR n-1)
Green colour: regular rhythm
Orange colour: one or more ectopic heartbeats
Red colour: atrial fibrillation
Blue: insufficient signal quality

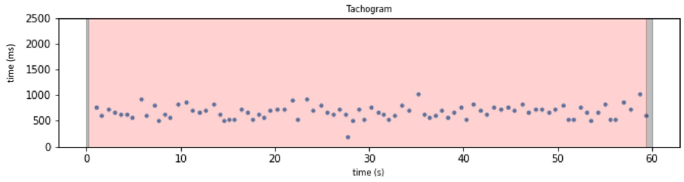

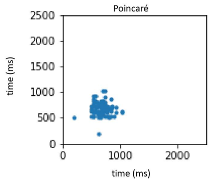

INTERPRET-AF I_PPG

* 33. How would you classify the following measurement?

- Regular rhythm
- One or more ectopic/missed heartbeats
- Atrial flutter
- Atrial fibrillation
- Unreadable
- Other (please specify)

Red dot: potential heartbeat detected by the algorithm

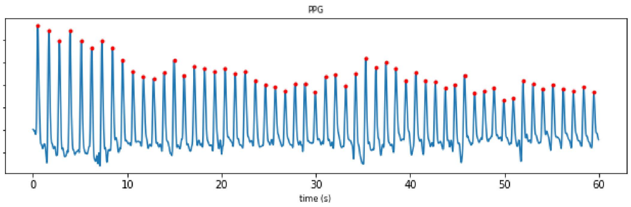

INTERPRET-AF I_PPG

* 34. How would you classify the following measurement?

- Regular rhythm
- One or more ectopic/missed heartbeats
- Atrial flutter
- Atrial fibrillation
- Unreadable
- Other (please specify)

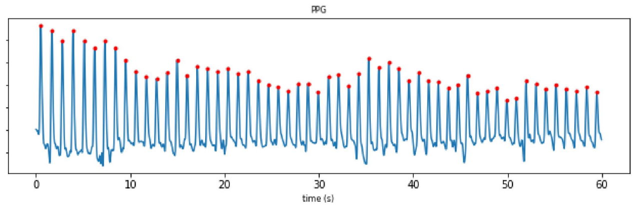

Red dot: potential heartbeat detected by the algorithm
Tachogram: time between the detected peaks (RR-interval)
Poincaré: duration of each RR-interval (RR n) in function of its preceding RR-interval (RR n-1)

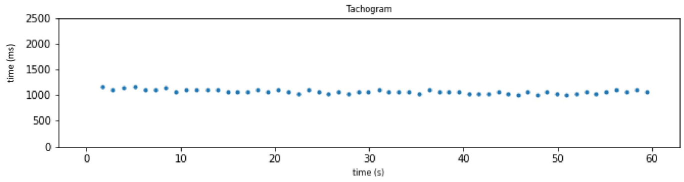

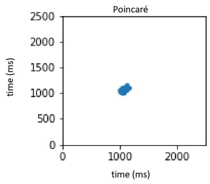

INTERPRET-AF I_PPG

* 35. How would you classify the following measurement?

- Regular rhythm
- One or more ectopic/missed heartbeats
- Atrial flutter
- Atrial fibrillation
- Unreadable
- Other (please specify)

Algorithm output: normal (56 beats per minute)

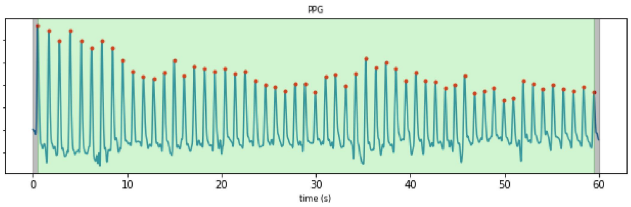

Red dot: potential heartbeat detected by the algorithm
Tachogram: time between the detected peaks (RR-interval)
Poincaré: duration of each RR-interval (RR n) in function of its preceding RR-interval (RR n-1)
Green colour: regular rhythm
Orange colour: one or more ectopic heartbeats
Red colour: atrial fibrillation
Blue: insufficient signal quality

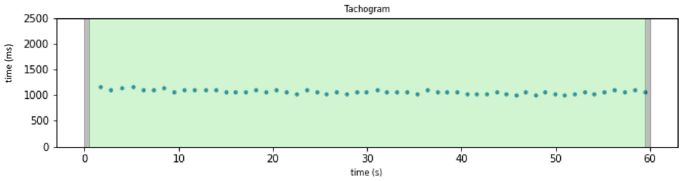

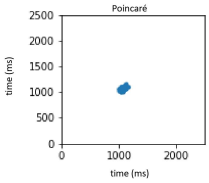

INTERPRET-AF I_PPG

* 36. How would you classify the following measurement?

- Regular rhythm
- One or more ectopic/missed heartbeats
- Atrial flutter
- Atrial fibrillation
- Unreadable
- Other (please specify)

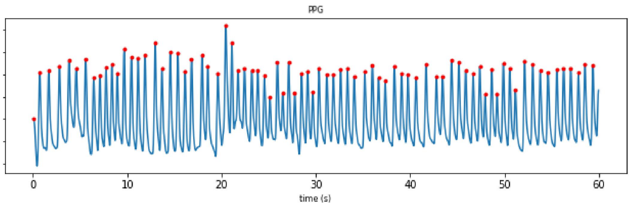

Red dot: potential heartbeat detected by the algorithm

INTERPRET-AF I_PPG

* 37. How would you classify the following measurement?

- Regular rhythm
- One or more ectopic/missed heartbeats
- Atrial flutter
- Atrial fibrillation
- Unreadable
- Other (please specify)

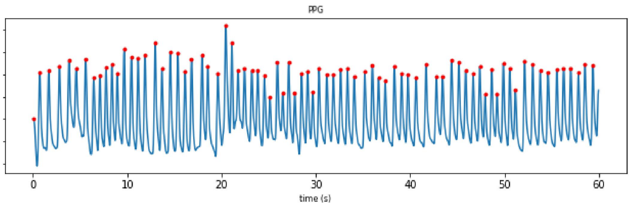

Red dot: potential heartbeat detected by the algorithm
Tachogram: time between the detected peaks (RR-interval)
Poincaré: duration of each RR-interval (RR n) in function of its preceding RR-interval (RR n-1)

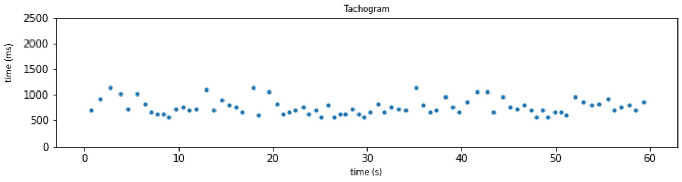

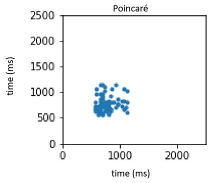

INTERPRET-AF I_PPG

* 38. How would you classify the following measurement?

- Regular rhythm
- One or more ectopic/missed heartbeats
- Atrial flutter
- Atrial fibrillation
- Unreadable
- Other (please specify)

Algorithm output: urgent (78 beats per minute)

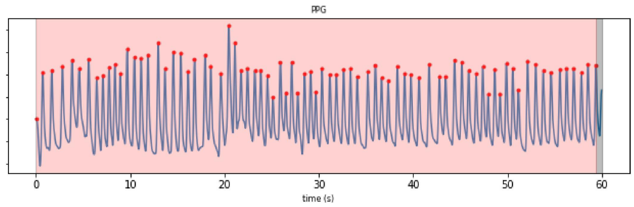

Red dot: potential heartbeat detected by the algorithm
Tachogram: time between the detected peaks (RR-interval)
Poincaré: duration of each RR-interval (RR n) in function of its preceding RR-interval (RR n-1)
Green colour: regular rhythm
Orange colour: one or more ectopic heartbeats
Red colour: atrial fibrillation
Blue: insufficient signal quality

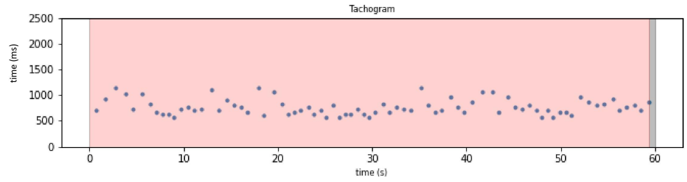

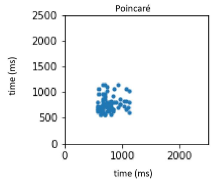

INTERPRET-AF I_PPG

* 39. How would you classify the following measurement?

- Regular rhythm
- One or more ectopic/missed heartbeats
- Atrial flutter
- Atrial fibrillation
- Unreadable
- Other (please specify)

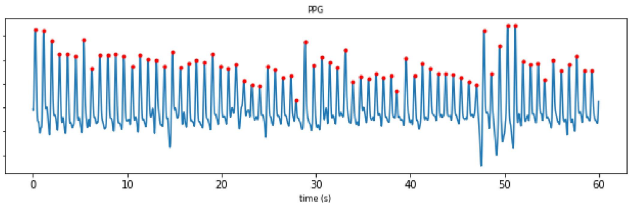

Red dot: potential heartbeat detected by the algorithm

INTERPRET-AF I_PPG

* 40. How would you classify the following measurement?

- Regular rhythm
- One or more ectopic/missed heartbeats
- Atrial flutter
- Atrial fibrillation
- Unreadable
- Other (please specify)

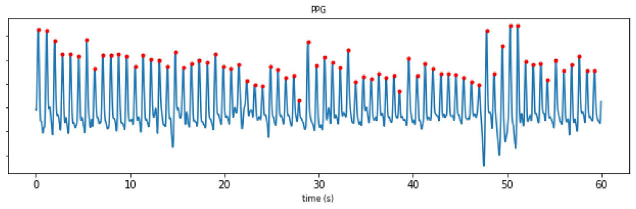

Red dot: potential heartbeat detected by the algorithm
Tachogram: time between the detected peaks (RR-interval)
Poincaré: duration of each RR-interval (RR n) in function of its preceding RR-interval (RR n-1)

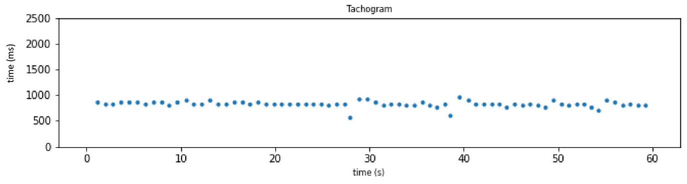

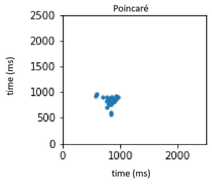

INTERPRET-AF I_PPG

* 41. How would you classify the following measurement?

- ○ Regular rhythm
- ○ One or more ectopic/missed heartbeats
- ○ Atrial flutter
- ○ Atrial fibrillation
- ○ Unreadable
- ○ Other (please specify)

Algorithm output: warning (72 beats per minute)

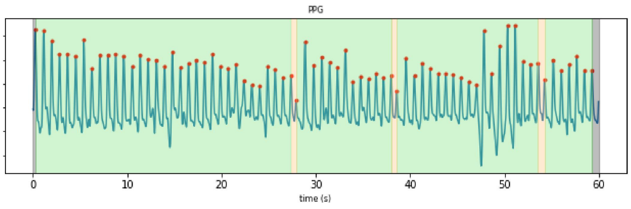

Red dot: potential heartbeat detected by the algorithm
Tachogram: time between the detected peaks (RR-interval)
Poincaré: duration of each RR-interval (RR n) in function of its preceding RR-interval (RR n-1)
Green colour: regular rhythm
Orange colour: one or more ectopic heartbeats
Red colour: atrial fibrillation
Blue: insufficient signal quality

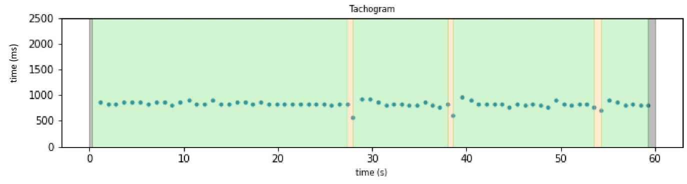

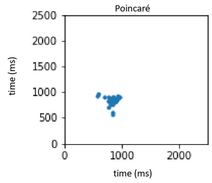

INTERPRET-AF I_PPG

* 42. How would you classify the following measurement?

- Regular rhythm
- One or more ectopic/missed heartbeats
- Atrial flutter
- Atrial fibrillation
- Unreadable
- Other (please specify)

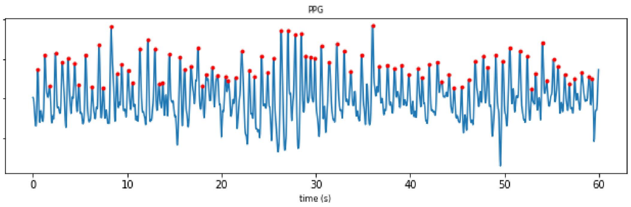

Red dot: potential heartbeat detected by the algorithm

INTERPRET-AF I_PPG

* 43. How would you classify the following measurement?

- Regular rhythm
- One or more ectopic/missed heartbeats
- Atrial flutter
- Atrial fibrillation
- Unreadable
- Other (please specify)

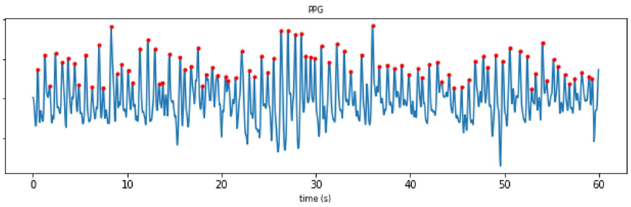

Red dot: potential heartbeat detected by the algorithm
Tachogram: time between the detected peaks (RR-interval)
Poincaré: duration of each RR-interval (RR n) in function of its preceding RR-interval (RR n-1)

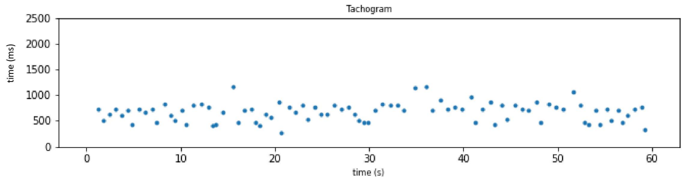

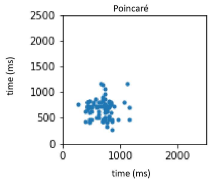

INTERPRET-AF I_PPG

* 44. How would you classify the following measurement?

- Regular rhythm
- One or more ectopic/missed heartbeats
- Atrial flutter
- Atrial fibrillation
- Unreadable
- Other (please specify)

Algorithm output: urgent (89 beats per minute)

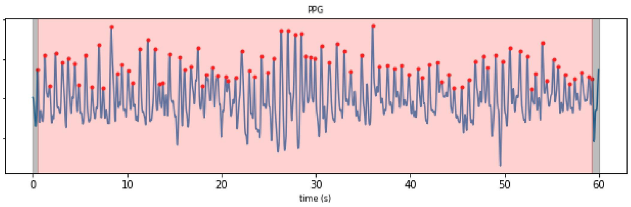

Red dot: potential heartbeat detected by the algorithm
Tachogram: time between the detected peaks (RR-interval)
Poincaré: duration of each RR-interval (RR n) in function of its preceding RR-interval (RR n-1)
Green colour: regular rhythm
Orange colour: one or more ectopic heartbeats
Red colour: atrial fibrillation
Blue: insufficient signal quality

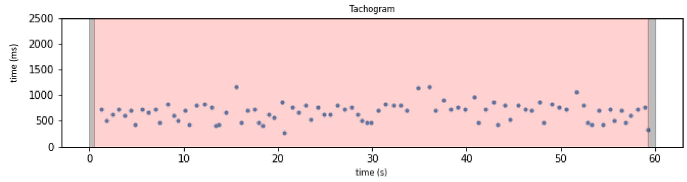

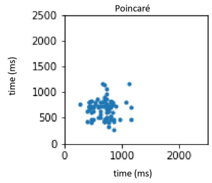

INTERPRET-AF I_PPG

* 45. How would you classify the following measurement?

- Regular rhythm
- One or more ectopic/missed heartbeats
- Atrial flutter
- Atrial fibrillation
- Unreadable
- Other (please specify)

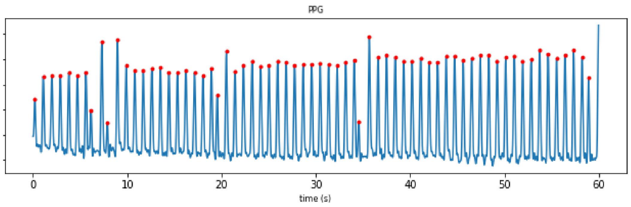

Red dot: potential heartbeat detected by the algorithm

INTERPRET-AF I_PPG

* 46. How would you classify the following measurement?

- Regular rhythm
- One or more ectopic/missed heartbeats
- Atrial flutter
- Atrial fibrillation
- Unreadable
- Other (please specify)

Red dot: potential heartbeat detected by the algorithm
Tachogram: time between the detected peaks (RR-interval)
Poincaré: duration of each RR-interval (RR n) in function of its preceding RR-interval (RR n-1)

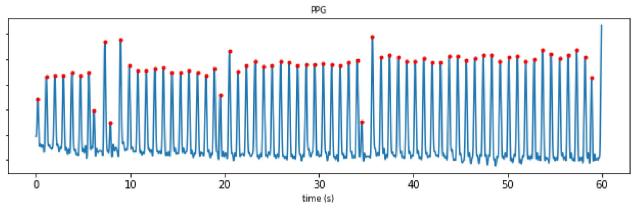

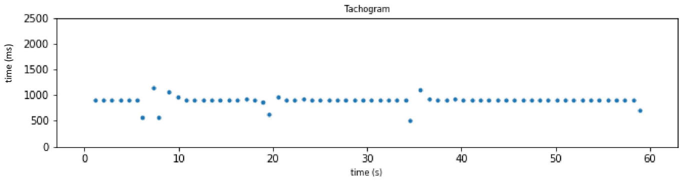

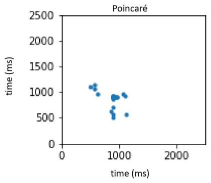

INTERPRET-AF I_PPG

* 47. How would you classify the following measurement?

- ◯ Regular rhythm
- ◯ One or more ectopic/missed heartbeats
- ◯ Atrial flutter
- ◯ Atrial fibrillation
- ◯ Unreadable
- ◯ Other (please specify)

Algorithm output: warning (67 beats per minute)

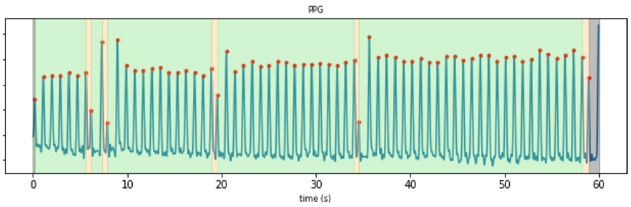

Red dot: potential heartbeat detected by the algorithm
Tachogram: time between the detected peaks (RR-interval)
Poincaré: duration of each RR-interval (RR n) in function of its preceding RR-interval (RR n-1)
Green colour: regular rhythm
Orange colour: one or more ectopic heartbeats
Red colour: atrial fibrillation
Blue: insufficient signal quality

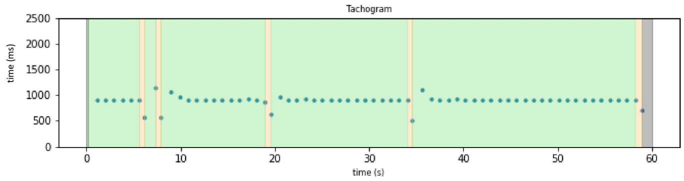

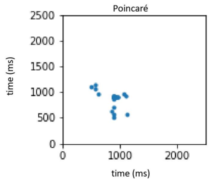

INTERPRET-AF I_PPG

* 48. How would you classify the following measurement?

- Regular rhythm
- One or more ectopic/missed heartbeats
- Atrial flutter
- Atrial fibrillation
- Unreadable
- Other (please specify)

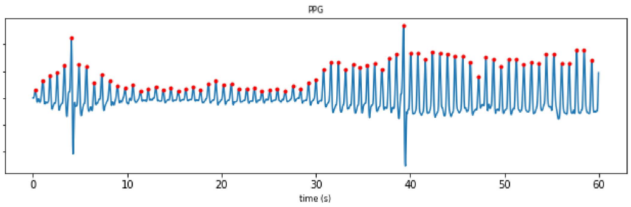

Red dot: potential heartbeat detected by the algorithm

INTERPRET-AF I_PPG

* 49. How would you classify the following measurement?

- Regular rhythm
- One or more ectopic/missed heartbeats
- Atrial flutter
- Atrial fibrillation
- Unreadable
- Other (please specify)

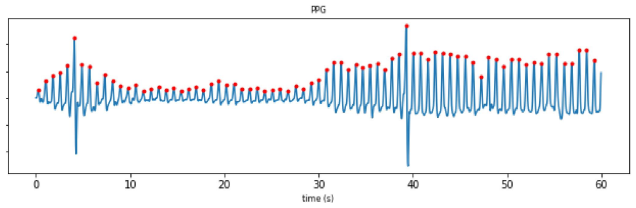

Red dot: potential heartbeat detected by the algorithm
Tachogram: time between the detected peaks (RR-interval)
Poincaré: duration of each RR-interval (RR n) in function of its preceding RR-interval (RR n-1)

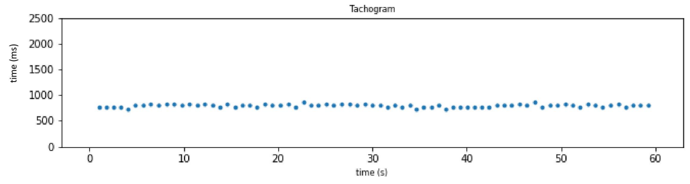

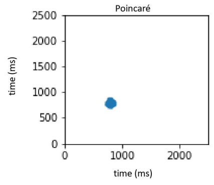

INTERPRET-AF I_PPG

* 50. How would you classify the following measurement?

- Regular rhythm
- One or more ectopic/missed heartbeats
- Atrial flutter
- Atrial fibrillation
- Unreadable
- Other (please specify)

Algorithm output: normal (75 beats per minute)

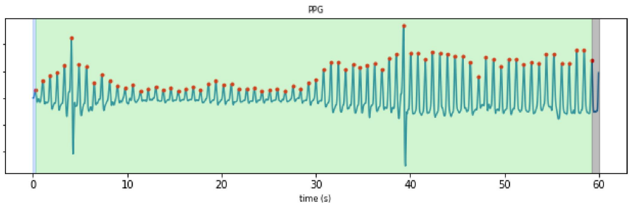

Red dot: potential heartbeat detected by the algorithm
Tachogram: time between the detected peaks (RR-interval)
Poincaré: duration of each RR-interval (RR n) in function of its preceding RR-interval (RR n-1)
Green colour: regular rhythm
Orange colour: one or more ectopic heartbeats
Red colour: atrial fibrillation
Blue: insufficient signal quality

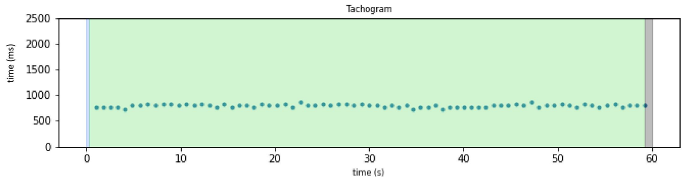

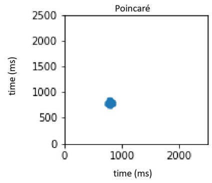

INTERPRET-AF I_PPG

* 51. How would you classify the following measurement?

- Regular rhythm
- One or more ectopic/missed heartbeats
- Atrial flutter
- Atrial fibrillation
- Unreadable
- Other (please specify)

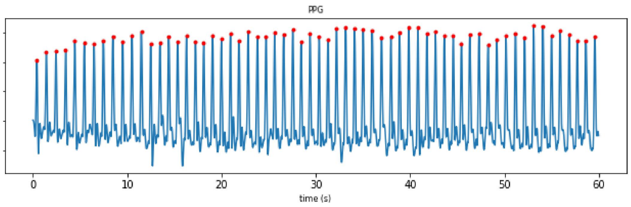

Red dot: potential heartbeat detected by the algorithm

INTERPRET-AF I_PPG

* 52. How would you classify the following measurement?

- ◯ Regular rhythm
- ◯ One or more ectopic/missed heartbeats
- ◯ Atrial flutter
- ◯ Atrial fibrillation
- ◯ Unreadable
- ◯ Other (please specify)

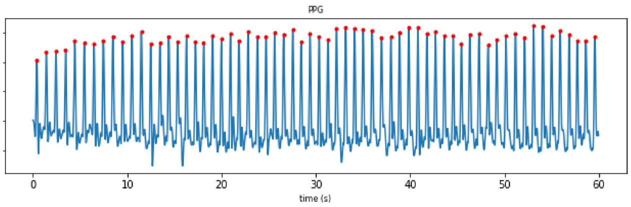

Red dot: potential heartbeat detected by the algorithm
Tachogram: time between the detected peaks (RR-interval)
Poincaré: duration of each RR-interval (RR n) in function of its preceding RR-interval (RR n-1)

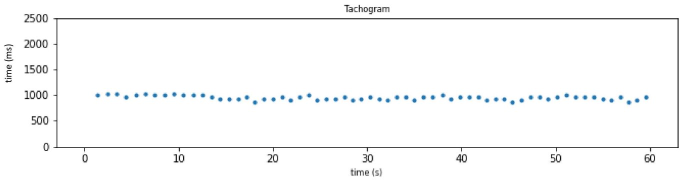

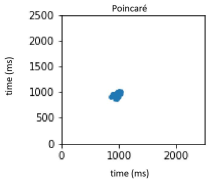

INTERPRET-AF I_PPG

* 53. How would you classify the following measurement?

- ◯ Regular rhythm
- ◯ One or more ectopic/missed heartbeats
- ◯ Atrial flutter
- ◯ Atrial fibrillation
- ◯ Unreadable
- ◯ Other (please specify)

Algorithm output: normal (63 beats per minute)

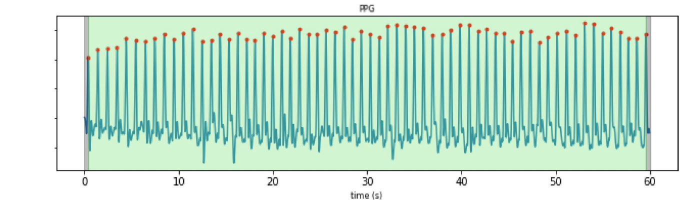

Red dot: potential heartbeat detected by the algorithm
Tachogram: time between the detected peaks (RR-interval)
Poincaré: duration of each RR-interval (RR n) in function of its preceding RR-interval (RR n-1)
Green colour: regular rhythm
Orange colour: one or more ectopic heartbeats
Red colour: atrial fibrillation
Blue: insufficient signal quality

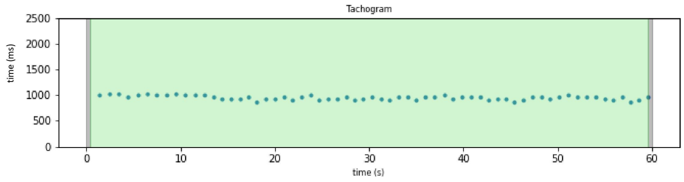

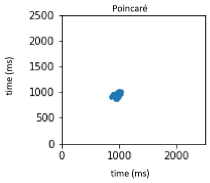

Supplement: Supplementary file 3 [file Data_Sheet_3.PDF]
