# Supplementary material for: Accuracy of Physicians Interpreting Photoplethysmography and Electrocardiography Tracings to Detect Atrial Fibrillation: INTERPRET-AF
Source: Front Cardiovasc Med. 2021 Sep 20;8:734737. doi: 10.3389/fcvm.2021.734737 (PMC8488290; doi:10.3389/fcvm.2021.734737)

INTERPRET-AF I_PPG

* 54. How would you classify the following measurement?

- Regular rhythm
- One or more ectopic/missed heartbeats
- Atrial flutter
- Atrial fibrillation
- Unreadable
- Other (please specify)

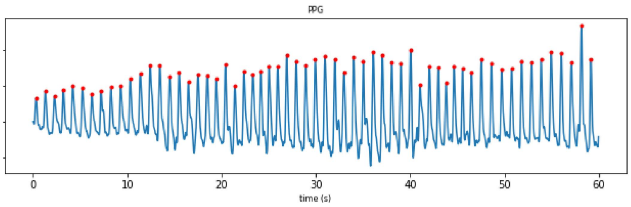

Red dot: potential heartbeat detected by the algorithm

INTERPRET-AF I_PPG

* 55. How would you classify the following measurement?

- ◯ Regular rhythm
- ◯ One or more ectopic/missed heartbeats
- ◯ Atrial flutter
- ◯ Atrial fibrillation
- ◯ Unreadable
- ◯ Other (please specify)

Red dot: potential heartbeat detected by the algorithm
Tachogram: time between the detected peaks (RR-interval)
Poincaré: duration of each RR-interval (RR n) in function of its preceding RR-interval (RR n-1)

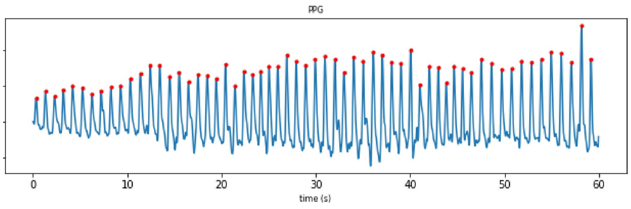

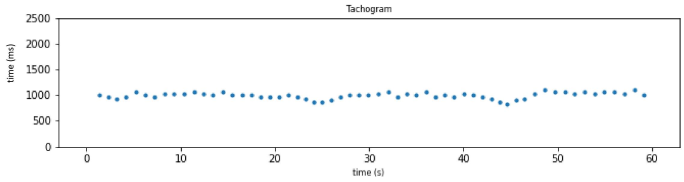

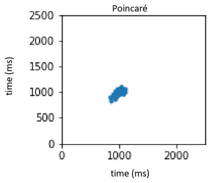

INTERPRET-AF I_PPG

* 56. How would you classify the following measurement?

- ◯ Regular rhythm
- ◯ One or more ectopic/missed heartbeats
- ◯ Atrial flutter
- ◯ Atrial fibrillation
- ◯ Unreadable
- ◯ Other (please specify)

Algorithm output: normal (93 beats per minute)

Red dot: potential heartbeat detected by the algorithm
Tachogram: time between the detected peaks (RR-interval)
Poincaré: duration of each RR-interval (RR n) in function of its preceding RR-interval (RR n-1)
Green colour: regular rhythm
Orange colour: one or more ectopic heartbeats
Red colour: atrial fibrillation
Blue: insufficient signal quality

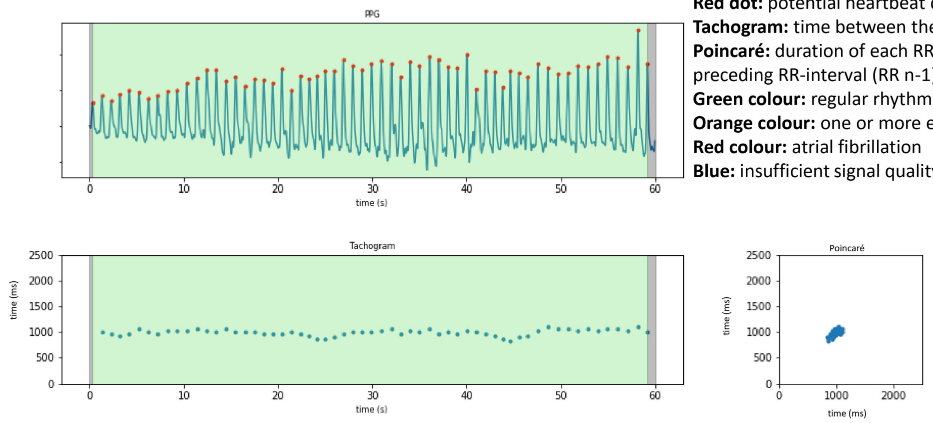

INTERPRET-AF I_PPG

* 57. How would you classify the following measurement?

- Regular rhythm
- One or more ectopic/missed heartbeats
- Atrial flutter
- Atrial fibrillation
- Unreadable
- Other (please specify)

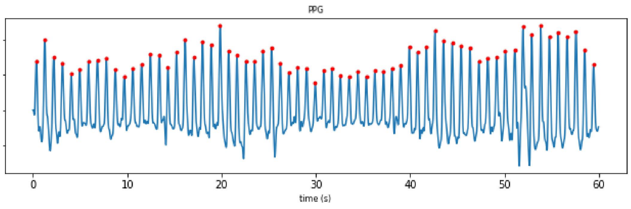

Red dot: potential heartbeat detected by the algorithm

INTERPRET-AF I_PPG

* 58. How would you classify the following measurement?

- Regular rhythm
- One or more ectopic/missed heartbeats
- Atrial flutter
- Atrial fibrillation
- Unreadable
- Other (please specify)

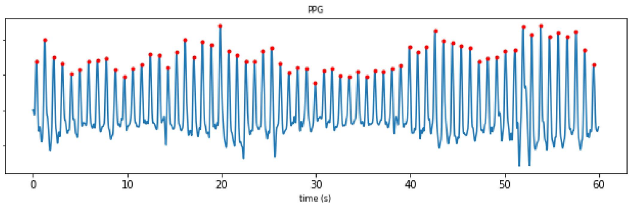

Red dot: potential heartbeat detected by the algorithm
Tachogram: time between the detected peaks (RR-interval)
Poincaré: duration of each RR-interval (RR n) in function of its preceding RR-interval (RR n-1)

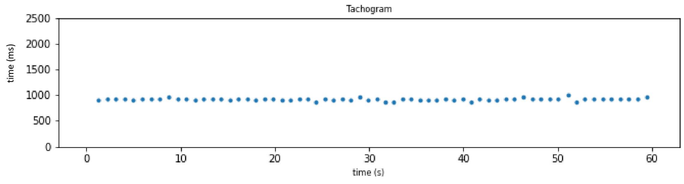

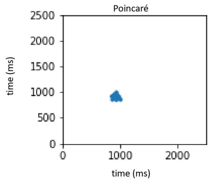

INTERPRET-AF I_PPG

* 59. How would you classify the following measurement?

- ◯ Regular rhythm
- ◯ One or more ectopic/missed heartbeats
- ◯ Atrial flutter
- ◯ Atrial fibrillation
- ◯ Unreadable
- ◯ Other (please specify)

Algorithm output: normal (77 beats per minute)

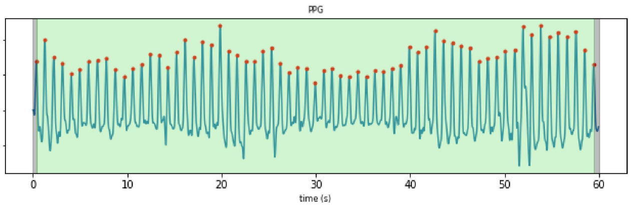

Red dot: potential heartbeat detected by the algorithm
Tachogram: time between the detected peaks (RR-interval)
Poincaré: duration of each RR-interval (RR n) in function of its preceding RR-interval (RR n-1)
Green colour: regular rhythm
Orange colour: one or more ectopic heartbeats
Red colour: atrial fibrillation
Blue: insufficient signal quality

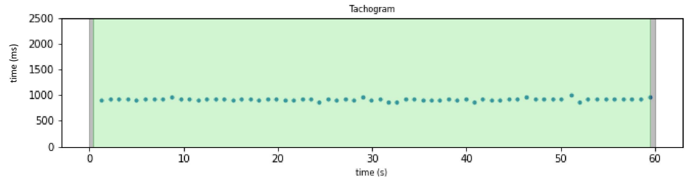

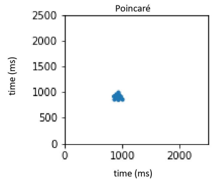

INTERPRET-AF I_PPG

* 60. How would you classify the following measurement?

- Regular rhythm
- One or more ectopic/missed heartbeats
- Atrial flutter
- Atrial fibrillation
- Unreadable
- Other (please specify)

Red dot: potential heartbeat detected by the algorithm

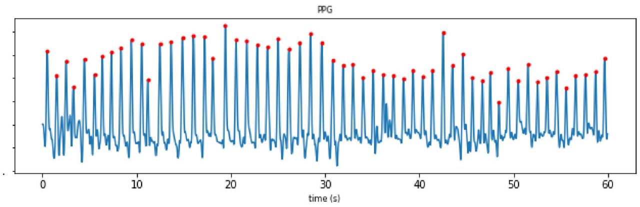

INTERPRET-AF I_PPG

* 61. How would you classify the following measurement?

- Regular rhythm
- One or more ectopic/missed heartbeats
- Atrial flutter
- Atrial fibrillation
- Unreadable
- Other (please specify)

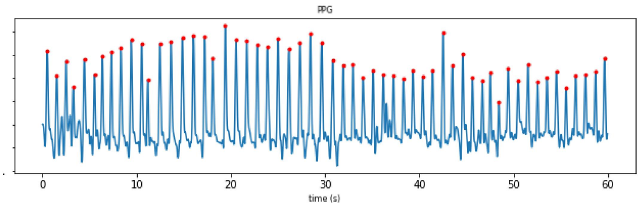

Red dot: potential heartbeat detected by the algorithm
Tachogram: time between the detected peaks (RR-interval)
Poincaré: duration of each RR-interval (RR n) in function of its preceding RR-interval (RR n-1)

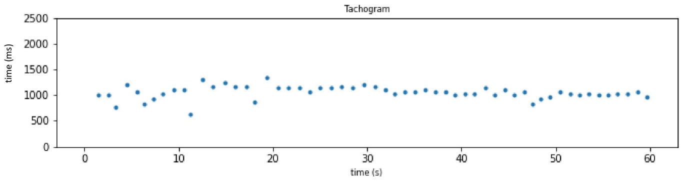

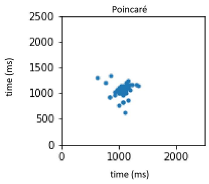

INTERPRET-AF I_PPG

* 62. How would you classify the following measurement?

- Regular rhythm
- One or more ectopic/missed heartbeats
- Atrial flutter
- Atrial fibrillation
- Unreadable
- Other (please specify)

Algorithm output: warning (57 beats per minute)

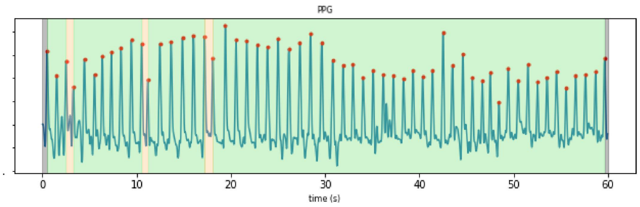

Red dot: potential heartbeat detected by the algorithm
Tachogram: time between the detected peaks (RR-interval)
Poincaré: duration of each RR-interval (RR n) in function of its preceding RR-interval (RR n-1)
Green colour: regular rhythm
Orange colour: one or more ectopic heartbeats
Red colour: atrial fibrillation
Blue: insufficient signal quality

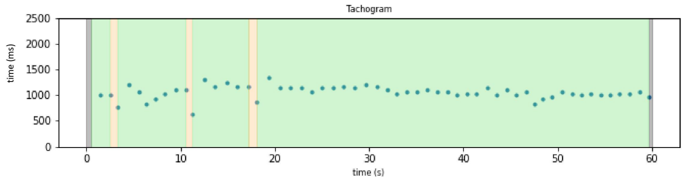

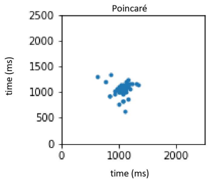

INTERPRET-AF I_PPG

* 63. How would you classify the following measurement?

- Regular rhythm
- One or more ectopic/missed heartbeats
- Atrial flutter
- Atrial fibrillation
- Unreadable
- Other (please specify)

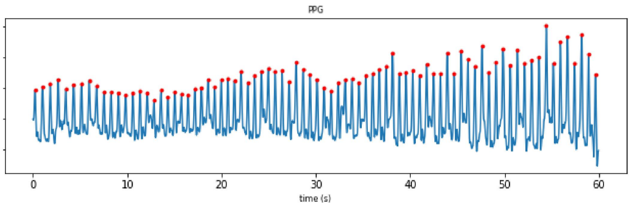

Red dot: potential heartbeat detected by the algorithm

INTERPRET-AF I_PPG

* 64. How would you classify the following measurement?

- ◯ Regular rhythm
- ◯ One or more ectopic heartbeats
- ◯ Atrial flutter
- ◯ Atrial fibrillation
- ◯ Unreadable
- ◯ Other (please specify)

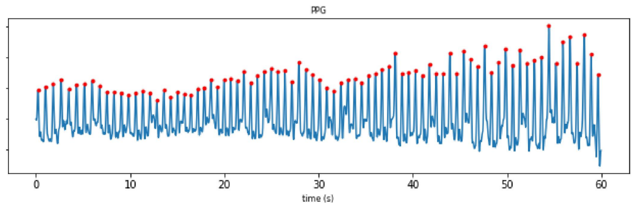

Red dot: potential heartbeat detected by the algorithm
Tachogram: time between the detected peaks (RR-interval)
Poincaré: duration of each RR-interval (RR n) in function of its preceding RR-interval (RR n-1)

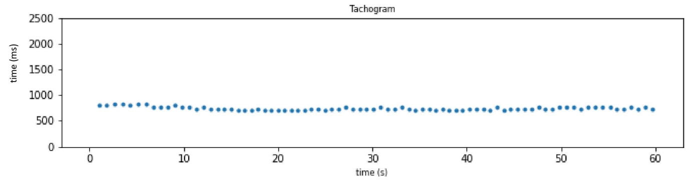

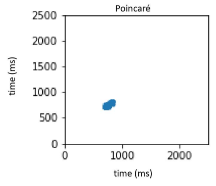

INTERPRET-AF I_PPG

* 65. How would you classify the following measurement?

- Regular rhythm
- One or more ectopic/missed heartbeats
- Atrial flutter
- Atrial fibrillation
- Unreadable
- Other (please specify)

Algorithm output: normal (81 beats per minute)

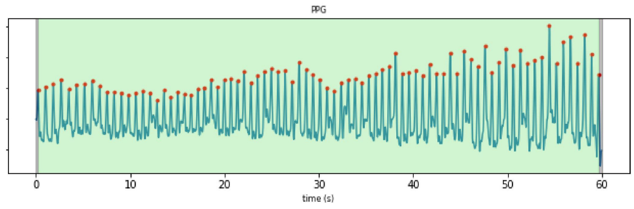

Red dot: potential heartbeat detected by the algorithm
Tachogram: time between the detected peaks (RR-interval)
Poincaré: duration of each RR-interval (RR n) in function of its preceding RR-interval (RR n-1)
Green colour: regular rhythm
Orange colour: one or more ectopic heartbeats
Red colour: atrial fibrillation
Blue: insufficient signal quality

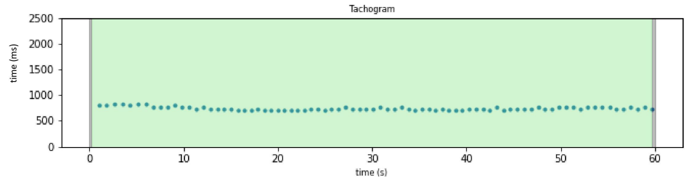

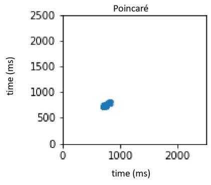

INTERPRET-AF I_PPG

* 66. How would you classify the following measurement?

- Regular rhythm
- One or more ectopic/missed heartbeats
- Atrial flutter
- Atrial fibrillation
- Unreadable
- Other (please specify)

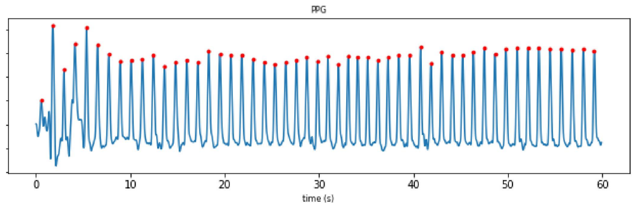

Red dot: potential heartbeat detected by the algorithm

INTERPRET-AF I_PPG

* 67. How would you classify the following measurement?

- Regular rhythm
- One or more ectopic/missed heartbeats
- Atrial flutter
- Atrial fibrillation
- Unreadable
- Other (please specify)

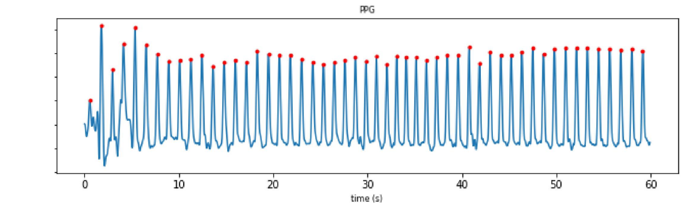

Red dot: potential heartbeat detected by the algorithm
Tachogram: time between the detected peaks (RR-interval)
Poincaré: duration of each RR-interval (RR n) in function of its preceding RR-interval (RR n-1)

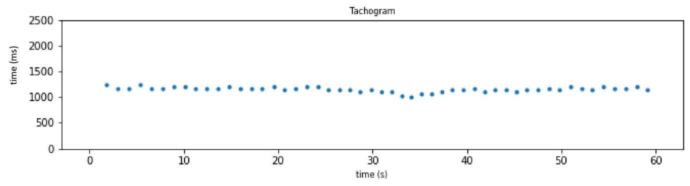

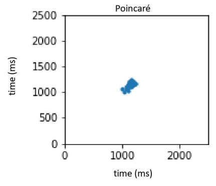

INTERPRET-AF I_PPG

* 68. How would you classify the following measurement?

- ◯ Regular rhythm
- ◯ One or more ectopic/missed heartbeats
- ◯ Atrial flutter
- ◯ Atrial fibrillation
- ◯ Unreadable
- ◯ Other (please specify)

Algorithm output: normal (52 beats per minute)

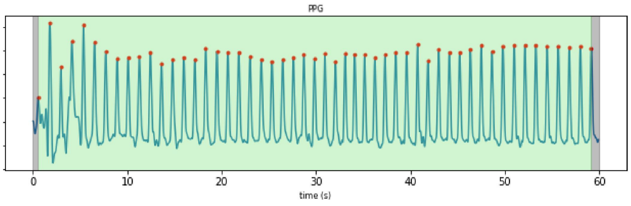

Red dot: potential heartbeat detected by the algorithm
Tachogram: time between the detected peaks (RR-interval)
Poincaré: duration of each RR-interval (RR n) in function of its preceding RR-interval (RR n-1)
Green colour: regular rhythm
Orange colour: one or more ectopic heartbeats
Red colour: atrial fibrillation
Blue: insufficient signal quality

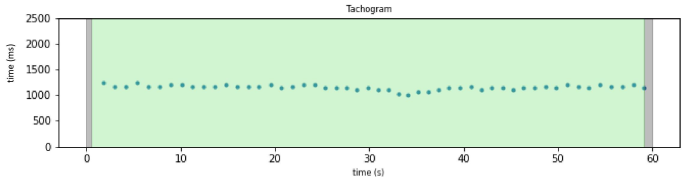

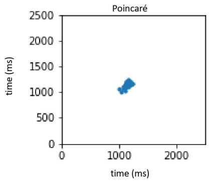

INTERPRET-AF I_PPG

* 69. How would you classify the following measurement?

- Regular rhythm
- One or more ectopic/missed heartbeats
- Atrial flutter
- Atrial fibrillation
- Unreadable
- Other (please specify)

Red dot: potential heartbeat detected by the algorithm

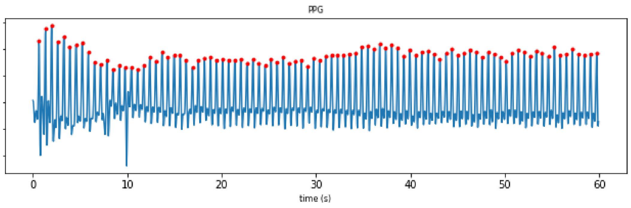

INTERPRET-AF I_PPG

* 70. How would you classify the following measurement?

- ◯ Regular rhythm
- ◯ One or more ectopic/missed heartbeats
- ◯ Atrial flutter
- ◯ Atrial fibrillation
- ◯ Unreadable
- ◯ Other (please specify)

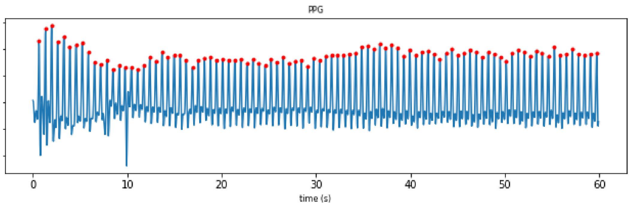

Red dot: potential heartbeat detected by the algorithm
Tachogram: time between the detected peaks (RR-interval)
Poincaré: duration of each RR-interval (RR n) in function of its preceding RR-interval (RR n-1)

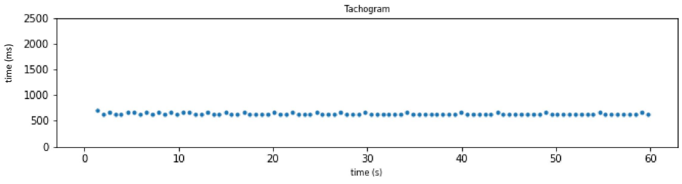

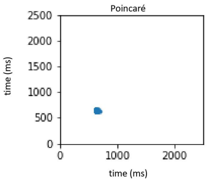

INTERPRET-AF I_PPG

* 71. How would you classify the following measurement?

- Regular rhythm
- One or more ectopic/missed heartbeats
- Atrial flutter
- Atrial fibrillation
- Unreadable
- Other (please specify)

Algorithm output: normal (93 beats per minute)

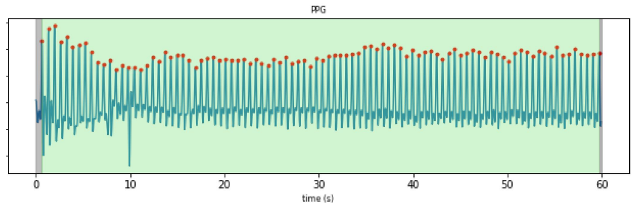

Red dot: potential heartbeat detected by the algorithm
Tachogram: time between the detected peaks (RR-interval)
Poincaré: duration of each RR-interval (RR n) in function of its preceding RR-interval (RR n-1)
Green colour: regular rhythm
Orange colour: one or more ectopic heartbeats
Red colour: atrial fibrillation
Blue: insufficient signal quality

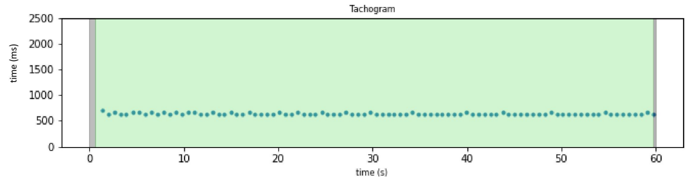

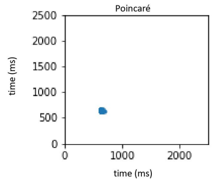

INTERPRET-AF I_PPG

* 72. How would you classify the following measurement?

- Regular rhythm
- One or more ectopic/missed heartbeats
- Atrial flutter
- Atrial fibrillation
- Unreadable
- Other (please specify)

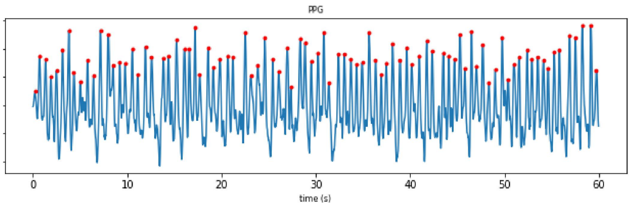

Red dot: potential heartbeat detected by the algorithm

INTERPRET-AF I_PPG

* 73. How would you classify the following measurement?

- Regular rhythm
- One or more ectopic/missed heartbeats
- Atrial flutter
- Atrial fibrillation
- Unreadable
- Other (please specify)

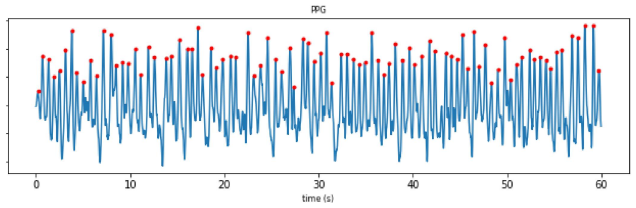

Red dot: potential heartbeat detected by the algorithm
Tachogram: time between the detected peaks (RR-interval)
Poincaré: duration of each RR-interval (RR n) in function of its preceding RR-interval (RR n-1)

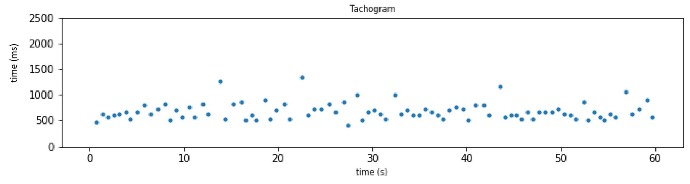

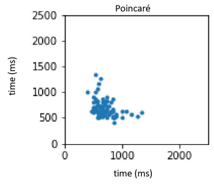

INTERPRET-AF I_PPG

* 74. How would you classify the following measurement?

- Regular rhythm
- One or more ectopic/missed heartbeats
- Atrial flutter
- Atrial fibrillation
- Unreadable
- Other (please specify)

Algorithm output: urgent (88 beats per minute)

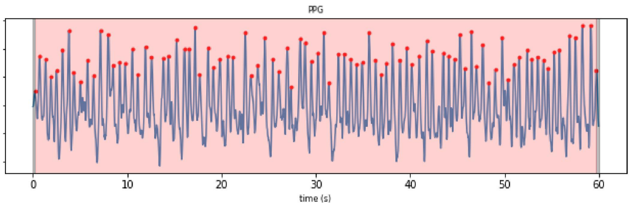

Red dot: potential heartbeat detected by the algorithm
Tachogram: time between the detected peaks (RR-interval)
Poincaré: duration of each RR-interval (RR n) in function of its preceding RR-interval (RR n-1)
Green colour: regular rhythm
Orange colour: one or more ectopic heartbeats
Red colour: atrial fibrillation
Blue: insufficient signal quality

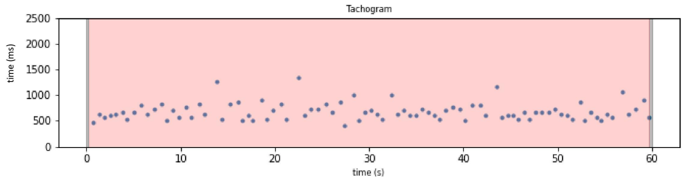

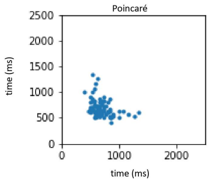

INTERPRET-AF I_PPG

* 75. How would you classify the following measurement?

- Regular rhythm
- One or more ectopic/missed heartbeats
- Atrial flutter
- Atrial fibrillation
- Unreadable
- Other (please specify)

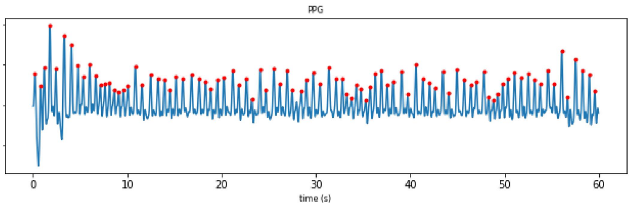

Red dot: potential heartbeat detected by the algorithm

INTERPRET-AF I_PPG

* 76. How would you classify the following measurement?

- Regular rhythm
- One or more ectopic/missed heartbeats
- Atrial flutter
- Atrial fibrillation
- Unreadable
- Other (please specify)

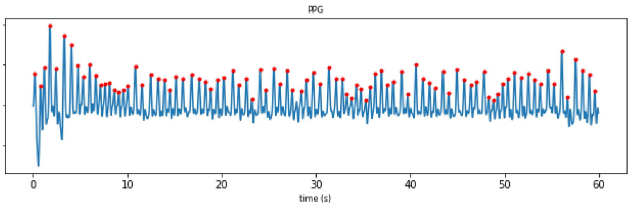

Red dot: potential heartbeat detected by the algorithm
Tachogram: time between the detected peaks (RR-interval)
Poincaré: duration of each RR-interval (RR n) in function of its preceding RR-interval (RR n-1)

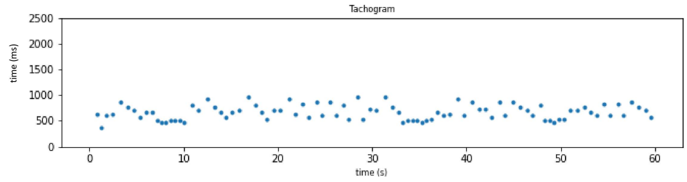

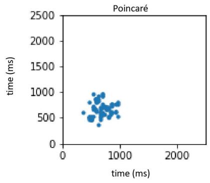

INTERPRET-AF I_PPG

* 77. How would you classify the following measurement?

- Regular rhythm
- One or more ectopic/missed heartbeats
- Atrial flutter
- Atrial fibrillation
- Unreadable
- Other (please specify)

Algorithm output: urgent (90 beats per minute)

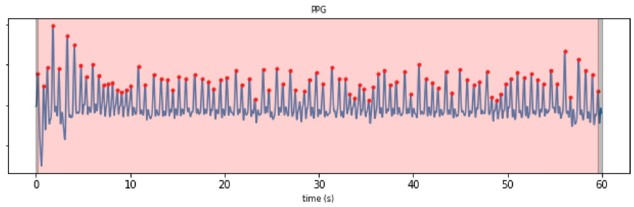

Red dot: potential heartbeat detected by the algorithm
Tachogram: time between the detected peaks (RR-interval)
Poincaré: duration of each RR-interval (RR n) in function of its preceding RR-interval (RR n-1)
Green colour: regular rhythm
Orange colour: one or more ectopic heartbeats
Red colour: atrial fibrillation
Blue: insufficient signal quality

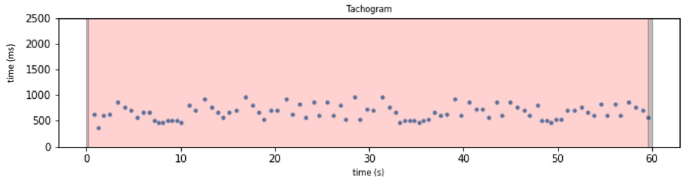

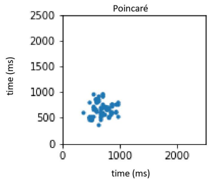

INTERPRET-AF I_PPG

* 78. How would you classify the following measurement?

- ◯ Regular rhythm
- ◯ One or more ectopic/missed heartbeats
- ◯ Atrial flutter
- ◯ Atrial fibrillation
- ◯ Unreadable
- ◯ Other (please specify)

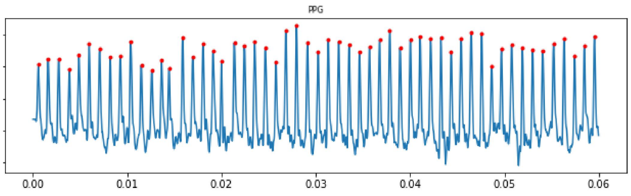

Red dot: potential heartbeat detected by the algorithm

INTERPRET-AF I_PPG

* 79. How would you classify the following measurement?

- Regular rhythm
- One or more ectopic/missed heartbeats
- Atrial flutter
- Atrial fibrillation
- Unreadable
- Other (please specify)

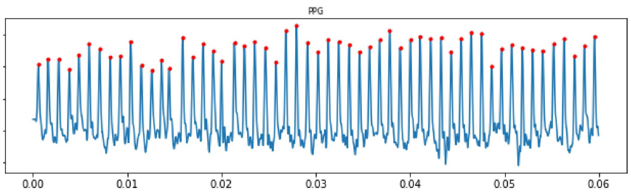

Red dot: potential heartbeat detected by the algorithm
Tachogram: time between the detected peaks (RR-interval)
Poincaré: duration of each RR-interval (RR n) in function of its preceding RR-interval (RR n-1)

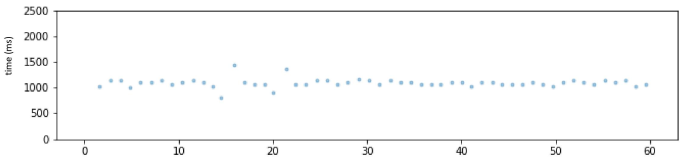

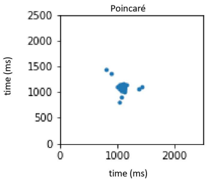

INTERPRET-AF I_PPG

* 80. How would you classify the following measurement?

- ◯ Regular rhythm
- ◯ One or more ectopic/missed heartbeats
- ◯ Atrial flutter
- ◯ Atrial fibrillation
- ◯ Unreadable
- ◯ Other (please specify)

Algorithm output: normal (55 beats per minute)

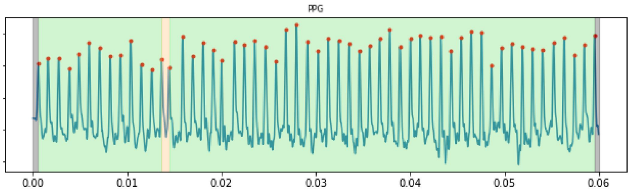

Red dot: potential heartbeat detected by the algorithm
Tachogram: time between the detected peaks (RR-interval)
Poincaré: duration of each RR-interval (RR n) in function of its preceding RR-interval (RR n-1)
Green colour: regular rhythm
Orange colour: one or more ectopic heartbeats
Red colour: atrial fibrillation
Blue: insufficient signal quality

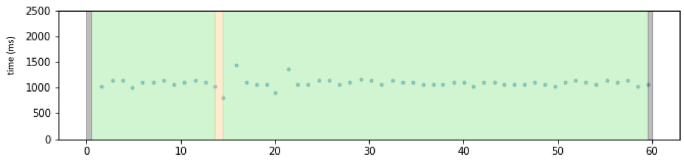

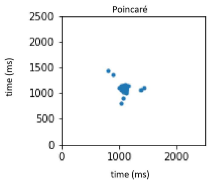

INTERPRET-AF I_PPG

* 81. How would you classify the following measurement?

- Regular rhythm
- One or more ectopic/missed heartbeats
- Atrial flutter
- Atrial fibrillation
- Unreadable
- Other (please specify)

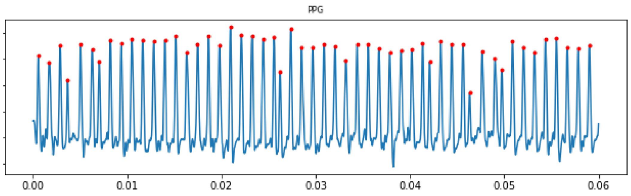

Red dot: potential heartbeat detected by the algorithm

INTERPRET-AF I_PPG

* 82. How would you classify the following measurement?

- ◯ Regular rhythm
- ◯ One or more ectopic/missed heartbeats
- ◯ Atrial flutter
- ◯ Atrial fibrillation
- ◯ Unreadable
- ◯ Other (please specify)

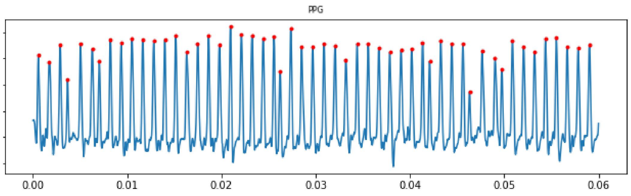

Red dot: potential heartbeat detected by the algorithm
Tachogram: time between the detected peaks (RR-interval)
Poincaré: duration of each RR-interval (RR n) in function of its preceding RR-interval (RR n-1)

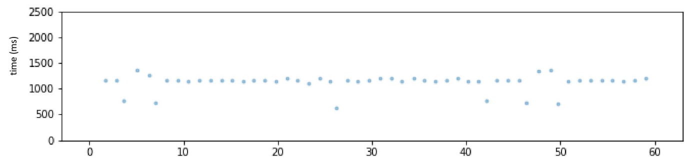

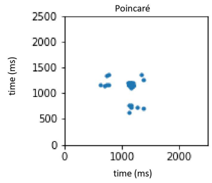

INTERPRET-AF I_PPG

* 83. How would you classify the following measurement?

- Regular rhythm
- One or more ectopic/missed heartbeats
- Atrial flutter
- Atrial fibrillation
- Unreadable
- Other (please specify)

Algorithm output: warning (53 beats per minute)

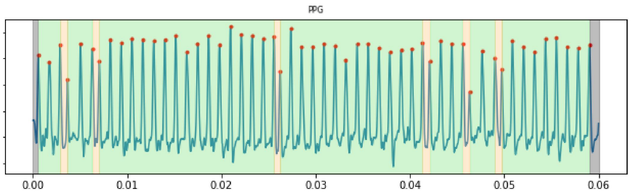

Red dot: potential heartbeat detected by the algorithm
Tachogram: time between the detected peaks (RR-interval)
Poincaré: duration of each RR-interval (RR n) in function of its preceding RR-interval (RR n-1)
Green colour: regular rhythm
Orange colour: one or more ectopic heartbeats
Red colour: atrial fibrillation
Blue: insufficient signal quality

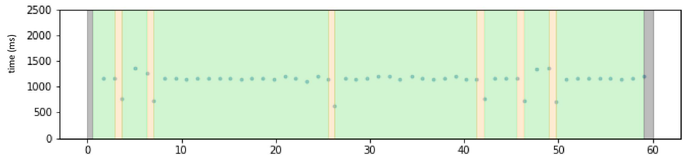

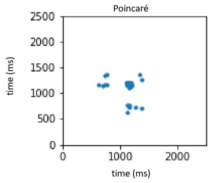

INTERPRET-AF I_PPG

* 84. How would you classify the following measurement?

- Regular rhythm
- One or more ectopic/missed heartbeats
- Atrial flutter
- Atrial fibrillation
- Unreadable
- Other (please specify)

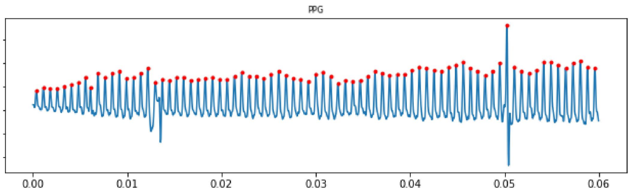

Red dot: potential heartbeat detected by the algorithm

INTERPRET-AF I_PPG

* 85. How would you classify the following measurement?

- ◯ Regular rhythm
- ◯ One or more ectopic/missed heartbeats
- ◯ Atrial flutter
- ◯ Atrial fibrillation
- ◯ Unreadable
- ◯ Other (please specify)

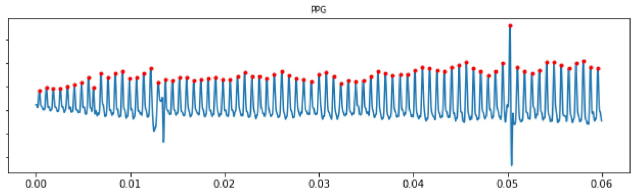

Red dot: potential heartbeat detected by the algorithm
Tachogram: time between the detected peaks (RR-interval)
Poincaré: duration of each RR-interval (RR n) in function of its preceding RR-interval (RR n-1)

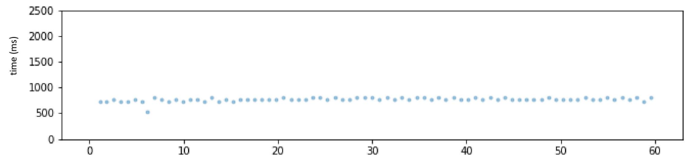

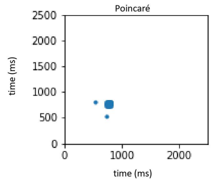

INTERPRET-AF I_PPG

* 86. How would you classify the following measurement?

- ○ Regular rhythm
- ○ One or more ectopic/missed heartbeats
- ○ Atrial flutter
- ○ Atrial fibrillation
- ○ Unreadable
- ○ Other (please specify)

Algorithm output: normal (78 beats per minute)

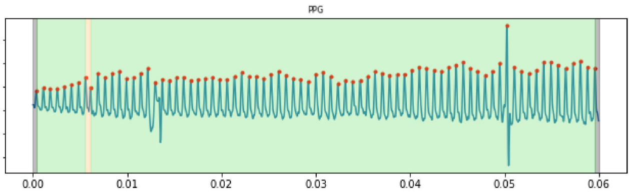

Red dot: potential heartbeat detected by the algorithm
Tachogram: time between the detected peaks (RR-interval)
Poincaré: duration of each RR-interval (RR n) in function of its preceding RR-interval (RR n-1)
Green colour: regular rhythm
Orange colour: one or more ectopic heartbeats
Red colour: atrial fibrillation
Blue: insufficient signal quality

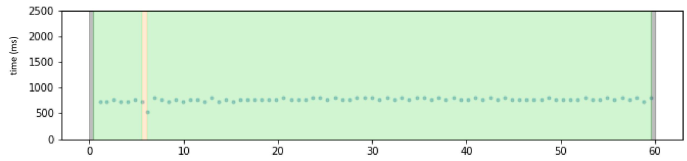

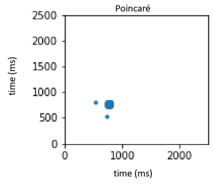

INTERPRET-AF I_PPG

* 87. How would you classify the following measurement?

- Regular rhythm
- One or more ectopic/missed heartbeats
- Atrial flutter
- Atrial fibrillation
- Unreadable
- Other (please specify)

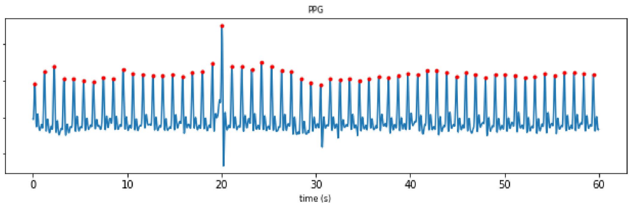

Red dot: potential heartbeat detected by the algorithm

INTERPRET-AF I_PPG

* 88. How would you classify the following measurement?

- Regular rhythm
- One or more ectopic/missed heartbeats
- Atrial flutter
- Atrial fibrillation
- Unreadable
- Other (please specify)

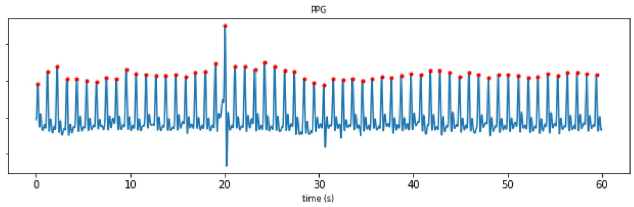

Red dot: potential heartbeat detected by the algorithm
Tachogram: time between the detected peaks (RR-interval)
Poincaré: duration of each RR-interval (RR n) in function of its preceding RR-interval (RR n-1)

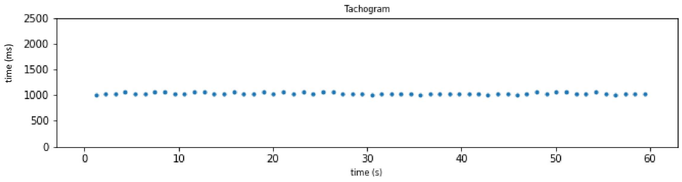

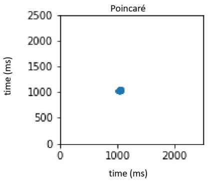

INTERPRET-AF I_PPG

* 89. How would you classify the following measurement?

- ◯ Regular rhythm
- ◯ One or more ectopic/missed heartbeats
- ◯ Atrial flutter
- ◯ Atrial fibrillation
- ◯ Unreadable
- ◯ Other (please specify)

Algorithm output: normal (58 beats per minute)

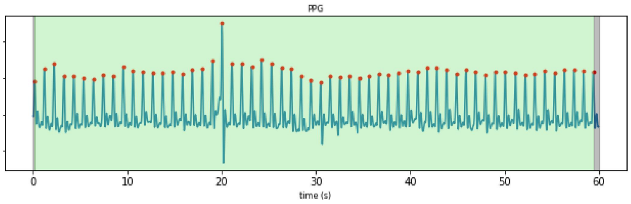

Red dot: potential heartbeat detected by the algorithm
Tachogram: time between the detected peaks (RR-interval)
Poincaré: duration of each RR-interval (RR n) in function of its preceding RR-interval (RR n-1)
Green colour: regular rhythm
Orange colour: one or more ectopic heartbeats
Red colour: atrial fibrillation
Blue: insufficient signal quality

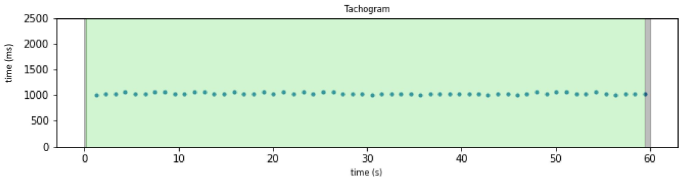

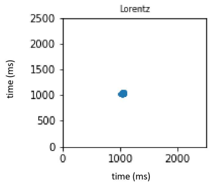

INTERPRET-AF I_PPG

* 90. How would you classify the following measurement?

- Regular rhythm
- One or more ectopic/missed heartbeats
- Atrial flutter
- Atrial fibrillation
- Unreadable
- Other (please specify)

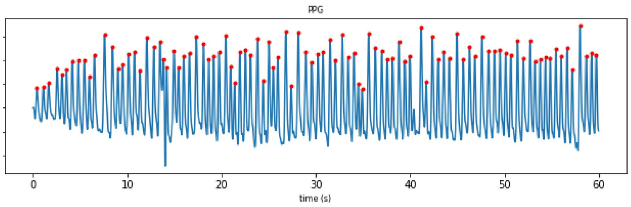

Red dot: potential heartbeat detected by the algorithm

INTERPRET-AF I_PPG

* 91. How would you classify the following measurement?

- ◯ Regular rhythm
- ◯ One or more ectopic/missed heartbeats
- ◯ Atrial flutter
- ◯ Atrial fibrillation
- ◯ Unreadable
- ◯ Other (please specify)

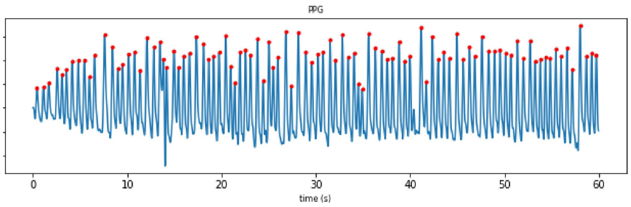

Red dot: potential heartbeat detected by the algorithm
Tachogram: time between the detected peaks (RR-interval)
Poincaré: duration of each RR-interval (RR n) in function of its preceding RR-interval (RR n-1)

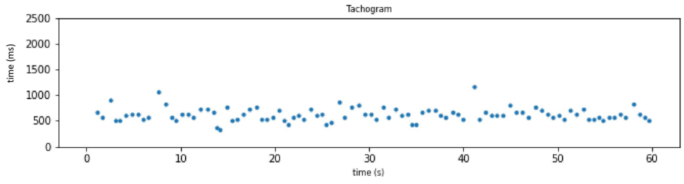

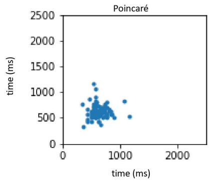

INTERPRET-AF I_PPG

* 92. How would you classify the following measurement?

- Regular rhythm
- One or more ectopic/missed heartbeats
- Atrial flutter
- Atrial fibrillation
- Unreadable
- Other (please specify)

Algorithm output: urgent (96 beats per minute)

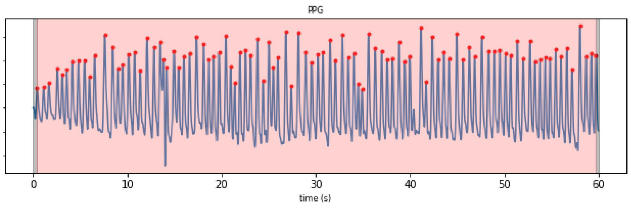

Red dot: potential heartbeat detected by the algorithm
Tachogram: time between the detected peaks (RR-interval)
Poincaré: duration of each RR-interval (RR n) in function of its preceding RR-interval (RR n-1)
Green colour: regular rhythm
Orange colour: one or more ectopic heartbeats
Red colour: atrial fibrillation
Blue: insufficient signal quality

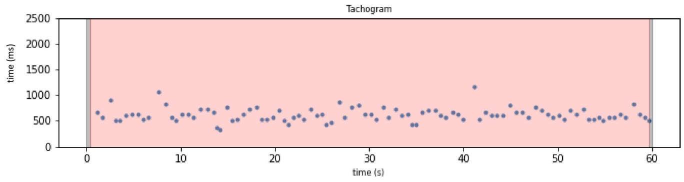

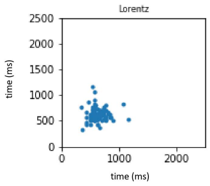

INTERPRET-AF I_PPG

* 93. How would you classify the following measurement?

- Regular rhythm
- One or more ectopic/missed heartbeats
- Atrial flutter
- Atrial fibrillation
- Unreadable
- Other (please specify)

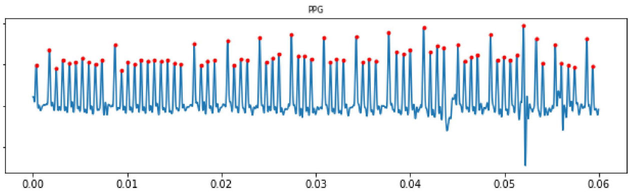

Red dot: potential heartbeat detected by the algorithm

INTERPRET-AF I_PPG

* 94. How would you classify the following measurement?

- ◯ Regular rhythm
- ◯ One or more ectopic/missed heartbeats
- ◯ Atrial flutter
- ◯ Atrial fibrillation
- ◯ Unreadable
- ◯ Other (please specify)

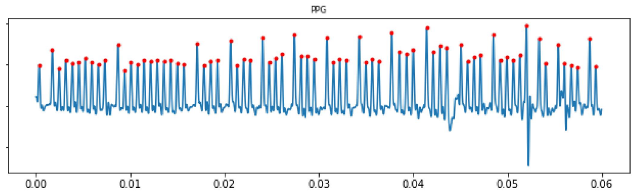

Red dot: potential heartbeat detected by the algorithm
Tachogram: time between the detected peaks (RR-interval)
Poincaré: duration of each RR-interval (RR n) in function of its preceding RR-interval (RR n-1)

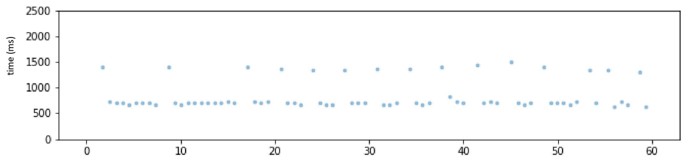

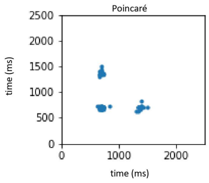

INTERPRET-AF I_PPG

* 95. How would you classify the following measurement?

- ◯ Regular rhythm
- ◯ One or more ectopic/missed heartbeats
- ◯ Atrial flutter
- ◯ Atrial fibrillation
- ◯ Unreadable
- ◯ Other (please specify)

Algorithm output: warning (71 beats per minute)

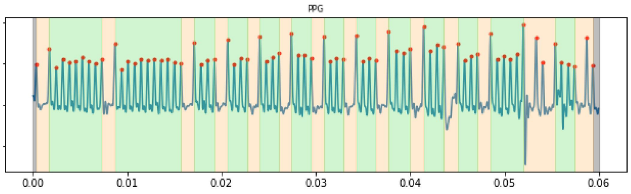

Red dot: potential heartbeat detected by the algorithm
Tachogram: time between the detected peaks (RR-interval)
Poincaré: duration of each RR-interval (RR n) in function of its preceding RR-interval (RR n-1)
Green colour: regular rhythm
Orange colour: one or more ectopic heartbeats
Red colour: atrial fibrillation
Blue: insufficient signal quality

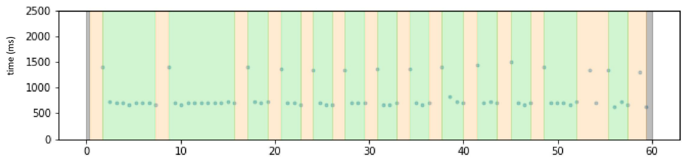

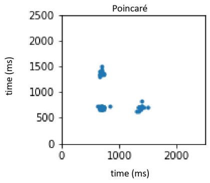

INTERPRET-AF I_PPG

* 96. How would you classify the following measurement?

- Regular rhythm
- One or more ectopic/missed heartbeats
- Atrial flutter
- Atrial fibrillation
- Unreadable
- Other (please specify)

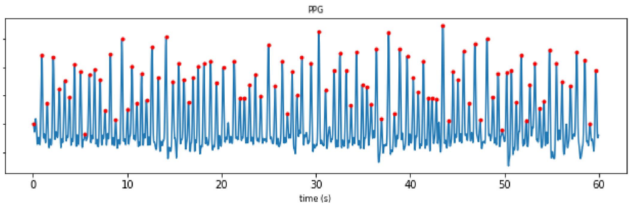

Red dot: potential heartbeat detected by the algorithm

INTERPRET-AF I_PPG

* 97. How would you classify the following measurement?

- Regular rhythm
- One or more ectopic/missed heartbeats
- Atrial flutter
- Atrial fibrillation
- Unreadable
- Other (please specify)

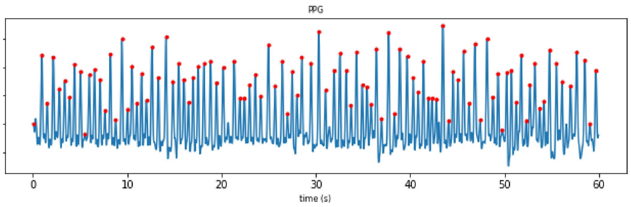

Red dot: potential heartbeat detected by the algorithm
Tachogram: time between the detected peaks (RR-interval)
Poincaré: duration of each RR-interval (RR n) in function of its preceding RR-interval (RR n-1)

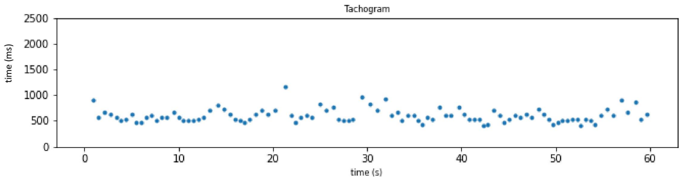

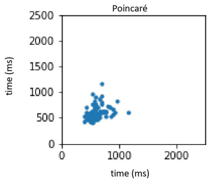

INTERPRET-AF I_PPG

* 98. How would you classify the following measurement?

- Regular rhythm
- One or more ectopic/missed heartbeats
- Atrial flutter
- Atrial fibrillation
- Unreadable
- Other (please specify)

Algorithm output: urgent (100 beats per minute)

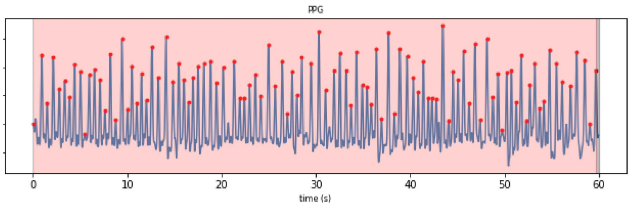

Red dot: potential heartbeat detected by the algorithm
Tachogram: time between the detected peaks (RR-interval)
Poincaré: duration of each RR-interval (RR n) in function of its preceding RR-interval (RR n-1)
Green colour: regular rhythm
Orange colour: one or more ectopic heartbeats
Red colour: atrial fibrillation
Blue: insufficient signal quality

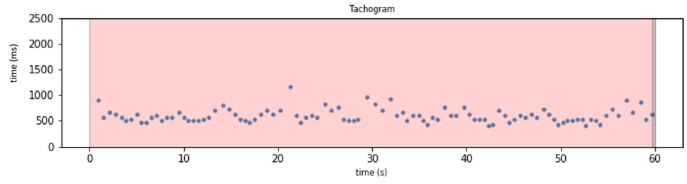

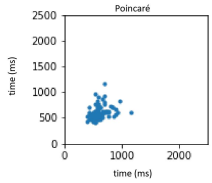

Supplement: Supplementary file 4 [file Data_Sheet_4.PDF]
